# Temporal transcriptomic changes in the THY-Tau22 mouse model of tauopathy display cell type- and sex-specific differences

**DOI:** 10.1186/s40478-025-02013-z

**Published:** 2025-05-07

**Authors:** Muhammad Ali, Pierre Garcia, Laetitia P. Lunkes, Alessia Sciortino, Melanie H. Thomas, Tony Heurtaux, Kamil Grzyb, Rashi Halder, Alexander Skupin, Luc Buée, David Blum, Manuel Buttini, Enrico Glaab

**Affiliations:** 1 Luxembourg Centre for Systems Biomedicine (LCSB), University of Luxembourg, 7 Avenue des Hauts Fourneaux, Esch-sur-Alzette, L-4362 Luxembourg; 2 Department of Life Sciences and Medicine (DLSM), University of Luxembourg, 6 Avenue du Swing, Belvaux, L-4367 Luxembourg; 3 Lille Neuroscience & Cognition, University of Lille, Inserm, CHU Lille, Alzheimer & Tauopathies, LabEx DISTALZ, Lille, UMR-S1172 France

**Keywords:** Alzheimer's disease, Tauopathy, THY-Tau22 mouse model, Sex differences, Age differences, Single-cell RNA sequencing, Transcriptomics

## Abstract

**Background:**

Tauopathies, including Alzheimer’s disease (AD) and frontotemporal dementia (FTD), display sex-specific differences in prevalence and progression, but the underlying molecular mechanisms remain unclear. Single-cell transcriptomic analysis of animal models can reveal how AD pathology affects different cell types across sex and age.

**Objective:**

To understand sex-specific and sex-dimorphic transcriptomic changes in different cell types and their age-dependence in the THY-Tau22 mouse model of AD-linked tauopathy.

**Methods:**

We applied single-cell RNA sequencing (scRNA-seq) to cortical tissue from male and female THY-Tau22 and wild-type mice at 17 months of age, when they had prominent tau inclusion pathology, and compared the results with corresponding data previously obtained at 7 months of age. Using differential statistical analysis for individual genes, pathways, and gene regulatory networks, we identified sex-specific, sex-dimorphic, and sex-neutral changes, and looked at how they evolved over age. To validate the most robust findings across distinct mouse models and species, the results were compared with cortical scRNA-seq data from the transgenic hAPP-based Tg2576 mouse model and human AD.

**Results:**

We identified several significant sex-specific and sex-dimorphic differentially expressed genes in neurons, microglia, astrocytes and oligodendrocytes, including both cross-sectional changes and alterations from 7 months to 17 months of age. Key pathways affected in a sex-dependent manner across age included neurotransmitter signaling, RNA processing and splicing, stress response pathways, and protein degradation pathways. In addition, network analysis revealed the AD-associated genes *Clu*, *Mbp*, *Fos* and *Junb* as relevant regulatory hubs. Analysis of age-dependent changes highlighted genes and pathways associated with inflammatory response (*Malat1*, *Cx3cr1*), protein homeostasis (*Cst3*), and myelin maintenance (*Plp1*, *Cldn11*, *Mal*) that showed consistent sex-dependent changes as the THY-Tau22 mice aged. Multiple genes with established implications in AD, including the long non-coding RNA gene *Malat1*, displayed concordant sex-specific changes in mouse models and human AD.

**Conclusions:**

This study provides a comprehensive single-cell transcriptomic characterization of sex-linked and age-dependent changes in the THY-Tau22 tauopathy model, revealing new insights into the interplay between age-dependent AD-like pathologies and sex. The identified sex-specific changes and their conservation across models and human AD highlight molecular targets for further preclinical investigation of sex-specific therapeutic strategies in AD.

**Supplementary Information:**

The online version contains supplementary material available at 10.1186/s40478-025-02013-z.

## Introduction

Alzheimer’s disease (AD) is a heterogeneous, progressive disorder with varied clinical presentations, rates of progression, and treatment responses. Biological sex is a key factor contributing to this diversity, with women showing an age-adjusted higher prevalence compared to men, comprising nearly two-thirds of individuals living with AD in the United States [1, 2]. Sex differences extend beyond prevalence; women with AD often experience faster cognitive decline and more severe neuropathological changes, while males tend to die faster with AD [3, 4] and have lower brain resilience to tau pathology [5]. Furthermore, sex differences have been observed in treatment effectiveness and risk factor impact [6, 7]. Despite established knowledge of AD hallmarks, amyloid-beta plaques and neurofibrillary tangles, the molecular mechanisms driving sex-specific differences remain poorly understood, presenting a significant barrier to developing personalized therapeutic strategies.

High-throughput single-cell RNA sequencing (scRNA-seq) technologies have significantly improved our ability to investigate complex cellular systems at unprecedented resolution, providing insights into cell type-specific changes and heterogeneity within tissues. In AD research, scRNA-seq has become a key tool for mapping cellular and molecular alterations associated with disease progression. Several studies have used scRNA-seq to investigate AD-related changes in human *post-mortem* brain tissue and animal models. For instance, Mathys et al. performed single-nucleus RNA-seq on human prefrontal cortex samples [8], revealing cell type-specific transcriptomic changes associated with AD pathology. Similarly, Grubman et al. used single-cell sequencing to identify altered cellular pathways in human AD brain tissue [9]. In animal models, studies such as the one by Keren-Shaul et al. [10] have employed scRNA-seq to characterize microglial states in mouse models of AD-like pathology. Building on this single-cell approach, our previous work has investigated cross-sectional sex-dependent changes at early time points in the THY-Tau22 and Tg2576 models [11, 12]. However, while these studies have provided new insights into AD- and tau-related transcriptomic changes, the potential impact of sex on these alterations at different ages has not been sufficiently addressed.

The THY-Tau22 mouse model, which over-expresses human 4-repeat tau with G272V and P301S mutations that were originally identified in frontotemporal dementia (FTD) patients [13], offers a valuable tool for studying mechanisms relevant to tauopathies, such as FTD and AD [14]. This model recapitulates several key features observed in tauopathies, including progressive cognitive decline, synaptic dysfunction, and tau pathology, without the confounding effects of motor deficits often seen in other transgenic lines [15]. By comprehensively studying scRNA-seq data for THY-Tau22 mice and their wild-type littermates at 17 months, and contrasting them to our previous analysis of 7-month old mice [12], we characterize age- and sex-dependent transcriptomic alterations associated with tau pathology. Our approach identifies sex-specific, sex-dimorphic, and sex-neutral gene expression changes across multiple cell types, including major AD-associated cell populations, such as neurons, astrocytes, microglia, and oligodendrocytes, to better understand cell type-specific responses to tau pathology in both sexes.

Our analysis reveals numerous sex-specific and sex-dimorphic differentially expressed genes across various cell types. We identify key biological pathways affected in a sex-dependent manner, both cross-sectionally and across age groups, including RNA processing and splicing, stress response pathways, neurotransmitter signaling, and protein degradation pathways. Through gene regulatory network analysis, we determine potential regulatory hubs, such as the genes *Clu*, *Mbp*, *Fos* and *Junb*, that may play central roles in mediating sex-specific responses to tau pathology. Furthermore, our age-dependent analyses identify genes showing consistent and progressive sex-dependent alterations between 7 and 17 months of age. By comparing our findings with cortical scRNA-seq data from human AD and another mouse model, the Tg2576 model of amyloid-beta pathology, we identify overlapping significant genes across different experimental settings, such as *Malat1*. Overall, this study provides new insights into the interplay between tauopathy and biological sex at the single-cell level, potentially informing future investigations of sex-specific therapeutic strategies for AD and related tauopathies.

## Materials and methods

### Ethical statement

All experimental procedures were conducted in strict accordance with European guidelines for animal research (FELASA) and received approval from the local Animal Experimentation Ethics Committee as well as oversight and approval from Luxembourg’s Ministry of Agriculture and Ministry of Health (registered under LUPA 2020/23).

### Animal model, colony maintenance, and genotyping

We used a genetically engineered tauopathy mouse model, THY-Tau22 Mice (B6.Cg-Tg(Thy1-MAPT)22Schd, MGI:3717232), characterized by the over-expression of a modified human tau protein, harboring the mutations P301S and G272V, originally identified in frontotemporal dementia (FTD) patients [14]. This mutated protein is expressed under the control of the neuronal promoter Thy1.2. As these mice age, they exhibit several hallmarks of AD, including cognitive decline, impaired synaptic function, increased astrocyte reactivity, and accumulation of tau proteins.

For the THY-Tau22 mice, the original breeding pairs were obtained from the University of Lille, France. These mice were then rederived in the mouse facility of the University of Luxembourg and bred by crossing heterozygous males with C57Bl6/J females (RRID: MGI:3028467). The animals were maintained in pathogen-free facilities, with constant access to standard mouse chow (Ssniff, # V 1534 − 300) and water. Genotyping analysis of the mice was performed as described previously [12].

### Experimental design and sample collection

**Fig. 1:**
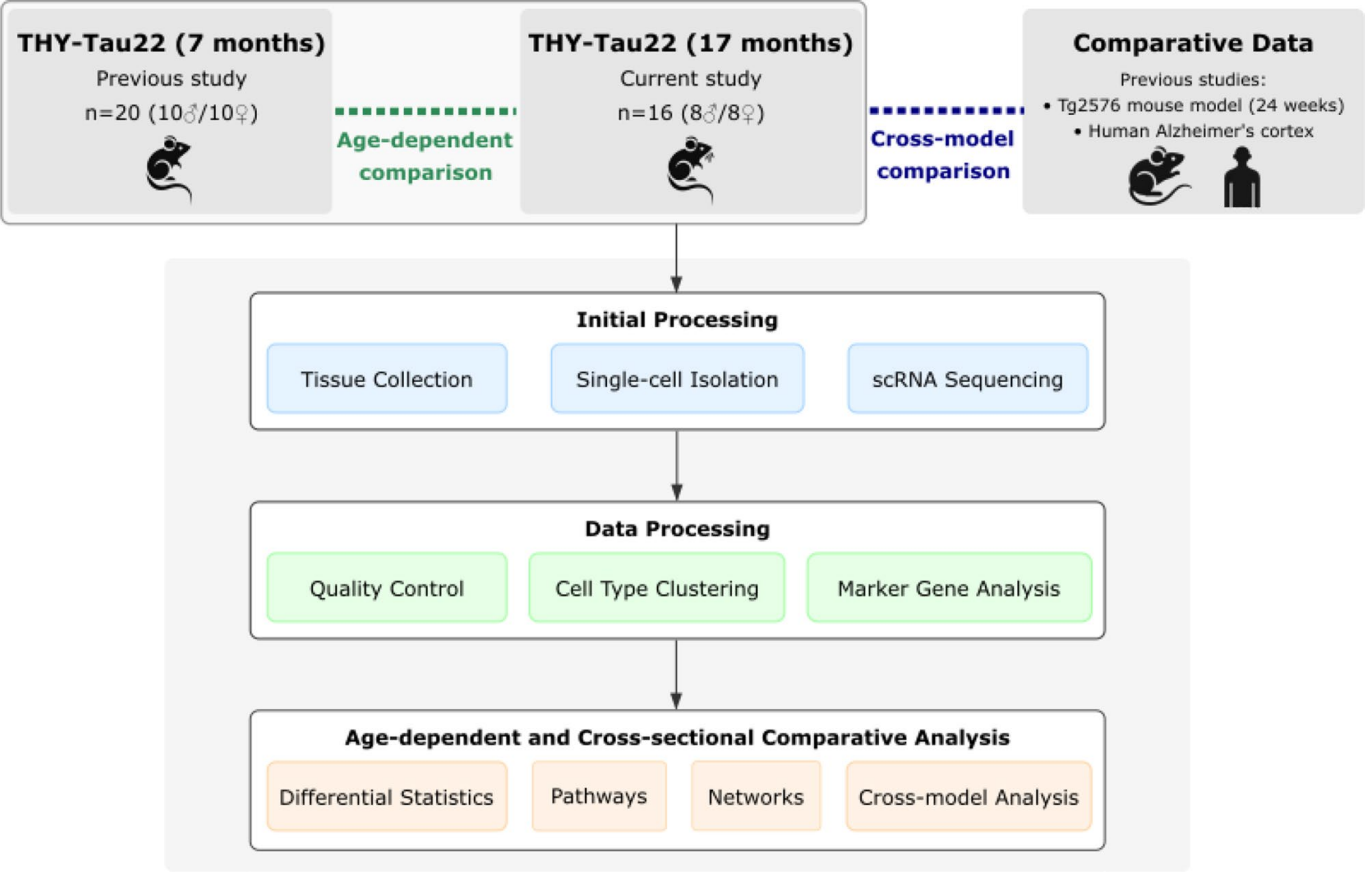
Overview of study design and analytical workflow for single cell analyses. Schematic showing the comparison of newly generated data from 17-month-old THY-Tau22 mice (*n* = 4 male THY-Tau22, *n* = 4 male wildtype, *n* = 5 female THY-Tau22, *n* = 3 female wildtype) with previously published data from 7-month-old mice (*n* = 5 per group) and data from the Tg2576 mouse model (*n* = 9 per group) and human AD cortical tissue (*n* = 3 per group). Green dashed line indicates age-dependent analyses; blue dashed line indicates cross-model comparisons with Tg2576 mice and human AD data. The workflow includes tissue processing, sequencing, data analysis, and comparative analyses

For this investigation, we studied the cerebral cortex of THY-Tau22 mice and their wild-type littermates at 17 months of age (data newly obtained for this study) and then compared the results to the same groups of mice at 7 months of age (data obtained from our previous cross-sectional study [12]). This allowed us to distinguish between early and late pathological changes and to identify and interpret age-dependent changes in this model of tauopathy. Figure 1 shows the experimental design and workflow, comparing our new data from 17-month-old mice with previous data from 7-month-old mice (age-dependent comparison), and with cortical tissue data from human AD tissue and the Tg2576 mouse model (cross-model comparison).

We used mice of both sexes, from different litters, heterozygous for the transgene and their wild-type littermates as controls, and sample sizes were optimized for each type of analysis based on their specific requirements. For single-cell transcriptomic analyses, 16 mice in total were profiled (male THY-Tau22 transgenic mice (*n* = 4), male wildtype littermates (*n* = 4), female THY-Tau22 transgenic mice (*n* = 5), and female wildtype littermates (*n* = 3)). Single-cell RNA sequencing analyses typically require fewer samples (*n* = 3–5 per group) than traditional bulk analyses due to the high number of cells profiled per sample and the resulting increased statistical power from cell-level measurements. This sample size has been validated in our previous studies with mouse models of neurodegeneration. For behavioral analyses, larger group sizes (11–17 mice per group) were used to account for higher individual variability in behavioral measurements. For neuropathological analyses, we used 8 transgenic mice and 2 wild-type controls per sex. The smaller number of controls in this case was sufficient due to their low variability and our focus on comparing male versus female transgenic mice. Sample sizes for the between-group comparisons of interest were verified using power analysis, confirming a minimum of 80% power to detect between-group changes with a type I error probability of 5% for a minimum detectable effect size of 2 (the G*Power 3.1 software was used for calculations).

After reaching the desired study ages, the animals were humanely euthanized following approved animal welfare protocols using a combination of anesthetic drugs (administered intraperitoneally with ketamine at 150 mg/kg and medetomidine at 1 mg/kg) followed by transcardial perfusion with phosphate-buffered saline (PBS) to clear blood. The brain tissue was carefully extracted and prepared for the subsequent analyses, including histological examination (one brain hemisphere) and advanced single-cell sequencing techniques (using freshly dissected cortex from the other hemisphere for DropSeq analysis).

For scRNA-seq analyses, we used the entire neocortex rather than a specific cortical subregion. This approach was deliberately chosen because: (1) the THY-Tau22 model expresses mutant tau broadly throughout the neocortex under the Thy1.2 promoter, with pathology developing across cortical regions; (2) using the whole neocortex allowed us to capture tau-associated single-cell transcriptomic changes that might occur heterogeneously across different cortical areas, providing a more comprehensive view of pathology; (3) sampling the whole neocortex provided sufficient cell numbers for robust single-cell analysis of all major cell types, including less abundant populations; and (4) the mouse neocortex is, unlike the primate neocortex, smooth and does not allow for a clear distinction between subregions upon extraction of fresh tissue.

To ensure precise sampling and minimize contamination from non-cortical regions, we used a standardized dissection protocol performed by experienced neuroscientists. After euthanasia and transcardial perfusion, the entire neocortex was carefully separated from subcortical structures, hippocampus, and olfactory bulbs using anatomical landmarks. All dissections were performed rapidly, on an iced surface, to minimize RNA degradation and with care to prevent cross-contamination.

### Behavioral assessment (Y-maze)

The spatial working memory performance of mice was assessed in the Y-maze. The Y-maze used here takes advantage of the natural exploratory behavior of the mouse. The maze consists of three identical arms with walls (40 cm long, 10 cm high, 10 cm wide), made of white opaque plastic, arranged in a Y-shape, with 120° angles between the arms around a central platform (Noldus, RRID: SCR_004074). Visual shapes (squares, circles, triangles) were depicted on the walls of the maze to allow for visual orientation and memory formation.

The test measures spontaneous alternation behavior (SAB), where the mouse is expected to remember which arm it has previously visited, and explore a new one. This ability to alternate between arms reflects working memory, as the mouse must continuously update its memory of recent choices. Memory performance is measured by calculating the percentage of spontaneous alternations, which reflects the mouse’s ability to remember and alternate between arms. A correct alternation occurs when the mouse consecutively enters each of the three arms in turn without repeating the same arm in a sequence. A high percentage of alternations indicates good working memory, as the mouse can remember which arms it has visited previously. The alternation percentage was calculated as: (number of correct alternations/total number of possible alternations (total number of arm entries minus 2)) ×100.

Seventeen-month-old THY-Tau22 (17 males, 11 females) and wild-type littermates (11 males, 13 females) were tested. The experimenter was blinded to the genotypes of the mice. At the start of the test, each mouse was placed at the end of an arm, facing outwards. The test was conducted in a controlled low-intensity light environment (10 lx). Mice were allowed to freely explore the maze during a single 5-minute session. We tracked and recorded trajectories using Ethovision software from Noldus (RRID: SCR_000441). The total number of visits to each arm and the number of correct alternations were counted manually, and the percentage of alternations was calculated using the formula above. Mice that visited fewer than 10 arms were excluded (one male and one female wild-type).

Two-way ANOVA with sex and genotype as factors was performed, followed by Sidak post hoc test to analyze the results using GraphPad Prism software (v10.3.0) (RRID: SCR_002798). P-values less than 0.05 were considered significant.

### Histology and immunohistochemistry

Staining procedures followed standard protocols [16] on randomly selected free-floating 50 µM thick sections (2 sections/mouse for THY-Tau22). Sections from 7 to 8 mice per sex were used for THY-Tau22 mice, and sections from two wild-type littermates per sex served as negative controls. Prior to staining, epitope exposure was achieved by immersing sections in 50% vol/vol formic acid (Sigma, 33015) for 5 min. The sections were then washed three times for 10 min each in PBS containing 0.1% Triton X-100 (Sigma, T8787). For permeabilization, sections were incubated in PBS with 3% H_2_O_2_ (Sigma, 31642) vol/vol and 1.5% Triton X-100 vol/vol for 30 min. After three 10-minute washes, sections were incubated in 5% BSA (Sigma, A8022) wt/vol in wash buffer for 1 h to avoid unspecific antibody binding, before being incubated overnight at RT with AT8 primary antibody (Fisher, MN1060 RRID: AB_223652) diluted 1/1000 in PBS, 0.3% TritonX100, 2% BSA to detect hyperphosphorylated tau. The following day, sections were washed three times in PBS and incubated with anti-mouse secondary antibody (Alexa 555-conjugated, Invitrogen, A21422, RRID: AB_141822) for 2 h at RT. Fluorescence-stained sections were washed three times for 10 min each in PBS, mounted on Superfrost plus slides (Thermoscientific, Waltham, MA) and air dried overnight.

To reveal beta-pleated structures, AT8-stained dried sections on mounted slides were circled with DakoPen (Merck, Z377821), and incubated for 25 min in 0.3% thioflavin-S (Thio-S, Sigma, T1892) in a 50:50 ethanol: water mixture. Staining was differentiated in a 50%/50% ethanol/water mixture for 10 min, and sections were rinsed twice for 1 min in water. The sections were then air dried and coverslipped with Fluoromount G mounting medium (ThermoFisher, 00-4958-02).

After drying for 24 h, sections were viewed on a Zeiss AxioImager1 microscope using an Alexa 488 detection filter for Thio-S and an Alexa555 detection filter for AT8 under a 20x objective. AT8- and Thio-S-positive neuronal profiles were manually quantified separately in the cortex and hippocampal CA1 using a cell counter, and the average number/section of AT8- and Thio-S-positive neuronal profiles was calculated for each mouse. Statistical comparisons were made using a two-tailed Student’s t-test after confirming the normality of the data, and correlation analysis was performed using Pearson’s R and GraphPad Prism software (v10.3.0) (RRID: SCR_002798).

### Single-Cell RNA sequencing

For single cell gene expression studies, fresh cortical tissue was dissociated with the Adult - Brain Dissociation kit (Miltenyi Biotec, #130-107-677), following the manufacturer’s protocol.

The dissociated cells were filtered through a 40 μm strainer, assessed for viability (> 80% required to proceed), and adjusted to a concentration of 120 cells/µl. Single-cell suspensions were then processed using a microfluidics-based approach following a modified Drop-seq protocol [11]. Briefly, cells were co-encapsulated with barcoded beads (ChemGenes Corp., 180 beads/µl in lysis buffer) in droplets generated using custom PDMS microfluidics devices fabricated by soft lithography with an aspect depth of 90 μm. Droplet generation was performed at flow rates of 2.5 ml/h and 11 ml/h for the cell/bead suspensions and carrier oil, respectively. After droplet breakage, captured mRNAs were reverse transcribed using Maxima H-RT in RT buffer (1× Maxima RT buffer, 4% Ficoll PM-400, 1 µM dNTPs, 1 U/ml RNase Inhibitor, 2.5 µM Template Switch Oligo), followed by exonuclease treatment and PCR amplification (4 cycles at 98 °C/65°C/72°C, 9 cycles at 98 °C/67°C/72°C). Libraries were purified using 0.6× AMPure XP beads, tagmented using Nextera XT, and sequenced on an Illumina NextSeq 500 with paired-end reads (20 bp for cell barcodes/UMIs and 60 bp for transcript sequences). Raw sequencing data was processed using the standard Drop-seq computational pipeline as detailed in previous work [11].

### Sequencing data Pre-Processing

The scRNA-seq data was processed in the R statistical programming environment (version 4.4.0, RRID: SCR_001905) using the dedicated software package *Seurat* (version 5.1.0, RRID: SCR_007322). The data underwent quality control filtering using established metrics to ensure robust downstream analysis. Specifically, we filtered out potential technical artifacts by removing outlier cells according to three criteria: (1) cells with fewer than 200 unique feature counts, which likely represent empty droplets; (2) cells with more than 7,000 unique feature counts, which may indicate cell doublets or multiplets; and (3) cells with greater than 5% mitochondrial gene content, which often signifies stressed, damaged, or apoptotic cells. These quality control thresholds were selected according to the distribution of these metrics across all samples and established best practices in single-cell analysis.

All samples passed these quality control assessments and were retained for subsequent analysis. Following filtration, the data were normalized and scaled using the *SCTransform* function in Seurat, which employs a regularized negative binomial regression model to normalize cellular gene expression values while controlling for technical confounders. We then identified the 2,500 most variable genes across the dataset using the *FindVariableFeatures* function, prioritizing genes likely to be most informative for distinguishing between different cell types and states. These selected features were scaled using the *ScaleData* function with default parameters to prepare the data for dimensional reduction and clustering. The final quality-controlled dataset comprised 28,190 cells and 17,681 genes, which formed the basis for all subsequent analyses.

### Bioinformatic analysis

#### Cell type identification and clustering

Before clustering the data for cell type annotation, dimensionality reduction was performed using principal component analysis (PCA) followed by Uniform Manifold Approximation and Projection (UMAP) for visualization [17]. The optimal number of principal components was determined through two complementary approaches: (1) the elbow method implemented in Seurat v5.1.0 *FindElbowPoint* function and (2) a quantitative cumulative variance analysis where we selected components until the percent change in variation between consecutive PCs fell below 0.1%. This approach identified 19 principal components as optimal for downstream analysis.

Cell clustering was performed using the shared nearest neighbor (SNN) graph-based clustering algorithm (Seurat v5.1.0 *FindClusters* function) [18] with resolution parameter 0.06, selected by maximizing the average silhouette width across multiple resolutions (0.001-1.0) using the cluster R package (v2.1.6). This optimization identified 9 distinct clusters as optimal, with an average silhouette width of 0.463. The *FindNeighbors* function was run with dims = 1:19 and default parameters. All visualizations were generated using *ggplot2* v3.5.1.

Cell type annotation was conducted using the ScType algorithm [19] with marker genes sourced from the Cell Marker database (RRID: SCR_018503) [20]. Cell identities were assigned based on cell type-specific expression patterns, using a scoring approach that weights markers by their specificity (marker sensitivity threshold = 0.5). Cluster identity was assigned to the highest-scoring cell type when the cumulative score exceeded 25% of the total cell count in that cluster. Finally, the validity of the determined cell type identities was further corroborated by comparing the most significant differentially expressed genes (DEGs) within each cluster against established markers from the database *PanglaoDB* (RRID: SCR_022580) [21].

#### Differential gene expression analysis

For our analysis of differential gene expression, we used a Poisson regression model, implemented in the *Seurat* v5.1.0 package [22]. This approach was applied to identify DEGs between genotypes with both sex-dependent patterns and shared patterns across the sexes. Specifically, we categorized DEGs into three primary classes according to their expression patterns across sexes and genotypes: (1) *Sex-neutral DEGs*: Genes exhibiting significant changes (FDR < 0.05, min. absolute log fold change: 0.5) between transgenic and wild-type animals in both sexes, with the same sign of the log fold change; (2) *Sex-specific DEGs*: Genes showing significant differential expression (FDR < 0.05, min. absolute log fold change: 0.5) in one sex exclusively, while showing no trend toward even nominal significance in the opposite sex (nominal P-value > 0.5; this conservative cut-off for non-significance was chosen to avoid spurious detection of sex-specificity due to stochastic variation of p-value estimates around commonly used significance thresholds); (3) *Sex-dimorphic DEGs*: Genes demonstrating significant changes in both sexes (FDR < 0.05, min. absolute log fold change: 0.5), but with opposing directions of change.

These three types of DEGs were assessed and compared in three different analyses: (1) cross-sectional examination of DEGs at 17 months of age, (2) comparison of these DEGs with those identified at 7 months of age, which have been discussed in more detail in a dedicated publication focused on early-stage changes [12], and (3) age-dependent assessment of expression changes between the two time points, contrasting transgenic and wildtype animals while accounting for sex-neutral, sex-specific, and sex-dimorphic effects. We build on our previous analysis of cross-sectional changes at 7 months of age [12] here to enable temporal comparisons.

Furthermore, we determined genes exhibiting sex differences in the magnitude, but not direction, of expression changes through an analysis of sex-genotype interactions. The resulting genes with more subtle sex-dependent expression patterns are presented as additional, lower-confidence candidates for sex-related DEGs in the Supplementary Materials, while our main discussion is centered on the three primary categories (sex-neutral, sex-specific, and sex-dimorphic DEGs).

To identify both cell type-specific gene expression changes and common changes, we performed independent analyses for specific cell types and across all cell types integrated (pseudobulk analysis). While we filtered out consistently low-expressed genes before analysis, some genes showed minimal expression in just one condition in either bulk or cell type-specific analyses. For these cases, the estimated log fold changes and p-values can reach extreme values. Although these results correctly identify differentially expressed genes and preserve their relative rankings, they may overestimate both the magnitude of change and statistical confidence. Our interpretation therefore emphasizes the broader patterns of differential expression and relative gene rankings rather than focusing on specific numerical estimates.

To contextualize our findings and validate them across species and models, we compared our results with human AD *post-mortem* brain data [9] and single-cell data from the Tg2576 mouse model [11]. Specifically, these single-time-point datasets from Tg2576 mice (24 weeks) and human AD *post-mortem samples* were compared against the THY-Tau22 data for both 7 months of age [12] and 17 months of age (collected for this study) to identify robust changes shared across models, time points and species. For this purpose, mouse and human gene symbols were standardized using the *HGNChelper* R package (version 0.8.1). Visualization of the gene overlap across different studies in Venn diagrams was performed using the *ggVennDiagram* R package (version 1.5.2) [23].

#### Gene set enrichment and pathway analysis

To explore the effect of sex-dependent transcriptomic alterations on cellular pathways, we applied an over-representation analysis using the *enrichGO* function from the R package *clusterProfiler* (version 4.12, RRID: SCR_016884) [24] and annotations from the Gene Ontology (RRID: SCR_010326) database. The Gene Ontology enrichment analysis was conducted using two different subsets: biological processes (BP) and molecular functions (MF). We sourced the mouse gene annotations from the *org.Mm.eg.db* package (version 3.19.1, RRID: SCR_002811). Enrichment significance was determined using an adjusted p-value cutoff of 0.05. Finally, we visualized the enrichment results using *enrichplot* (version 1.24.0) and *cowplot* (version 1.1.3, RRID: SCR_018081).

#### Gene regulatory network (GRN) analysis

To investigate coordinated changes in gene activity within regulatory sub-networks, we constructed gene regulatory networks (GRNs) using a dedicated network reconstruction algorithm [25] in combination with the GeneGo MetaCore™ knowledge database (RRID: SCR_008125). This database integrates curated, experimentally validated gene-gene interactions derived from the scientific literature. For this analysis, we relied only on the retrieved interactions with regulatory effects and defined directionality, focusing on categories such as “Regulation”, “Transcriptional Regulation”, “Influence on Expression”, “Binding”, and “co-Regulation of Transcription”. To perform the network analysis, we only used statistical significance (FDR < 0.05) as a filter without requiring a minimum effect size, as this approach can detect coordinated changes across multiple genes that might be missed when examining only large individual gene changes.

For genotype-specific GRN reconstruction, we employed a condition-specific algorithm [25] that refines the initial network based on the binary gene expression profiles (increased expression = 1, decreased expression = 0) of transgenic and wild-type phenotypes. This method uses a genetic algorithm-based optimization to exclude interactions from the initial knowledge-based network that do not align with the observed expression states. Additionally, for interactions in the MetaCore database lacking activation or inhibition annotations, the algorithm inferred these regulatory effects from the expression data and the network topology [25].

#### Network perturbation analysis

While the GRN analysis already highlights key regulatory genes that influence the expression of the DEGs, a more refined ranking of these regulators can be achieved by simulating the effects of modifying their activity. To this end, we performed an algorithmic network perturbation analysis [25], which evaluates the ability of candidate regulators (perturbagens) to counteract downstream gene expression changes. Regulators with higher perturbation scores have the potential to partially reverse pathological expression patterns in a significant subset of their target genes. Consequently, the top-ranked regulators from this analysis represent promising candidates for initial drug target discovery, laying the ground for further in-silico druggability assessments and subsequent experimental validation.

## Results

### Exploratory activity, cognitive impairment, and neuronal Tau inclusions in 17-month-old THY-Tau22 mice

**Fig. 2:**
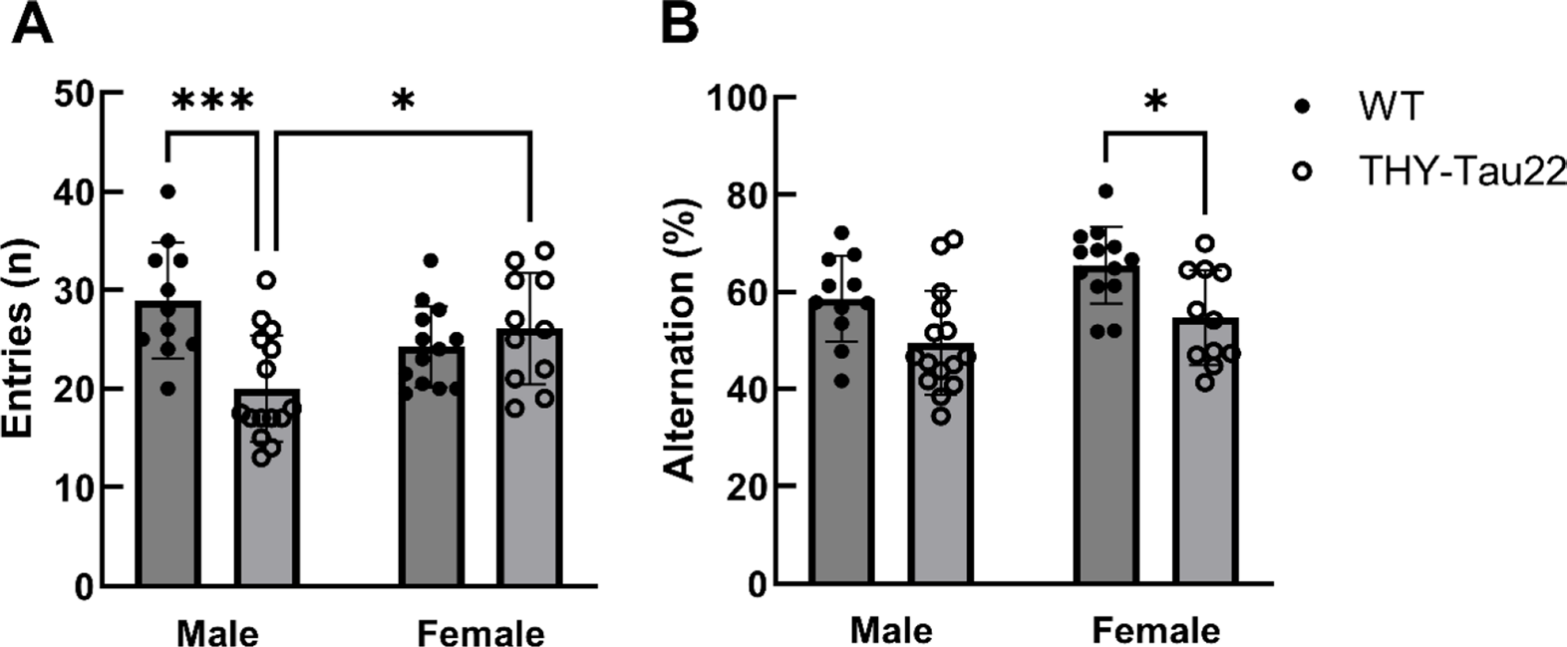
Sex-specific differences in exploratory activity and working memory in THY-Tau22 mice (Y-maze). (**A**) Number of arm entries in 17-month-old mice (*n* = 17 male THY-Tau22, *n* = 11 male WT, *n* = 11 female THY-Tau22, *n* = 13 female WT). (**B**) Percentage of alternations (*: *p* < 0.05, ***: *p* < 0.001)

To confirm that the THY-Tau22 mice of our colony had an AD-like phenotype at 17 months of age, we tested their cognition with the Y-maze, and tested for the presence of tau inclusions. Specifically, we first used the Y-maze to test exploratory activity and spatial working memory. By analyzing the number of entries into the arms of the Y-maze, a measure of exploratory activity, we found a genotype effect (F(1,46) = 5.46; *p* = 0.02), no significant sex effect, but a significant interaction between the two (F(1,46) = 12,82; *p* = 0008). Post hoc analysis indicated that wild-type males explored a similar number of arms as their female counterparts. However, THY-Tau22 males showed a significantly less exploratory activity compared to their sex-matched wild-type littermates (WT: entries = 28.95; THY-Tau22: entries = 20.03; *p* = 0.0004) and to THY-Tau22 females (male entries = 20.03; female entries = 26.09; *p* = 0.023) (Fig. 2A).

Importantly, when analyzing the percentage of alternations, which measures cognitive performance, we found effects of both sex (F(1,46) = 5.051; *p* = 0.0295) and genotype (F(1,46) = 13.67; *p* = 0.0006). Post hoc analysis suggested that male and female wildtype littermates had similar percentages of alternations. However, while the percentage of alternations in THY-Tau22 males compared to their sex-matched wild-type controls did not reach significance (*p* = 0.077), this decrease was significant in transgenic THY-Tau22 females compared to their wild-type controls (*p* = 0.029) (Fig. 2B).

**Fig. 3:**
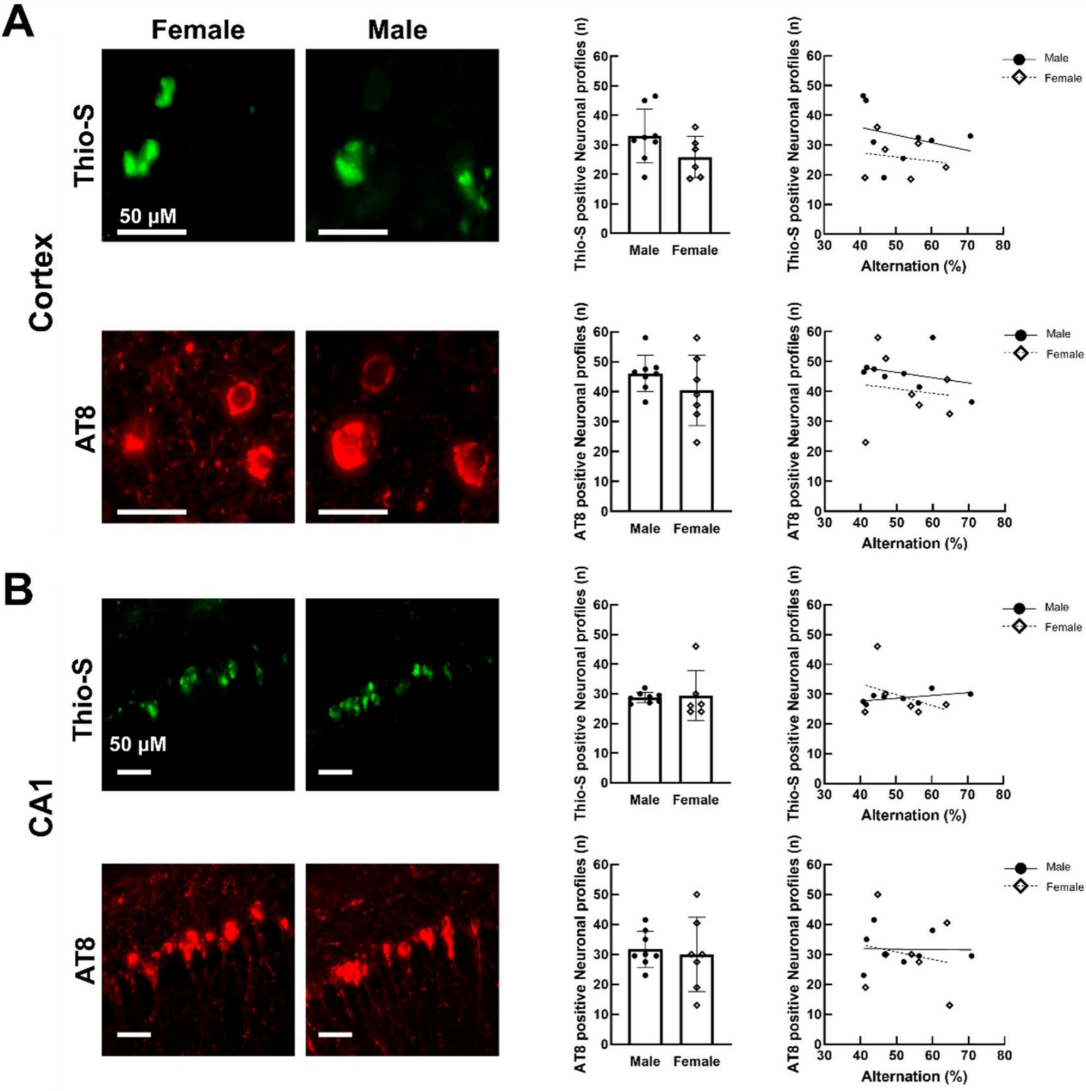
Tau pathology markers and correlation with cognitive function in THY-Tau22 mice at 17 months (*n* = 7 mice per sex). (**A**) Cortical region showing Thioflavin-S (green) and AT8 (red) staining (left), quantification (middle column), and correlation with alternation behavior (right). Scale bar: 50 µM. (**B**) Corresponding analyses for hippocampal CA1 region. Data shown as mean ± standard error of the mean (SEM) with individual data points in correlation plots

We quantified intraneuronal tau inclusions by counting the number of Thio-S-positive and AT8-positive neuronal profiles in the cortex and CA1 region of the hippocampus in THY-Tau22 mice of both sexes. We found no signal in these two brain regions in wild-type controls of either sex (not shown). Furthermore, we found no significant difference between the sexes in either the number of Thio-S-positive or that of AT8-positive neuronal profiles in the cortex (Fig. 3A) or hippocampus of THY-Tau22 mice (Fig. 3B). The number of Thio-S-positive or AT8-positive neuronal profiles in the cortex and the hippocampus did not correlate significantly with the percentage of alternation in either sex (Fig. 3, panels A and B; last column).

### Characterization of cell types and marker genes

**Fig. 4:**
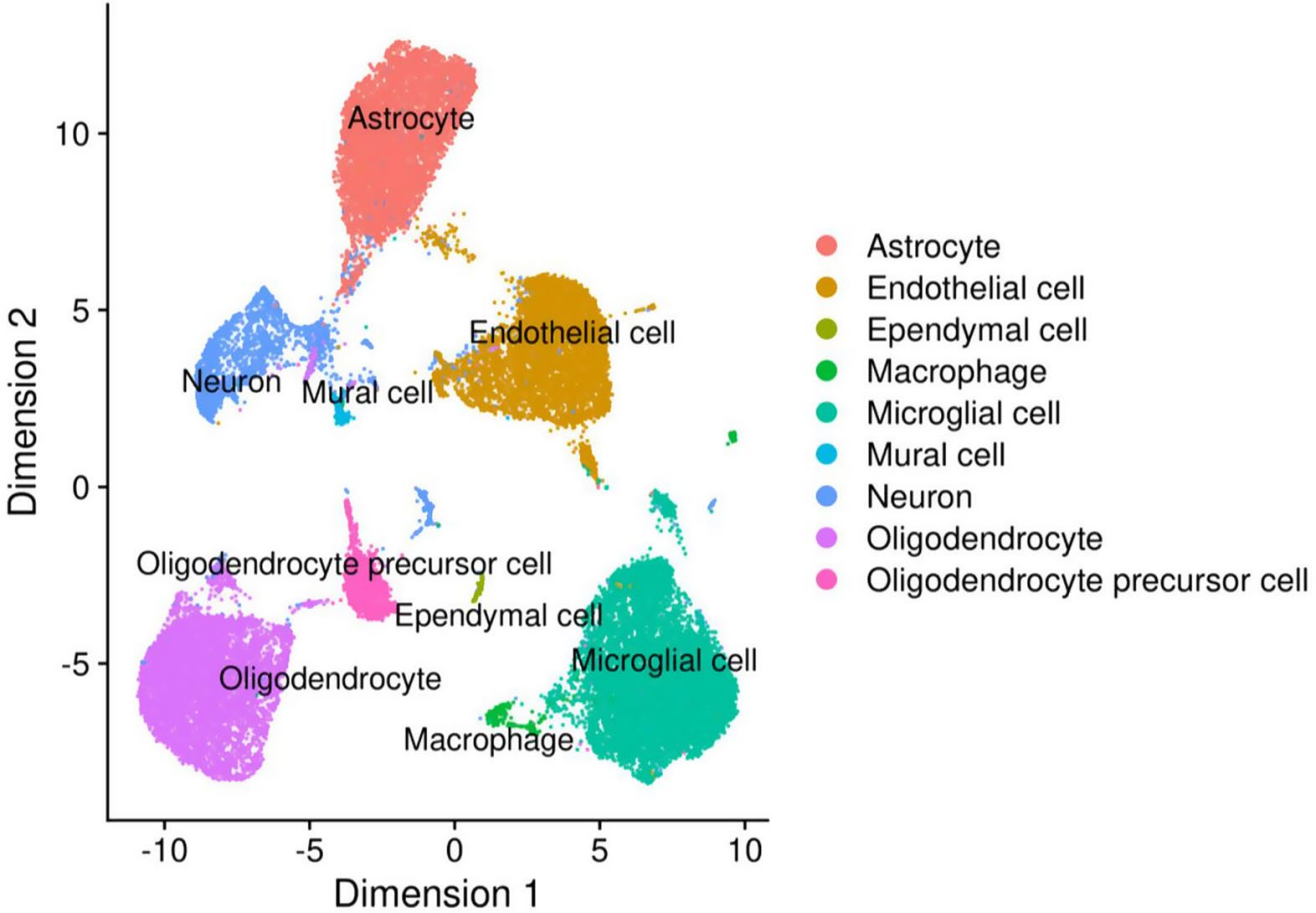
UMAP visualization of the scRNA-seq data clustering at 17 months. Annotation of cell clusters representing distinct cell types was performed using SCType on pooled data from all experimental groups (*n* = 16 mice total). The corresponding figure for 7-month data is shown in Suppl. Figure 1

Our scRNA-seq analysis identified 9 different cell types in the mouse cortex samples for the 17 months time point (Suppl. Table ST1). The most abundant cell types were microglia, oligodendrocytes, astrocytes, and endothelial cells, each with over 5,000 cells profiled for both time points. Neurons and oligodendrocyte precursor cells (OPCs) were also well represented (over 1k cells for the 17 months time point, compared to ~ 0.8k cells for the 7 months time point). Smaller populations of macrophages, mural cells, and ependymal cells were detected, although the low cell counts for these types (less than 600 cells each) limited our ability to robustly characterize subpopulations. Given these abundance profiles, we focused our downstream analyses of transcriptomic changes on the major glial and vascular cell types. The cell type clusters were visualized using the UMAP dimensionality reduction, with annotated results shown in Fig. 4 (17 month time point; Suppl. Fig. S1 shows the corresponding results for the 7-month time point, adapted from our previous study [12]). A heatmap visualizing cell-type-specific marker gene expression across identified cell type clusters is provided in Suppl. Fig. S2.

### Sex-dependent and sex-neutral gene-level transcriptomic changes

#### Global transcriptomic alterations across all cell types (THY-Tau22, 17 months of age)

In the global gene-level analysis of differential expression of THY-Tau22 mice vs. wild-type littermates across all cell types combined (also known as pseudobulk analysis) at 17 months of age, we identified 15 male-specific DEGs, 1 female-specific DEG, 21 sex-dimorphic DEGs (showing changes in both sexes but with opposing directions), and 10 sex-neutral DEGs. A detailed list of these DEGs is provided in Suppl. Table ST2.

Male mice showed a stronger transcriptional response to tau pathology, as evidenced by the higher number of male-specific DEGs. Furthermore, several sex-dimorphic DEGs (34) were observed, where genes changed in opposite directions between males and females.

**Fig. 5:**
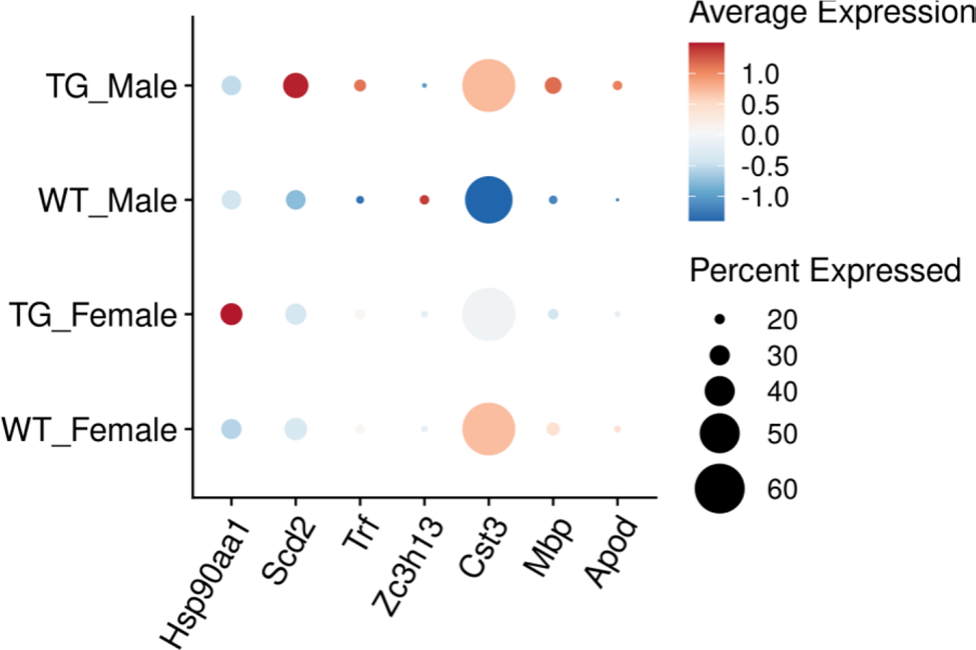
Sex-dependent gene expression patterns in 17-months old mice. Dot plot showing expression of key differentially expressed genes across the four experimental groups. Dot size indicates percentage of cells expressing each gene; color intensity represents average expression level (red = high, blue = low). Selected genes include female-specific (*Hsp90aa1*), male-specific (*Scd2*, *Trf*, *Zc3h13*), and sex-dimorphic (*Cst3*, *Mbp*, *Apod*) DEGs

Below, we briefly describe the top significant DEGs with known functional annotations in each category (male-specific, female-specific, sex-dimorphic, and sex-neutral DEGs). A summary of these genes and their alteration statistics in males and females is provided in Table 1, and a dot plot visualization of the sex-dependent changes is shown in Fig. 5.

**Table 1:** Most significant sex-dependent differentially expressed genes between THY-Tau22 and wild-type mice at 17 months of age

Type	Gene Symbol	Direction (females)	Direction (males)	FDR Female	FDR Male
Sex-neutral	*Fth1*	↑	↑	1.97E-09	<2.50E-274
Sex-neutral	*Malat1*	↓	↓	1.96E-21	<2.50E-274
Sex-neutral	*Plp1*	↓	↓	5.18E-66	<2.50E-274
Male-specific	*Scd2*	**-**	↑	1	<2.50E-274
Male-specific	*Trf*	**-**	↑	1	1.09E-19
Male-specific	*Zc3h13*	**-**	↑	1	8.00E-16
Female-specific	*Hsp90aa1*	↑	**-**	0.0053	1
Sex-dimorphic	*Cst3*	↓	↑	1.37E-44	<2.50E-274
Sex-dimorphic	*Mbp*	↓	↑	2.42E-12	<2.50E-274
Sex-dimorphic	*Apod*	↓	↑	0.00015	9.63E-124

All genes shown exhibited significant expression changes (FDR < 0.05) with absolute log2 fold-change ≥ 0.5 in the sex(es) where they showed significance. Direction refers to expression changes in THY-Tau22 mice compared to wild-type controls (↑: increased, ↓: decreased, -: no significant change). FDR values are adjusted p-values correcting for multiple testing. Sex-neutral genes showed consistent changes in both sexes. Sex-specific genes showed significant changes in only one sex, with no trend toward significance in the other (*P* > 0.5). Sex-dimorphic genes showed opposing directions of change between sexes, with a minimum difference in log2 fold-change of 0.5

*Male-Specific DEGs*:

1. *Scd2* (FDR = 2.54E-20, logFC = 1.49): Stearoyl-CoA desaturase 2, involved in fatty acid metabolism. Its male-specific increased expression highlights sex-specific alterations in lipid metabolism and membrane fluidity [26].
2. *Trf* (FDR = 1.09E-19, logFC = 8.98): Transferrin, essential for iron transport and homeostasis. Its strong male-specific overexpression indicates pronounced sex-specific differences in iron metabolism and transport [27].
3. *Zc3h13* (FDR = 8.00E-16, logFC=-0.83): Zinc finger CCCH-type containing protein 13, involved in RNA methylation and processing, pointing to decreased expression of RNA modification pathways in males [28].

*Female-Specific DEGs* (only one identified):

- *Hsp90aa1* (FDR = 0.005, logFC = 0.65): Heat shock protein significantly over-expressed in females but non-significantly underexpressed in males, indicating sex-specific differences in protein folding and stress response pathways and matching with unbalanced expression of human HSP90AA1 in the entorhinal cortex previously reported for Aβ pathology [29].

*Sex-Dimorphic DEGs*:

1. *Cst3* (up in males, FDR < 2.50E-274; down in females, FDR = 1.37E-44): Cystatin C, an inhibitor of cysteine proteases and major AD risk factor [30]. Cystatin C has been shown to bind to Aβ and inhibit Aβ oligomerization and amyloid fibril formation [30].
2. *Mbp* (up in males, FDR = 1.67E-135; down in females, FDR = 2.42E-12): Myelin basic protein, involved in myelin maintenance and repair, showing strong opposing gene expression changes between sexes. This protein has been reported to associate with amyloid plaques in the cortex of AD brains [31].
3. *Apod* (up in males, FDR = 9.63E-124; down in females, FDR = 1.47E-4): Apolipoprotein D, involved in lipid metabolism and neuroprotection, showing highly significant divergent expression changes.

*Sex-Neutral DEGs*:

1. *Fth1* (up in both sexes, male FDR < 1.67E-135, female FDR = 1.97E-09): Ferritin heavy chain, a regulator of iron homeostasis.
2. *Malat1* (down in both sexes, male FDR < 1.67E-135, female FDR = 1.96E-21): Long non-coding RNA involved in gene expression regulation and splicing.
3. *Plp1* (down in both sexes, male FDR = 2.50E-274, female FDR = 5.18E-66): Proteolipid protein 1, essential for myelin structure.

Overall, differential expression analysis identified several key patterns associated with tauopathy. We observe strong sex-dependent patterns in protein homeostasis and processing (*Cst3*), myelin-related gene expression (*Mbp*), and lipid metabolism (*Apod*), where males and females show opposing responses. These sex-dimorphic changes are complemented by male-specific alterations in lipid metabolism (*Scd2*), iron transport (*Trf*), and RNA processing (*Zc3h13*), as well as female-specific changes in stress response pathways (*Hsp90aa1*). However, some core disease processes are shared between sexes in the bulk level analysis, as shown by similar changes in both males and females for genes involved in iron homeostasis (*Fth1*), transcriptional regulation (*Malat1*), and myelin structure (*Plp1*).

#### Cell type-specific transcriptomic alterations

**Fig. 6:**
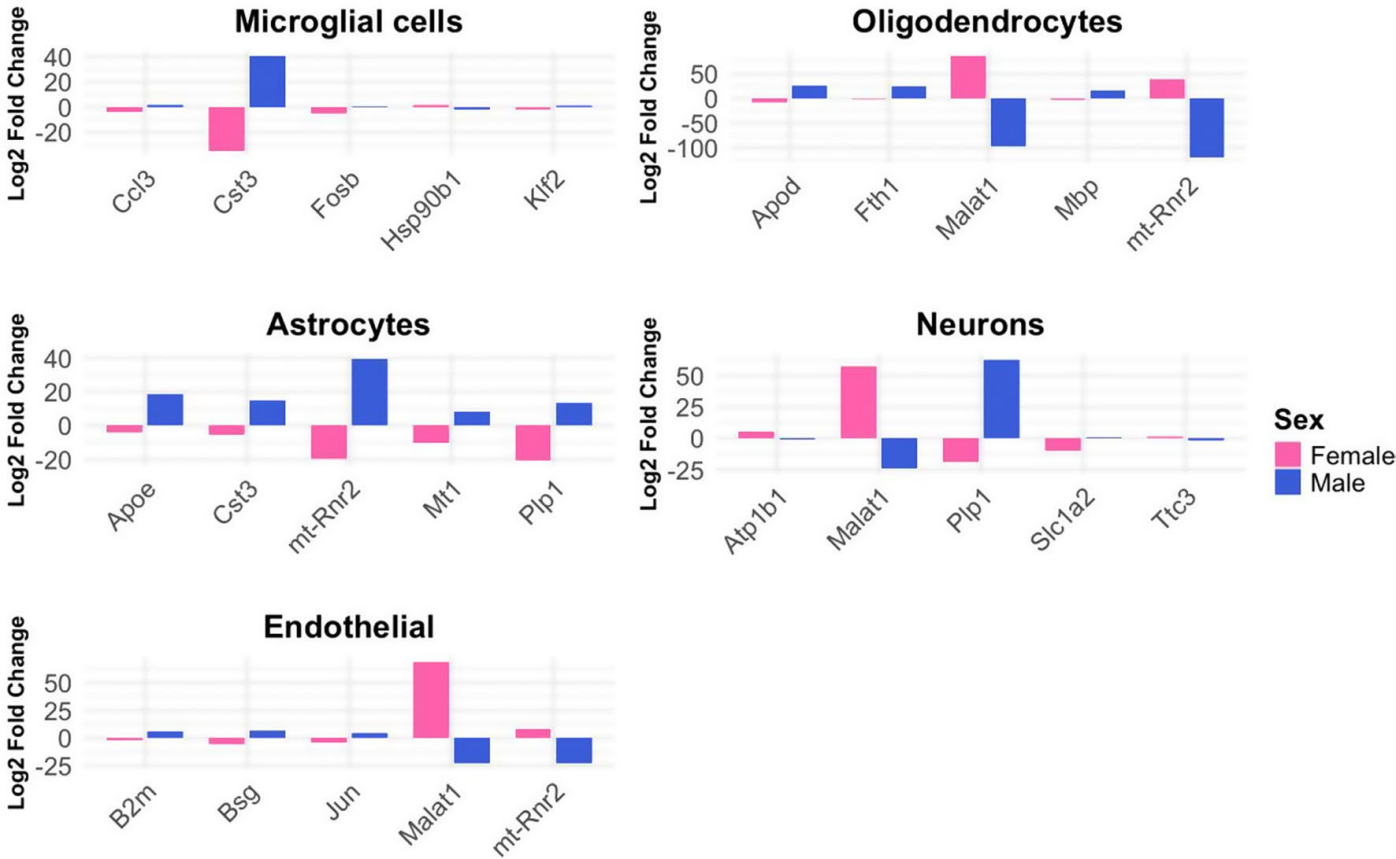
Top 5 sex-dimorphic differentially expressed genes across major brain cell types in 17-month-old THY-Tau22 mice. Bar plots show log2 fold changes between THY-Tau22 and wild-type mice. Blue bars = male changes; pink bars = female changes. All genes shown had significant differential expression (FDR < 0.05) with opposing directions of change between sexes

The cell type-specific analyses revealed varying patterns of differential expression across cell populations. Due to the limited number of cells in smaller clusters, which reduce the statistical robustness of differential expression analysis, we focused our investigation on the five major cell types with sufficient representation: microglia, astrocytes, neurons, oligodendrocytes, and endothelial cells. Bar plot and volcano plot representations of the top five most significant sex-dimorphic and sex-specific DEGs with known gene symbols for these major cell types are presented in Fig. 6 and Suppl. Fig. S3, respectively. An overview of the DEG counts per category (female-specific, male-specific, sex-dimorphic, or sex-neutral) for five main brain cell types (microglia, astrocytes, neurons, oligodendrocytes, and endothelial cells) is shown in Suppl. Fig. S4.

Microglial cells showed the highest number of DEGs, with 14 male-specific DEGs, 1 female-specific DEGs, 6 sex-dimorphic DEGs, and 9 sex-neutral DEGs. Other cell types with notable changes included oligodendrocytes (11 male-specific, 7 sex-dimorphic, 3 sex-neutral), astrocytes (9 male-specific, 1 female-specific, 12 sex-dimorphic, 2 sex-neutral) and neurons (7 male-specific, 11 sex-dimorphic, 4 sex-neutral). The complete breakdown of all DEGs for all cell types is shown in Suppl. Table ST3.

Overall, our analysis revealed a pronounced sex-specific pattern in transcriptional responses to tau pathology across all major cell types at 17 months. Male cells exhibited both a broader and stronger response, with male-specific significant expression changes consistently outnumbering female-specific alterations.

Considering the functional annotations of the identified DEGs, our single-cell transcriptomic analysis also highlighted distinct patterns across the major brain cell types for both sex-neutral and sex-dependent changes (see the summary for top-ranked genes in Table 2).

In microglia, we found strong male-specific upregulation of inflammatory and immune response genes (*Malat1*: logFC = 24.90, FDR = 1.82E-266; *H2-D1*: logFC = 0.88, FDR = 2.61E-44; *Tyrobp*: logFC = 5.14, FDR = 3.84E-36), while females showed specific upregulation of stress response in line with the pseudobulk analysis results (*Hsp90ab1*: logFC = 2.42, FDR = 8.07E-4). Similarly, astrocytes exhibited male-specific changes in protein homeostasis and barrier function (*Clu*: logFC = 6.16, FDR = 1.42E-14; *Cldn10*: logFC = 0.79, FDR = 2.44E-14; *Cpe*: logFC = 2.39, FDR = 6.82E-14) and female-specific alterations in RNA processing (*Pnisr*: logFC = 1.88, FDR = 1.35E-4).

Neurons showed male-specific changes in particular in mitochondrial and matrix proteins (*mt-Cytb*: logFC = 15.80, FDR = 8.75E-29; *Sparcl1*: logFC = 7.19, FDR = 1.58E-26; *Snhg11*: logFC = 2.50, FDR = 2.37E-10). In oligodendrocytes, we observed strong male-specific changes in carbonic anhydrase and mitochondrial complex I gene members (*Car2*: logFC = 7.15, FDR = 4.30E-90; *mt-Nd1*: logFC = 14.84, FDR = 1.57E-38; *mt-Nd2*: logFC = 10.84, FDR = 1.11E-31), while endothelial cells showed male-specific changes in transcriptional regulation (*Id3*: logFC = 1.07, FDR = 1.82E-15) and female-specific changes in chemokine signaling (*Cxcl12*: logFC=-1.34, FDR = 1.10E-3).

Strong sex-dimorphic expression patterns were observed across all cell types, including *Cst3* in microglia, *mt-Rnr2* in astrocytes, *Plp1* in neurons (all up in males/down in females, see Suppl. Tab. ST3), *Malat1* in oligodendrocytes, and *mt-Rnr2* in endothelial cells (all down in males/up in females, see Suppl. Tab. ST3). Finally, we also identified significant sex-neutral transgene-associated patterns, such as *Apoe* overexpression in microglia, *Malat1* underexpression in astrocytes, *Meg3* underexpression in neurons, and *mt-Rnr1* underexpression in oligodendrocytes and endothelial cells (see Suppl. Tab. ST3).

To identify additional sex differences affecting only the magnitude of expression changes (rather than the direction or presence/absence of significant changes), we also performed a statistical test for sex-genotype interactions using the software *edgeR*. This analysis identified numerous significant interactions (Suppl. Tab. ST4), including many genes that were also detected in our primary analysis using Seurat. For example, *Malat1* showed strong interaction effects across multiple cell types (e.g., with an adjusted p-value < 2.4E-31 in microglia), confirming its sex-dependent dysregulation. The interaction analysis also revealed additional candidate genes that display more subtle sex differences in only the magnitude of expression changes between males and females. Examples include *mt-Rnr1* in astrocytes (interaction adj. p-value = 1.1E-28) and *Aldoc* in astrocytes (interaction adj. p-value = 1.7E-20). While these additional results represent lower-confidence candidates for sex-dependent regulation compared to genes showing opposing significant changes or sex-specific effects, they may still contribute to the overall sex-dependent manifestation of tau pathology.

Overall, these cell type-specific analyses reveal pronounced sex differences in the response to tau pathology across all major brain cell types, with males showing stronger inflammatory and metabolic alterations (particularly in microglia and oligodendrocytes), whereas females exhibit changes in stress response and RNA processing. While certain pathways show robust sex-specific or sex-dimorphic regulation (such as inflammatory responses in microglia and myelin-related genes in oligodendrocytes), other key AD-related pathways (including *Apoe* signaling and mitochondrial pathways) show consistent dysregulation across both sexes depending on the cell type. This suggests that tau pathology affects both sex-dependent and sex-independent molecular mechanisms in a partly cell type-specific manner.

**Table 2:** Cell type-specific sex-dependent gene expression changes across major brain cell types in THY-Tau22 mice at 17 months of age

Cell Type	Sex-Specific Changes	Sex-Dimorphic Changes
**Microglia**	Malat1 (↑♂) H2-D1 (↑♂) Tyrobp (↑♂) Hsp90ab1 (↑♀)	Cst3 (↑♂/↓♀) Ccl3 (↑♂/↓♀) Hsp90b1 (↓♂/↑♀)
**Astrocytes**	Clu (↑♂) Cldn10 (↑♂) Cpe (↑♂) Pnisr (↑♀)	mt-Rnr2 (↑♂/↓♀) Apoe (↑♂/↓♀) Mt1 (↑♂/↓♀)
**Neurons**	mt-Cytb (↑♂) Sparcl1 (↑♂) Snhg11 (↑♂) Strbp (↓♂)	Plp1 (↑♂/↓♀) Atp1b1 (↓♂/↑♀) mt-Rnr2 (↓♂/↑♀)
**Oligodendrocytes**	Car2 (↑♂) mt-Nd1 (↑♂) mt-Nd2 (↑♂)	Malat1 (↓♂/↑♀) Fth1 (↑♂/↓♀) Mbp (↑♂/↓♀)
**Endothelial cells**	Id3 (↑♂) Cxcl12 (↓♀) Eif3a (↓♂)	mt-Rnr2 (↓♂/↑♀) B2m (↑♂/↓♀) Malat1 (↓♂/↑♀)

Summary of the most significant differentially expressed genes showing either sex-specific or sex-dimorphic patterns across five major brain cell types. Sex-specific changes indicate significant alterations (FDR < 0.05, absolute log2 fold-change ≥ 0.5) in one sex only (♂: male, ♀: female) with direction shown by arrows (↑: increased, ↓: decreased). Sex-dimorphic changes indicate significant opposing changes between sexes. Notable patterns include widespread male-specific upregulation across cell types, recurrent dysregulation of *Malat1* across multiple cell populations, and cell type-specific regulation of genes involved in inflammation (microglia), protein homeostasis (astrocytes), mitochondrial function (neurons), myelination (oligodendrocytes), and vascular function (endothelial cells). All changes compare THY-Tau22 mice to wild-type controls

### Shared gene-level alterations across mouse models, ages, and human AD

We compared the differential expression analysis results for THY-Tau22 mice at 17 months of age against an earlier time point for the same model, a distinct mouse model of Aβ pathology, and cortical gene expression data in human AD. Specifically, we computed overlaps between the following gene sets: (1) DEGs for THY-Tau22 vs. wildtype mice at 17 months of age, (1) DEGs for THY-Tau22 vs. wildtype at 7 months of age, (2) DEGs for the Tg2576 mouse model of Aβ pathology vs. wildtype (24-weeks old) [11], and (3) DEGs in *post mortem* human cortical tissue from AD patients vs. controls [9]. The analysis focused on the four major cell types consistently present across all datasets: neurons, astrocytes, microglial cells, and oligodendrocytes.

**Fig. 7:**
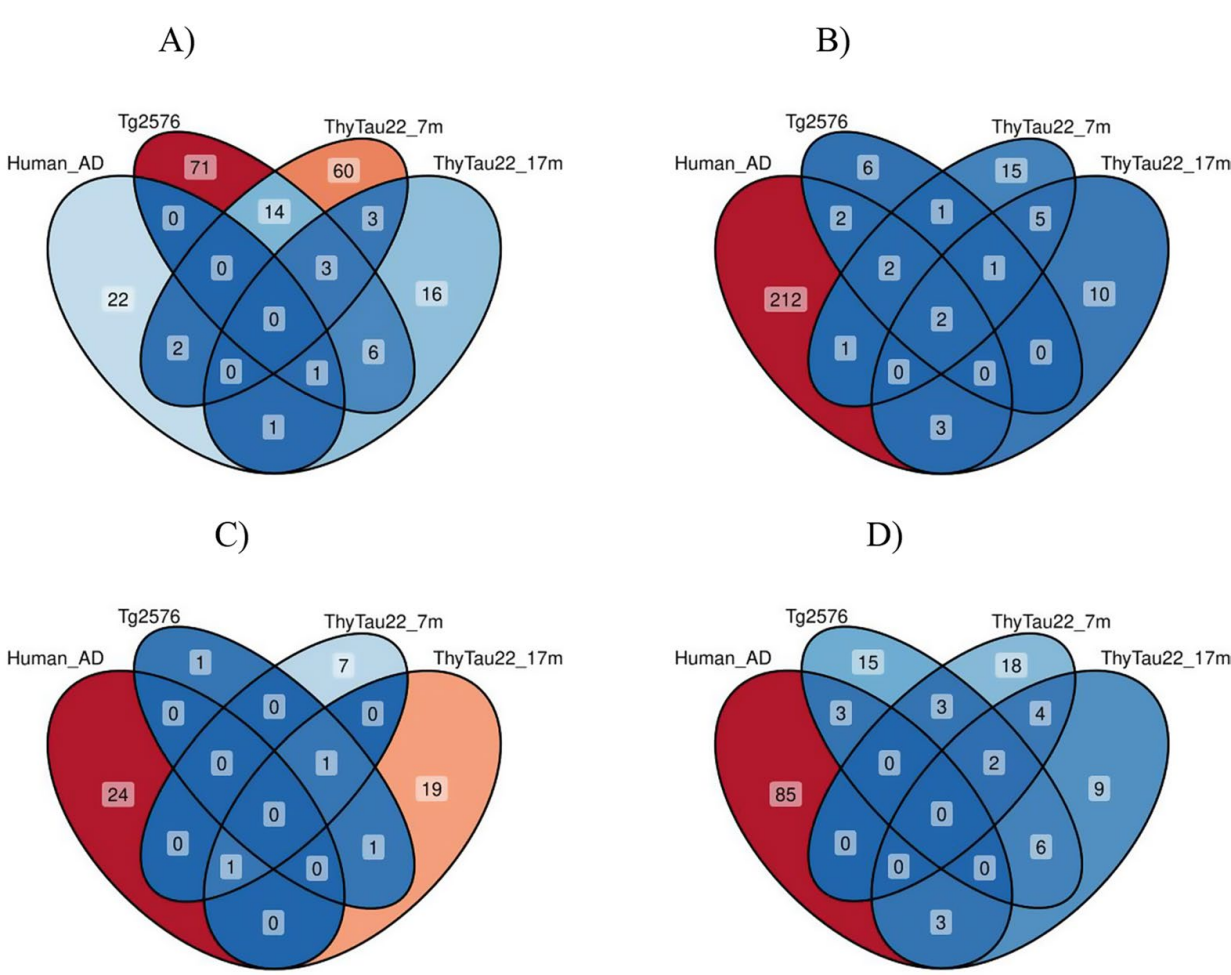
Cell type-specific overlaps of differentially expressed genes across mouse models and human AD data. Venn diagrams showing intersections of DEGs in human AD cortical tissue, Tg2576 model, and THY-Tau22 mice at 7 and 17 months in (**A**) microglial cells, (**B**) oligodendrocytes, (**C**) neurons, and (**D**) astrocytes. Numbers indicate DEG counts in each intersection set

Computing the intersections of DEGs for each of these cell types revealed limited overlap across the datasets (Fig. 7). No genes showed significant changes across all four datasets in microglia, neurons, or astrocytes. The overlap between the 7- and 17-month THY-Tau22 data was small across all cell types, with 2–8 shared DEGs between these time points depending on the cell type. In oligodendrocytes, only two genes (*Malat1* and *Mbp*) were differentially expressed across all datasets (Fig. 7b). *Malat1*, a long non-coding RNA, displayed an overall dominant pattern of male-specific underexpression in oligodendrocytes, though the sex-dependencies varied across the datasets. While it showed male-specific underexpression in human AD oligodendrocytes and sex-dimorphic expression in THY-Tau22 mice at 17 months of age (down in males, and up in females), it appeared as sex-neutral, underexpressed in oligodendrocytes in 7 months THY-Tau22 and Tg2576 models. Similarly, the myelin basic protein encoding gene *Mbp* showed dominant male-specific changes in oligodendrocytes, with consistent male-specific underexpression in the Tg2576 mouse model and in human AD; however, different patterns were observed in the ThyTau22 model for this gene, with a relative female-specific overexpression at the 7 months time point and significant sex-dimorphic changes at the 17 months time point (overexpression in males, and underexpression in females).

*Hsp90ab1*, a member of the heat shock protein 90 family with chaperone activity, displayed significant changes in microglia with varying patterns across three datasets: female-specific in 17 months THY-Tau22 (logFC = 2.5, FDR = 8.10E-04), male-specific in human AD data (logFC = 0.3, FDR = 8.25E-05), and sex-neutral in the Tg2576 model (logFC_m_= -0.22, FDR_m_=2.47E-07, logFC_f_= -0.16, FDR_f_=4.80E-03; Fig. 7a). The only other gene with significant alterations in the same cell type across a minimum of three datasets was *Atp1b1*, encoding a subunit of a sodium/potassium-transporting ATPase. This gene displays female–specific changes in human AD (logFC= -0.37, FDR = 9.95E-03) and THY-Tau22 mice (logFC= -0.51, FDR = 2.70E-06), and sex-dimorphic changes in the 17 months THY-Tau22 AD model (logFC_m_= -0.76, FDR_m_=6.35E-06, logFC_f_=5.31, FDR_f_=9.78E-22; Fig. 7c).

Additional significant patterns emerged when comparing smaller subsets of these datasets (see the Venn diagram in Fig. 7, detailed summary statistics in Suppl. Tab. ST18, and the overview of intersection set sizes in Suppl. Fig. S5). The well-established AD risk factor gene *Apoe* displayed sex-neutral microglial over-expression in both the 7 month and 17 months THY-Tau22 datasets. Finally, *Malat1*’s alteration patterns also extended beyond oligodendrocytes, e.g., showing consistent underexpression in astrocytes and OPCs as well, suggesting its broad involvement in multiple cell types relevant to AD pathology.

### Sex-dependent pathway alterations in single cell types

We investigated cell type-specific pathway alterations by performing separate gene set enrichment analyses for Gene Ontology (GO) terms within each major cell population (a global, pseudobulk analysis of pathway alterations across all cell types is presented in the Suppl. Materials, see also Suppl. Figures S6-S9 and Suppl. Tables ST6-ST7). Analyses were performed separately for male-specific and sex-dimorphic DEGs for each cell type (for female-specific DEGs, the number of globally significant DEGs was insufficient for enrichment analysis). Below, we report the results for the largest cell populations for the cross-sectional analysis of THY-Tau22 mice vs. wild-type mice at 17 months of age.

#### Sex-dependent pathway alterations (THY-Tau22 mice, 17 months of age)

##### Microglial cells

Sex-dimorphic DEGs (6 genes) showed enrichment in synaptic and inflammatory response pathways (Suppl. Table ST8). Among male-specific DEGs in microglia (14 genes), significantly enriched biological processes included the regulation of neuron apoptosis, amyloid-beta formation, and regulation of myeloid cell differentiation (adjusted *p* < 0.05, see the dot plot visualization in Suppl. Fig. S10-S13). Molecular functions showed strong enrichment for tau protein binding, ubiquitin protein ligase binding, and peptide binding.

##### Oligodendrocytes

Male-specific DEGs in oligodendrocytes (11 genes) showed significant enrichment in myelination, chemokine production, and regulation of amyloid fibril formation pathways (see the dot plot visualization in Suppl. Fig. S14-S17). Molecular functions were enriched for structural constituents of myelin sheath and ferric iron binding. Sex-dimorphic DEGs (7 genes) showed enrichment in lipid metabolism and membrane organization pathways (Suppl. Table ST9).

##### Astrocytes

Male-specific DEGs in astrocytes (9 genes) showed significant enrichment in pathways related to negative regulation of amyloid beta formation, central nervous system (CNS) myelination, and axon ensheathment in CNS. Molecular functions included tau protein binding, amyloid protein binding, and functions related to myelin sheath structural components. While the single female-specific DEG (*Pnisr*, encoding a serine and arginine rich splicing regulatory protein) was insufficient for pathway analysis, it has previously been implicated in dysfunctional lactate metabolism in human AD [32]. Sex-dimorphic DEGs (12 genes) showed enrichment in cellular stress response pathways (Suppl. Fig. S18-S21 and Suppl. Table ST10).

##### Neurons

In neurons, sex-dimorphic DEGs (11 genes) were strongly enriched in transmembrane transport pathways and sodium ion homeostasis, suggesting sex-dependent alterations in neuronal communication (Suppl. Table ST11, Suppl. Fig. S22-S25). Male-specific DEGs (7 genes) were enriched in symporter activity and glutamate binding related molecular functions.

### Gene regulatory network analysis of sex-dependent changes

To characterize the regulatory mechanisms involved in sex-dependent gene expression changes in THY-Tau22 mice, we performed differential gene regulatory network (GRN) analyses (see Methods). Beyond detecting potential disease-relevant regulatory mechanisms, this enabled us to determine key regulatory genes involved in controlling sex-dependent molecular disease phenotypes. The differential GRN inference approach was applied separately to the male-specific and sex-dimorphic DEGs in order to reconstruct phenotype-specific networks for the cross-sectional analysis of THY-Tau22 mice vs. wild-type mice at 17 months of age. Because the number of DEGs was only sufficient to build GRNs for the global analysis integrating all cell types, and only for male-specific and sex-dimorphic DEGs, we only report global rather than cell type-specific GRN analyses for these sex-dependent DEGs.

#### Gene regulatory networks (THY-Tau22, 17 months of age)

##### Male-specific differential GRNs

**Fig. 8:**
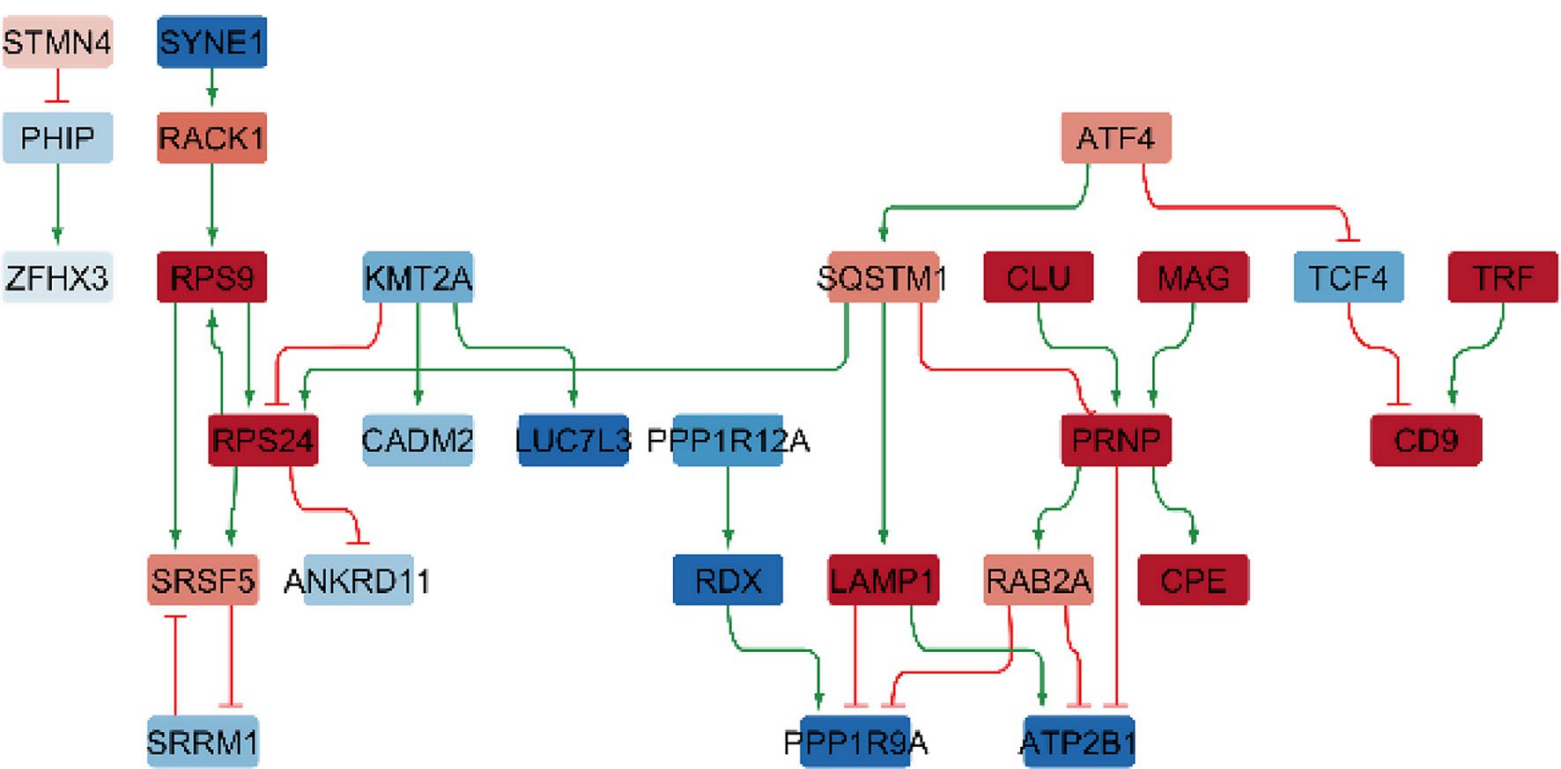
Gene regulatory network of male-specific differentially expressed genes in 17-month-old THY-Tau22 mice (global analysis across cell types). Nodes represent genes (red = increased expression, blue = decreased expression); edges show regulatory interactions (green arrows = activation, red lines = inhibition). The network comprises 28 nodes and 32 interactions (20 activations, 12 inhibitions). Key regulatory genes appear in the upper part with downstream targets below

For male-specific DEGs, two GRNs were built, reflecting the regulatory sub-networks controlling these genes in transgenic mice (see Fig. 8) and wild-type controls respectively. The THY-Tau22 sub-network involves 28 gene nodes and 32 interactions, while the WT network includes 32 genes and 35 interactions. Among the upstream transcription factors in this GRN, the known Alzheimer risk factor gene clusterin (*Clu*) [33] was identified as a regulator gene that showed significant male-specific increased expression and involvement in regulating multiple downstream genes.

##### Sex-dimorphic differential GRNs

**Fig. 9:**
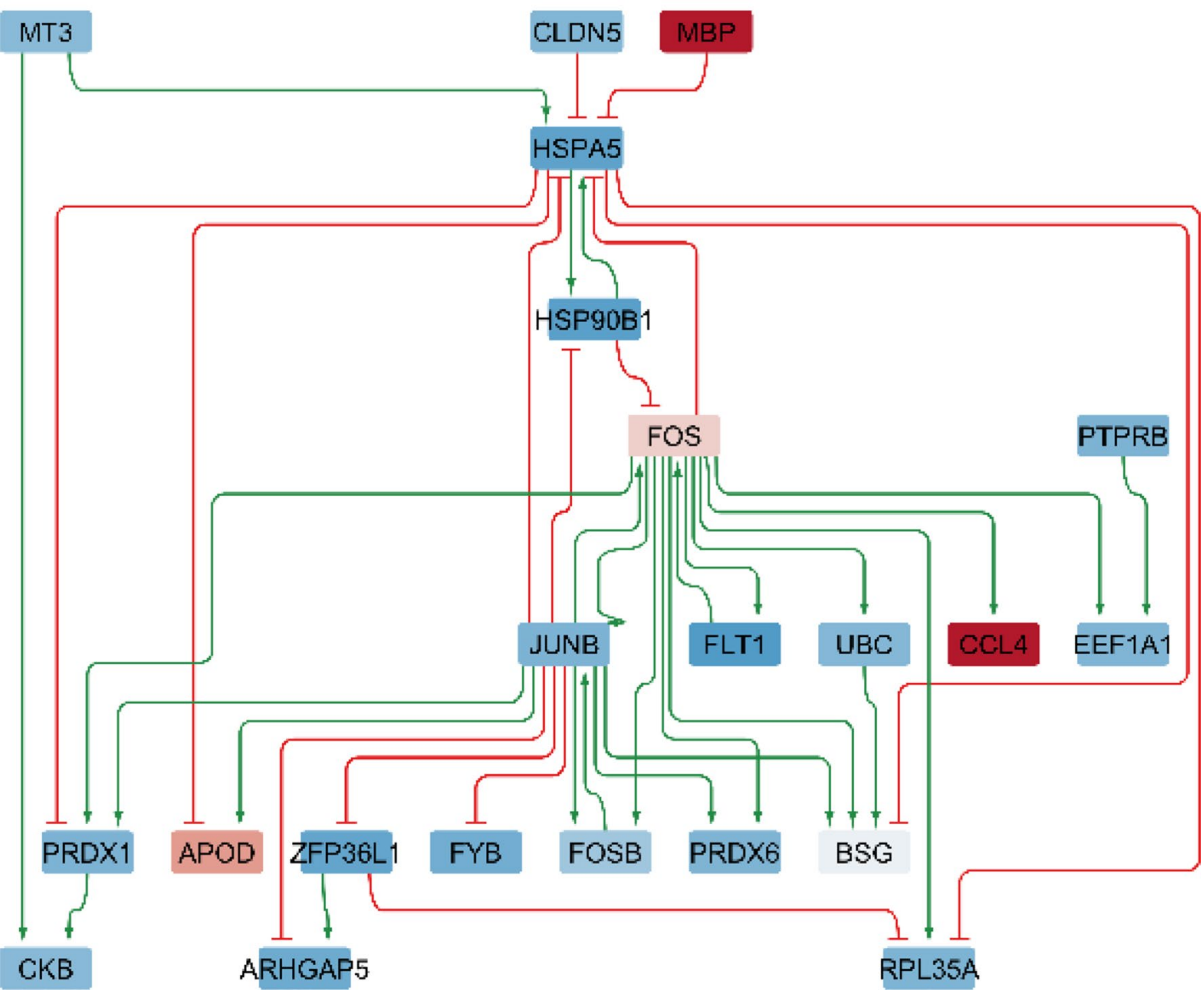
Gene regulatory network of sex-dimorphic differentially expressed genes in 17-month-old THY-Tau22 mice (global analysis across cell types). Nodes represent genes (red = increased expression, blue = decreased expression); edges show regulatory interactions (green arrows = activation, red lines = inhibition). The network comprises 22 nodes and 49 interactions (26 activations, 14 inhibitions). Key regulatory genes appear in the upper part with downstream targets below

For sex-dimorphic DEGs, the THY-Tau22-specific network contains 22 nodes and 40 interactions (see Fig. 9), while the WT network covers 20 nodes and 37 interactions. Central regulatory genes identified include *MBP*, *FOS*, and *JUNB*, which display significant sex-dimorphic expression and regulate substantial portions of the network.

#### Network perturbation analysis (THY-Tau22, 17 months of age)

To identify potential targets for therapeutic disease modification among the regulatory genes in the created GRNs, we performed a network perturbation analysis [25]. The analysis revealed several gene regulators with the potential to modulate and reverse many downstream pathological changes in the network.

In the male-specific GRN, the clusterin gene (*Clu*) was identified as a central regulator with the potential to normalize the expression of 10 downstream DEGs. Another regulator, *Rps9*, showed the capacity to modulate 8 downstream DEGs. When targeted in combination, *Clu* and *Rps9* have the potential for reversing changes in 12 downstream genes (Suppl. Table 16).

For sex-dimorphic networks, both *Hspa5* and *Fos* individually showed a more modest perturbation potential, each capable of modulating 3 downstream DEGs (Suppl. Table S17). However, targeting the combination of *Hspa5* with *Junb* provides a synergistic effect, reversing sex-dependent changes in 10 downstream genes. Visualizations of the condition-specific gene regulatory networks highlighting key regulatory transcription factors and their interactions with direct downstream target genes are shown in Fig. 8 (male-specific GRN) and 9 (sex-dimorphic GRN).

Overall, these analyses reveal a hierarchical organization of sex-dependent gene expression changes in THY-Tau22 mice at 17 months of age, with a few key regulatory genes controlling larger sets of downstream genes. The main identified regulatory hubs include *Clu* for male-specific changes and *Hspa5/Junb* for sex-dimorphic changes.

### Age-dependent cell-type and sex-linked transcriptomic changes in THY-Tau22 mice

#### Age-dependent gene expression alterations in THY-Tau22 mice

The analysis of age-dependent and sex-linked alterations in the THY-Tau22 model revealed extensive cell type-specific patterns of gene expression changes between 7 and 17 months of age. Across nine distinct cell populations, a total of 1,057 genes showed significant disease-associated temporal changes that were either sex-dimorphic or sex-neutral. For detecting more subtle sex-specific changes between age groups, statistical power was insufficient in this analysis.

Among all cell types, microglial cells exhibited the most pronounced temporal changes, with 45 sex-dimorphic and 299 sex-neutral DEGs. Other major cell populations also demonstrated considerable age-dependent changes, though with varying patterns of sex dependence. Oligodendrocytes showed 24 sex-dimorphic and 117 sex-neutral DEGs, while astrocytes had 28 sex-dimorphic and 92 sex-neutral DEGs. Endothelial cells displayed 17 sex-dimorphic and 112 sex-neutral DEGs, suggesting significant vascular involvement in disease progression. Neurons showed only 2 sex-dimorphic DEGs but 96 sex-neutral DEGs, and similarly, OPCs only exhibited few sex-dimorphic DEGs (4), but many sex-neutral DEGs (201).

Overall, while microglia, oligodendrocytes, astrocytes, and endothelial cells displayed a high number of sex-dimorphic changes, only few sex-dimorphic DEGs could be detected in neurons and OPCs.

The smaller cell populations in the dataset showed more limited significant temporal changes, likely due in part to their lower representation in the samples. Macrophages displayed 3 sex-dimorphic and 9 sex-neutral DEGs, while mural cells and ependymal cells showed only sex-neutral changes (7 and 1 DEGs, respectively). Across all cell types, sex-neutral age-dependent changes predominated over sex-dimorphic ones. The complete gene-level statistics for these age-dependent alterations across all covered cell types are provided in Suppl. Table ST5.

Considering the most significant DEGs with known annotations in the individual cell types, in microglia, sex-dimorphic changes were led by *Malat1* (down in males/up in females, FDR = 1.73E-140/<2.11E-280). *Malat1* is a long non-coding RNA involved in inflammation already identified as significant in the cross-sectional analyses across different time points in THY-Tau22 and in the data for the Tg2576 mouse model and human AD (see section on ‘Shared gene-level alterations across mouse models, ages, and human AD’). Further significant age-dependent DEGs included *Cx3cr1* (down in males/up in females, FDR = 1.05E-74/3.77E-22), an essential microglial receptor involved in neuron-microglia communication, and *B2m* (up in males/down in females, FDR = 2.01E-74/4.03E-84), involved in immune response and in aging-related processes as a systemic pro-aging factor [34]. Sex-neutral changes included consistent alterations in *Ctsd* (down in both sexes, FDR = 1.54E-236/<2.11E-280), a cathepsin involved in lysosomal function, in *Cst3* (down in both sexes, FDR = 6.63E-213/<2.11E-280), a key regulator of protein homeostasis already identified as significant in the analysis of THY-Tau22 mice at 17 months of age, and in *Hexb* (down in both sexes, FDR = 6.27E-185/<2.11E-280), a beta-hexosaminidase subunit important for lysosomal function.

In astrocytes, sex-dimorphic patterns again included strong changes in *Malat1* (down in males/up in females, FDR = 4.31E-184/<2.11E-280), *Mt-Nd2* (up in males/down in females, FDR = 5.68E-67/2.41E-21), indicating mitochondrial dysfunction, and *Ccdc88a* (down in males/up in females, FDR = 8.00E-47/1.61E-2), an actin-binding protein involved in cell migration. Sex-neutral changes included *Atp1a2* (down in both sexes, FDR = 1.58E-259/2.30E-81), encoding a Na+/K + ATPase essential for ion transport, *Mt-Nd5* (up in both sexes, FDR = 9.85E-245/2.09E-7), indicating mitochondrial stress similar to *Mt-Nd2*, and *Plpp3* (down in both sexes, FDR = 2.87E-122/6.63E-87), a phosphatase involved in lipid metabolism.

In oligodendrocytes, sex-dimorphic changes were again observed for *Malat1* (down in males/up in females, FDR < 1.58E-259/<2.11E-280), *Fth1* (up in males/down in females, FDR = 9.28E-245/4.43E-82), indicating altered iron homeostasis, and *Car2* (up in males/down in females, FDR = 1.44E-58/9.54E-15), involved in myelin maintenance. Sex-neutral changes featured *Plp1* (down in both sexes, FDR = 2.82E-97/7.52E-12), essential for myelin structure, *Cldn11* (down in both sexes, FDR = 1.02E-93/5.05E-170), important for myelin barrier function, and *Mal* (down in both sexes, FDR = 5.58E-93/1.66E-29), a further gene involved in myelin maintenance. In general, oligodendrocytes showed pronounced changes, in particular in myelin-related genes.

#### Age-dependent pathway alterations in THY-Tau22 mice

The analysis of sex-dimorphic pathway alterations between THY-Tau22 mice at 7 and 17 months of age revealed diverse patterns of enrichment across major brain cell types, with both shared and unique features characterizing the aging response in different cellular populations.

##### Microglial cells

Microglia displayed strong enrichment in protein-related processes, with cytoplasmic translation showing the highest significance (FDR = 1.73E-05, Suppl. Table ST12). At the molecular function level, this was reflected in robust enrichment of ribosome-related functions, including *structural constituent of ribosome* (FDR = 2.06E-09) and *rRNA binding* (FDR = 3.65E-03). Major changes were also observed in protein modification processes, including *regulation of protein ubiquitination* (FDR = 1.29E-02) and *regulation of protein modification by small protein conjugation or removal* (FDR = 1.29E-02).

**Fig. 10:**
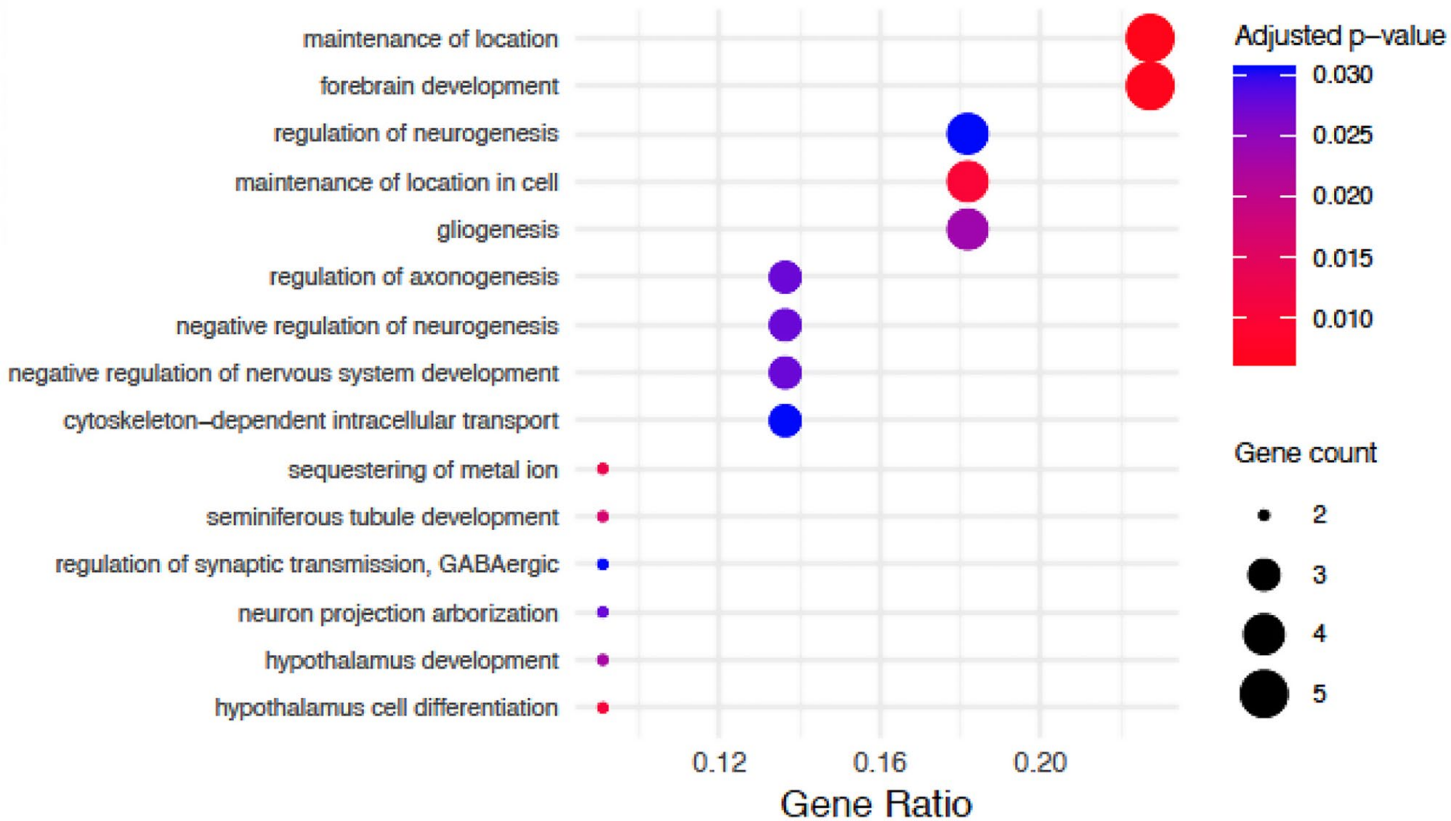
Gene Ontology biological processes enriched in age-dependent sex-dimorphic changes in astrocytes. Dot size indicates number of genes in each pathway; color represents statistical significance (red = more significant). Gene ratio (x-axis) shows proportion of pathway genes differentially expressed. Only pathways with FDR < 0.05 are shown

***Astrocytes***: Astrocytes exhibited strong enrichment in developmental processes, with *gliogenesis* (FDR = 2.36E-02) and *glial cell differentiation* (FDR = 4.45E-02, Suppl. Table ST13) emerging as key pathways during disease progression (see Fig. 10). The molecular function analysis revealed two primary themes: transcriptional regulation and cytoskeletal organization. *Transcription regulator inhibitor activity* showed the strongest molecular function enrichment (FDR = 1.61E-02), while multiple cytoskeleton-related functions were significantly enriched, including *actin binding* (FDR = 1.61E-02) and *microtubule binding* (FDR = 3.58E-02).

##### Oligodendrocytes

**Fig. 11:**
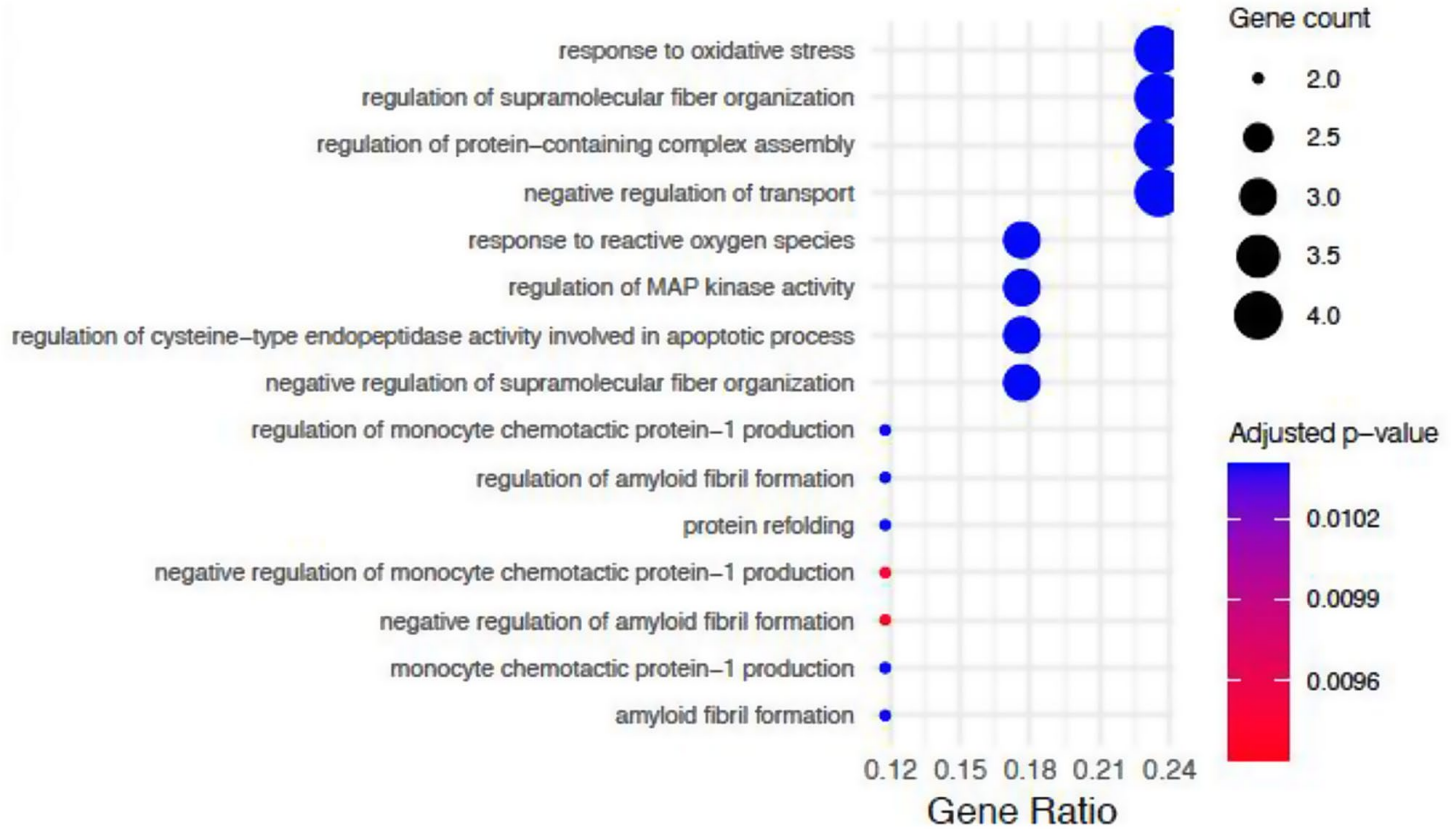
Gene Ontology biological processes enriched in age-dependent sex-dimorphic changes in oligodendrocytes. Dot size indicates number of genes in each pathway; color represents statistical significance (red = more significant). Gene ratio (x-axis) shows proportion of pathway genes differentially expressed. Only pathways with FDR < 0.05 are shown

Oligodendrocytes displayed strong enrichment in protein quality control and stress response pathways (Fig. 11, Suppl. Table ST14). The most significantly enriched biological processes centered around protein aggregation control, including *negative regulation of amyloid fibril formation* (FDR = 9.30E-03) and *response to oxidative stress* (FDR = 1.04E-02). At the molecular function level, *amyloid-beta binding* showed the highest significance (FDR = 1.05E-03), followed by several structural and binding functions including *microtubule binding* (FDR = 1.55E-02) and *fatty acid binding* (FDR = 1.55E-02). The data also revealed enrichment in protein folding and chaperone-related functions, with *unfolded protein binding* (FDR = 1.55E-02) and *heat shock protein binding* (FDR = 3.09E-02) being enriched.

##### Neurons

Neurons showed a distinctive pattern of alterations in regulatory functions (Suppl. Table ST15). The biological process analysis revealed significant enrichment in pathways related to extracellular matrix regulation, with *regulation of blood vessel remodeling* (FDR = 1.20E-02) and *negative regulation of extracellular matrix organization* (FDR = 1.20E-02) being most significant. At the molecular function level, the strongest enrichment results centered around protein regulation, particularly *endopeptidase inhibitor activity* (FDR = 2.53E-02) and *amyloid-beta binding* (FDR = 2.53E-02).

#### Gene regulatory network analysis of age-dependent changes in THY-Tau22 mice

**Fig. 12:**
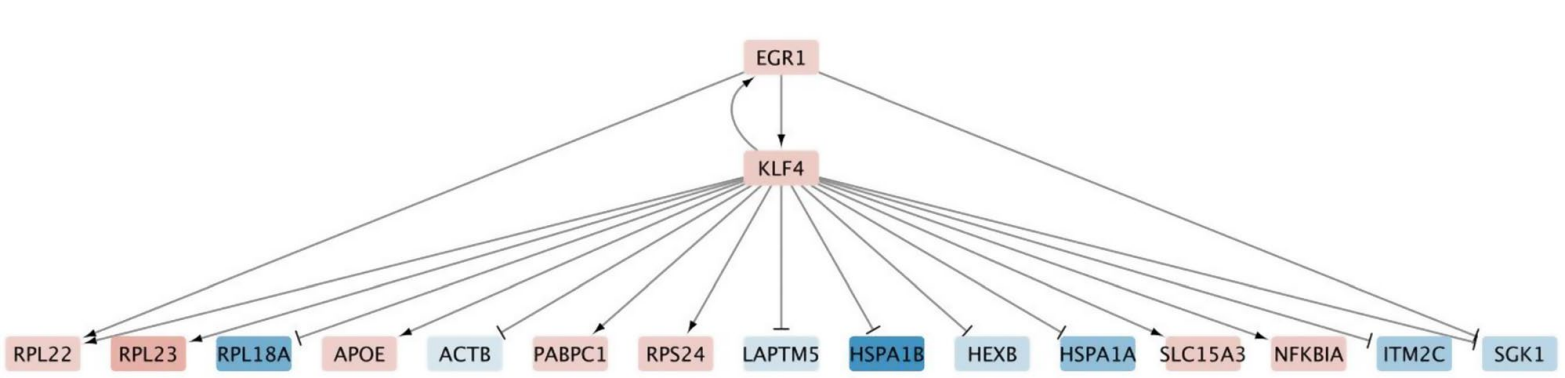
Gene regulatory network of age-dependent transcriptional changes in microglial cells. Network shows relationships between differentially expressed genes in THY-Tau22 mice between 7 and 17 months of age. Node colors indicate expression changes (red = increased, blue = decreased). Edges show regulatory interactions (arrows = activation, perpendicular lines = inhibition). Only the 16 gene interaction partners of Klf4 with lowest p-values are shown to ensure the interpretability of the network

To investigate the regulatory mechanisms underlying age-dependent transcriptomic changes in microglial cells between 7 and 17 months in THY-Tau22 mice, we performed differential gene regulatory network (GRN) analysis on the combined set of DEGs. We analyzed sex-dimorphic and sex-neutral DEGs together, as we noted that these genes function in shared regulatory networks, and analyzing them separately was infeasible due to insufficient DEG numbers for network construction. Using the differential GRN inference approach described in the Methods section, we reconstructed the regulatory network controlling these age-dependent expression changes in microglia, the most abundant cell type in our dataset (see sub-network visualization in Fig. 12).

Network perturbation analysis identified both individual genes and gene pairs whose activity modulation could potentially revert many of the downstream age-dependent changes observed in the THY-Tau22 model. The most potent combination was *Lyl1* and *Klf4*, achieving a joint perturbation score of 70, indicating that modulating these two genes in concert has the potential to revert the age-dependent expression changes of 70 downstream targets. Other two-gene combinations showing equally strong effects included *Ldb1*/*Klf4* and *Ldb1*/*Cited2* (both scoring 70). Among individual genes, *Klf4* and *Cited2* emerged as the regulators with the strongest potential for modulating downstream DEGs, each achieving a single-gene perturbation score of 48, indicating that their modulation alone can reverse a substantial subset of age-dependent changes.

## Discussion

### Interpretation of global expression patterns

Our comprehensive analysis of transcriptomic changes in the THY-Tau22 mouse model and their differences between the sexes revealed several significant alteration patterns in the molecular response to tau pathology. These findings extend previous single-cell studies that have investigated transcriptional changes in tauopathy models [10, 35] and human AD [8, 9, 36] by specifically focusing on the intersection of sex differences and aging. The identified global sex-dependent changes in the pseudobulk analysis at 17 months of age included key pathways relevant to AD pathogenesis. In male-specific responses, we observed pronounced changes in genes involved in lipid metabolism (*Scd2*), iron transport (*Trf*), and RNA processing (*Zc3h13*). These results are consistent with previous studies linking lipid metabolism with tau pathology [37], reported sex differences in brain iron accumulation [38], and findings of dysregulated RNA splicing in tauopathies [39]. The female-specific changes, though fewer, included important chaperone genes such as *Hsp90aa1*, whose altered expression could influence protein folding and cellular stress responses differently in females. This finding is consistent with both the protective nature of many chaperones in experimental models of neurodegeneration [40], and with the previously reported AD-associated protein expression changes in the human ortholog HSP90AA1 in the entorhinal cortex [29].

Overall, there was a clear predominance of male-specific over female-specific transcriptional changes at 17 months of age, with males showing a much stronger transcriptional response to tau pathology. This observation cannot be attributed to different levels of gene expression variability, as the standard deviations of expression levels were comparable between males and females. While this aligns with growing evidence that male and female brains may respond differently to neurodegenerative conditions [41, 42], this male-biased response pattern is particularly interesting given that human AD typically shows higher age-adjusted prevalence in females [43], and warrants careful interpretation in the context of disease progression. As noted in our Introduction, while females show higher overall AD prevalence and often experience faster cognitive decline, males tend to die faster with AD and show lower brain resilience to the effects of tau pathology [5]. When comparing our findings at 17 months with the earlier 7 months time point, the extensive male-specific transcriptional changes at this late disease stage may therefore reflect the cumulative impact of chronic tau pathology on male brains. Indeed, in previous studies in tauopathy mice, males showed greater susceptibility to pathology and drug-resistant microglia [44], whereas females may be more susceptible to other disease manifestations.

However, the observed stronger transcriptional response in males may in part also reflect compensatory mechanisms in males that might contribute to their relative protection against AD [36]. Given the enrichment of male-specific transcriptional changes in protein quality control pathways and coordinated inflammatory responses (rather than chronic inflammatory responses), at least some of these alterations may be beneficial. This interpretation aligns with the lower AD prevalence in human males [43] and suggests that key transcriptional changes may represent protective mechanisms, though they may become insufficient or even detrimental as the pathology progresses.

In general, the age- and sex-dependent significant molecular changes observed in THY-Tau22 mice indicate that the relationship between transcriptional responses and tau pathology varies with both sex and stage of pathology. This underscores the importance of considering both sex-specific mechanisms and the progression of tauopathy when developing therapeutic strategies, as the same pathological processes can elicit distinct molecular responses in men and women at different disease stages. In addition, the substantial number of sex-dimorphic DEGs showing opposing directions of change between males and females further highlights that biological sex fundamentally influences cellular responses to tau pathology. Overall, these findings indicate that at least some aspects of tau pathology might benefit from sex-specific treatment approaches, which would have important implications for future drug development and dosage regimens.

### Cell Type-Specific response patterns

The single-cell analysis highlighted divergent patterns of sex-dependent responses across major brain cell types, with particularly pronounced effects in microglia and astrocytes.

The predominance of DEGs with sex-linked patterns in glial cells suggests a sexually dimorphic neuroimmune response to tau pathology. This finding matches with the growing recognition of neuroinflammation as a key driver of AD progression [45], and with studies demonstrating sexual dimorphism in microglial development and function [46, 47] and astrocytic structure and function [48, 49]. The male-specific upregulation of inflammatory and immune response genes (such as *H2-D1* and *Tyrobp*) in microglia, contrasting with female-specific stress response changes (*Hsp90ab1*), may reflect different immune response strategies between sexes that are regulated by sex hormones [50].

Astrocytes displayed both strong male-specific responses and the highest number of sex-dimorphic DEGs, highlighting sex-dependent regulation of key astrocytic functions. Male-specific changes in genes regulating protein homeostasis and barrier function (*Clu*, *Cldn10*, *Cpe*) contrasted with female-specific alterations in RNA processing (*Pnisr*), indicating sex-specific adaptation mechanisms for astrocytes, which have previously already been reported for ischemia-induced damage [51].

Oligodendrocytes emerged as another cell type with strong sex-dependent responses, with predominant male-specific changes affecting myelin maintenance and bioenergetic pathways. Specifically, males showed significant alterations in genes regulating myelin remodeling (*Car2*) and mitochondrial function (*Mt-Nd1*, *Mt-Nd2*), suggesting sex-specific vulnerabilities in myelin integrity and cellular metabolism. These findings align with growing evidence for white matter involvement in AD pathogenesis [52] and indicate that myelin-targeted therapeutic approaches may require sex-specific optimization.

Neurons showed sex-dependent changes in particular in mitochondrial and matrix proteins in males (*mt-Cytb*, *Sparcl1*) and amyloid precursor protein processing in females (*App*). This differential involvement of bioenergetic, matrix, and amyloid-related processes indicates distinct patterns of neuronal dysregulation in key AD-associated pathways between sexes. Given its central role in AD pathogenesis, the female-specific modulation of *App* is particularly intriguing and matches with prior findings showing that *App* is regulated by sex hormones in the cortex [53].

These cellular responses also showed distinct age-dependent patterns when comparing THY-Tau22 mice at 7 and 17 months of age: microglia demonstrated progressive changes in protein synthesis and modification pathways that differed between sexes, while astrocytes and oligodendrocytes showed sex-linked and age-dependent changes in developmental processes, and respectively, in stress response pathways. The substantial microglial and astrocytic response matches with the growing recognition of neuroinflammation as a relevant mechanism in AD progression [45]. Neurons displayed sex-dependent changes across age groups primarily in extracellular matrix regulation and protein regulatory functions, particularly involving blood vessel remodeling and endopeptidase inhibition, which may reflect vascular dysfunction [54] and altered protein degradation in AD [55]. However, changes in neurons were less pronounced than in microglia. This contrast in sex-dependent patterns across cell types indicates that immune responses in AD may be more sex-dependent than core neuronal functions, potentially reflecting known sex differences in neuroimmune responses and inflammatory signaling pathways in the brain [56, 57]. Across all cell-types, sex-neutral age-dependent changes dominated over sex-dependent changes, suggesting that while sex-specific responses are important, many fundamental disease-associated changes occur similarly in both males and females across age groups.

Taken together, these age-dependent changes reveal a complex interplay between sex-dependent and sex-independent adaptations to tau pathology over time, particularly in pathways related to energy metabolism, protein homeostasis, and inflammatory responses. The findings indicate that while certain pathological processes progress similarly in both sexes over the two time points, others show marked sex-dependent divergence.

### Cross-Species and Cross-Model relevance

Our comparative analysis of differentially expressed genes across mouse models, ages, and human AD revealed surprisingly limited overlap between datasets (Fig. 7). This limited concordance warrants careful consideration when interpreting the relevance of mouse models to human disease.

Several methodological factors may contribute to these minimal overlaps. First, technical differences in single-cell technologies (DropSeq for our mouse studies versus 10X Genomics for the human data) can introduce platform-specific biases [58]. Second, differences in sequencing depth, bioinformatic processing pipelines, and statistical thresholds for identifying differential expression may all influence which genes are detected as significantly altered. Third, technical limitations for cell types that are highly heterogeneous and undergo degeneration, such as neurons, may result in a higher variability and an underrepresentation in single-cell studies due to increased fragility during tissue dissociation, leading to a reduced ability to detect shared DEGs.

Beyond technical considerations, fundamental biological differences are important. Human neurons differ fundamentally from mouse neurons, being larger, more complex, with greater dendritic and synaptic complexity, and longer maturation periods [59, 60]. Human brains possess unique neuronal subtypes, particularly among pyramidal neurons [61] and interneurons [62] that are abundant in the neocortex. Moreover, the human AD samples represent end-stage disease in elderly individuals, while even our 17-month-old mice represent middle-to-late adulthood rather than advanced age. Species-specific differences in gene expression regulation, cellular composition, and response to pathology further complicate direct comparisons. Additionally, the THY-Tau22 model expresses FTD-associated tau mutations rather than representing the mix of tau pathologies seen in sporadic human AD [63].

A small overlap was also observed between our 7- and 17-month THY-Tau22 datasets. This suggests that different molecular pathways predominate at distinct ages or pathology stages, with early responses potentially involving different gene sets than later compensatory or degenerative changes, in line with findings for other model systems and tissues [64, 65]. This stage-specific molecular signature may have important implications for preclinical therapeutic development, as interventions targeting early-stage molecular changes may be ineffective at later stages. These limitations highlight the need for cautious interpretation when extrapolating molecular observations from the considered mouse models to human disease.

However, the comparison of transcriptomic changes across mouse models, ages, and human AD also revealed conserved features that strengthen their potential relevance to AD pathogenesis. Of particular interest is the consistent alteration of *Malat1* across species and models, especially in oligodendrocytes. This long non-coding RNA’s conserved dysregulation, with a dominant pattern of male-specific underexpression, combined with its role in synaptic plasticity and cognition [66, 67], indicates a potential involvement in mediating sex-specific changes in AD pathology.

The shared patterns in myelin-related gene expression, particularly *Mbp*’s consistent male-specific changes across models and human data, highlight the importance of white matter pathology in AD. The variation in *Mbp* expression patterns between different age groups in the THY-Tau22 model points to a dynamic regulation that may reflect disease progression stages, potentially offering temporal windows for pro-myelinating therapeutic approaches currently under investigation [68, 69].

Heat shock protein family members, particularly *Hsp90ab1*, also showed notable conservation among the significant findings across datasets, but with varying sex-dependent patterns. This variability in sex-specificity between models and human data could result from a different regulation of stress response mechanisms across species and disease stages [70]. Finally, the consistent alterations of the ATPase *Atp1b1* across datasets, particularly in neurons, points to fundamental disruptions in cellular energy metabolism and ion homeostasis [71].

Although our study used the THY-Tau22 model expressing mutations originally identified in FTD cases (G272V and P301S) [13], we compared our findings with human AD data. This cross-disease comparison is valuable because tau aggregation is a common pathological feature in both AD and FTD, albeit with differences in tau isoform ratios and distribution patterns. The conservation of certain molecular signatures between the THY-Tau22 model and human AD data, particularly genes such as *Malat1* and *Mbp*, suggests common pathogenic mechanisms in tau-mediated neurodegeneration across tauopathies [72]. However, divergent patterns observed between our model and human AD data may reflect disease-specific processes unique to either AD or FTD. Future research would benefit from comparative analyses across multiple tauopathy models representing different diseases to further delineate common and disease-specific transcriptomic signatures.

Taken together, these cross-species and cross-model comparisons reveal both opportunities and challenges for follow-up research. While the conservation of certain molecular changes supports their targeting potential, the varying patterns of sex-dependency across models and species suggest careful consideration is needed when translating findings from animal models to human therapeutic research.

### Disentangling aging and disease progression in the THY-Tau22 model

The limited overlap between DEGs in 7-month and 17-month THY-Tau22 mice raises important questions about the relationship between aging and disease progression in this model. As age is the key difference between these two groups, the transcriptomic differences we observe are fundamentally age-associated, but represent a complex combination of normal aging processes, age-dependent progression of tau pathology, and age-dependent compensatory responses.

Our study design, which includes age-matched wildtype controls at both time points, allows us to partially distinguish between normal aging effects and disease-specific changes. The differential expression analysis at each time point compares age-matched transgenic and wildtype mice, theoretically controlling for normal aging processes and highlighting disease-associated changes. However, this approach cannot fully account for interaction effects between aging and disease, where normal aging processes may differently affect cells already experiencing tau-mediated stress.

The small DEG overlap likely represents fundamentally different molecular states at early versus late disease stages. The 7-month timepoint captures initial cellular responses to emerging tau pathology, while the 17-month timepoint reflects advanced pathology with extensive tau aggregation, synaptic loss, and compensatory mechanisms, each engaging distinct gene sets despite the same underlying pathology.

However, normal aging processes contribute substantially to these differences. Mouse brains have been shown to undergo significant cell type-specific transcriptomic remodeling with age [73], with different tissues and cell types epigenetically estimated to age at varying rates and through distinct molecular mechanisms [74]. Many aging-related pathways, including inflammation, oxidative stress, and proteostasis, overlap with disease-associated pathways, further complicating the distinction. Moreover, as sex differences have been reported in the onset of age-related inflammatory changes in mice [75], the stronger male-specific transcriptional response at 17 months may be influenced by sexual dimorphism in brain aging, potentially amplifying sex differences in disease processes. While we cannot completely separate pure aging from disease progression effects, our distinct findings for the two time points suggest that tau pathology and its interaction with normal aging engage temporally distinct molecular programs.

### Comparison with other Tau Transgenic models

To further validate our findings and establish their broader relevance to tau-mediated pathology, we compared our key differentially expressed genes with published data from other tau transgenic mouse models. While most previous studies have not investigated sex-specific transcriptomic changes or provided raw data stratified by sex, and have only reported bulk expression statistics, comparing transgenic versus wild-type differential expression across models can still provide valuable insights into conserved molecular signatures of tauopathy.

When examining bulk RNA-seq statistics reported by Kim et al. for the TPR50-P301S mouse model [76], we found consistent significance and direction of changes for multiple of the most significant DEGs identified in our study. Specifically, *Mbp* (log2FC = 0.41, FDR = 6.1E-10), *Fth1* (log2FC = 0.25, FDR = 1.3E-04), *Trf* (log2FC = 1.54, FDR = 7.5E-31), and *Apod* (log2FC = 1.7, FDR = 6.8E-27) showed concordant directional changes between the models. However, *Plp1* (log2FC = 0.50, FDR = 9.2E-13) and *Hsp90aa1* (log2FC = -0.23, FDR = 6.6E-05) displayed opposite directional changes, suggesting model-specific or context-dependent roles for these genes.

Further comparison with expression statistics published for the rTg4510 model by Castanho et al. [77] in entorhinal cortex tissue revealed concordance for most of the DEGs covered in both studies, including *Mbp* (log2FC = 0.003, FDR = 3.3E-03), *Fth1* (log2FC = 0.02, FDR = 2.8E-06), *Scd2* (log2FC = 0.009, FDR = 0.03), *Trf* (log2FC = 0.06, FDR = 4.7E-12), *Cst3* (log2FC = 0.06, FDR = 5.4E-17), and *Apod* (log2FC = 0.03, FDR = 2.5E-03). Among the reported DEGs, only *Zc3h13* (log2FC = -0.01, FDR = 3.0E-05) showed an opposite direction of change compared to our model.

Taken together, while these previous tauopathy mouse model datasets lack sex-specific and cell type-specific information, the cross-model comparison strengthens the biological relevance for multiple of our most significant DEGs by identifying a core set of consistently dysregulated, high-confidence DEGs across different tau models despite variations in the specific tau mutations, promoters used, and experimental designs. The concordant changes in genes related to iron homeostasis (*Fth1*, *Trf*), lipid metabolism (*Apod*, *Scd2*), and myelin maintenance (*Mbp*) also confirm the relevance of key pathways as a conserved response to tau pathology. Conversely, genes that show model-specific changes, such as *Plp1* and *Hsp90aa1*, may reflect differences in the stage of pathology, tau isoform composition, or model-specific compensatory mechanisms. These comparisons highlight both the most robust alterations associated with tau pathology among our findings and the complexity of molecular responses across different models of this pathology.

### Pathway-Level insights

Our pathway analysis highlighted numerous significant biological process alterations across cell types, providing insights into the cellular mechanisms underlying sex-dependent responses to tau pathology. The cell type-specific enrichment results revealed both shared and unique aspects of the disease process across different brain cell populations.

In microglial cells of THY-Tau22 mice at 17 months of age, the enrichment of synaptic and inflammatory response pathways in sex-dimorphic DEGs, coupled with male-specific enrichment in the regulation of neuron apoptotic processes and amyloid-beta formation pathways, indicates a sex-dependent regulation of key AD-related processes, in line with previous findings by the authors [78]. The strong enrichment of the molecular functions “tau protein binding” and “ubiquitin protein ligase binding” in male-specific changes suggests that protein quality control mechanisms, which are known to play a key role in AD [79], have strongly modulated activity in male microglia.

Oligodendrocyte pathway analysis revealed myelination and chemokine production changes in male-specific DEGs, while sex-dimorphic DEGs showed enrichment in lipid metabolism and membrane organization pathways. This suggests that while myelin maintenance may be particularly affected in males, the fundamental processes of lipid metabolism show dysregulation across both sexes, but with diverging patterns. These findings match with the growing evidence for white matter involvement in AD [52](see also the sections on the gene level analysis and cross-species and cross-omics comparison above) and sex differences observed in lipid metabolism in patients with mild cognitive impairment [80].

The astrocyte pathway analysis highlighted an enrichment of male-specific DEGs in amyloid beta regulation and CNS myelination, while sex-dimorphic DEGs were enriched in cellular stress response pathways.

In neurons, the enrichment of sodium ion homeostasis and transmembrane transport pathways in sex-dimorphic DEGs, coupled with male-specific enrichment in symporter activity and glutamate binding, suggests that sex-specific regulatory mechanisms may influence neuronal excitability and synaptic function, matching with previous reports of sex-dependent differences in neuronal plasticity [81].

The age-dependent analysis of sex-dimorphic pathway alterations between 7 and 17 months revealed distinct temporal patterns. In microglia, changes primarily influenced protein-related processes, with cytoplasmic translation and protein modification pathways showing opposing regulation between sexes over time. Astrocytes exhibited age-dependent sex-linked changes in developmental processes and transcriptional regulation, with gliogenesis and transcription regulator inhibitor activity showing particularly strong enrichment. In oligodendrocytes, protein quality control and stress response pathways showed sex-dependent changes, particularly in the regulation of amyloid fibril formation and oxidative stress response, with well-established AD associations [82, 83]. Neurons displayed sex-dimorphic age-dependent changes in extracellular matrix regulation and protein inhibitory functions, especially in blood vessel remodeling and endopeptidase inhibitor activity.

Overall, the pathway-level insights confirm the findings from the gene-level analysis, highlighting a complex interplay between sex-dependent and sex-independent process alterations across cell types. While certain pathways show clear sex-specific regulation, others display more subtle sex-dimorphic patterns that might contribute to the observed sex differences in AD prevalence and progression. These findings corroborate that pathway-targeted therapeutic interventions might need to consider cell type, age, and sex-specific factors.

### Regulatory network implications

The GRN analysis highlighted a hierarchical organization of sex-dependent transcriptional responses to tau pathology, with few transcription factors modulating the expression levels of many downstream genes. This network-level organization provides insights into potential therapeutic intervention points and suggests regulatory mechanisms for sex-specific disease progression.

In the global male-specific network, the emergence of clusterin (*Clu*) as a central regulatory hub is of particular interest given its known role as an AD risk factor [33]. Since clusterin has recently already been proposed as a therapeutic target for AD [84], its regulatory potential to reverse the expression of multiple downstream sex-dependent DEGs indicates that it could also serve as a candidate target to address sex-specific pathological changes in AD.

In addition, the sex-dimorphic network analysis highlighted the *Hspa5/Junb* regulatory axis as a potential master regulator of sex-dependent responses. The synergistic effects observed in the network perturbation analysis when modulating these regulators in combination matches with their joint involvement in T-cell receptor activation [85] and the implication of T-cell dysfunction in AD [86].

In microglial cells, the regulatory network revealed *Klf4* as a central hub, particularly in controlling age-dependent changes. *Klf4’s* prominence in both individual and paired perturbation analyses, along with its central position in the regulatory network, indicates a potential role as a key mediator of age-dependent microglial responses to tau pathology. This transcription factor forms a regulatory feedback loop with *Egr1* in the network, reminiscent of the regulatory pattern observed in our previous cross-sectional analyses of THY-Tau22 mice at 7 months of age [12] and of the Tg2576 model of amyloid-beta pathology [14]. The previous implication of *KLF4* and *EGR1* in human AD [87, 88] and the conservation of this *Klf4*-*Egr1* regulatory module across different time points and between tau- and amyloid-based AD models points to its fundamental importance in the pathological process.

*Cited2*, matching *Klf4*’s individual network perturbation score, has been implicated in neuroinflammatory responses and microglial activation. Previous studies have shown that *Cited2* is part of a negative feedback mechanism regulating NF-κB signaling [89], a central pathway associated with inflammatory responses in microglia. Its strong perturbation effects suggest a significant contribution to age-dependent microglial responses to tau pathology.

*Lyl1*, a basic helix-loop-helix transcription factor, demonstrated modest individual regulatory effects (perturbation score of 12) but showed notable synergistic potential with *Klf4*. This could reflect a cooperative role in modulating microglial activation states, given *Lyl1*’s established function in regulating microglia development and immune-modulatory phenotypes in primitive macrophage progenitors [90].

Overall, the results of the network analysis provide new insights into the regulatory mechanisms involved in age-dependent transcriptional changes in microglia in THY-Tau22 mice. They highlight the regulators *Klf4* and *Egr1*, whose involvement in modulating tau pathology associated changes was already supported by our previous cross-sectional analyses of the THY-Tau22 model at 7 months of age [12]. These regulators may serve as putative targets for modulating microglial responses in tau pathology, particularly given their potential to revert expression changes for several downstream DEGs.

## Conclusions

Our comprehensive cross-sectional and age-dependent single-cell transcriptomic analysis of the THY-Tau22 mouse model revealed extensive sex-linked molecular changes associated with tau pathology. The key findings highlight that biological sex significantly influences disease-associated transcriptional responses in multiple cell types, with particularly pronounced effects in microglia, astrocytes, and oligodendrocytes.

The study identified several patterns of interest: (1) a predominance of male-specific over female-specific transcriptional changes at 17 months - if protective, this would be consistent with a lower prevalence of AD in human males; (2) substantial sex-dimorphic gene expression, particularly in stress response, protein homeostasis, and neurotransmitter signaling pathways; (3) conservation of certain sex-dependent changes across mouse models and human AD data, including *Malat1*; (4) identification of potential targets for sex-specific therapeutic intervention through network analysis, including *Clu* and *Egr1*/*Klf4*.

Important limitations include the focus on two time points, the need to further investigate the relationship between male-biased transcriptional responses and the female prevalence of human AD, and the need to validate candidate therapeutic targets. Therefore, future research should focus on: (1) investigation of additional time points; (2) further comparative studies with human AD tissue; (3) preclinical functional validation of identified sex-specific therapeutic targets.

Collectively, our findings demonstrate that tau pathology triggers significant sex-specific molecular responses, with age-dependent and cell-type-specific patterns. This highlights that effective AD therapies may need to consider biological sex as a key factor in drug development and clinical trial design. Finally, the age-dependent aspects of sex-related changes suggest that therapeutic strategies may need to be tailored not only by sex but also by disease stage, representing a further step toward more effective, personalized therapeutic approaches for AD and related tauopathies.

## Electronic supplementary material

Below is the link to the electronic supplementary material.

Supplementary Material 1

Supplementary Material 2

## Data Availability

The datasets supporting the conclusions of this article are available in the NCBI Gene Expression Omnibus (GEO) repository, including the THY-Tau22 mouse model data for mice at 7 months of age (accession GSE245035, https://www.ncbi.nlm.nih.gov/geo/query/acc.cgi?acc=GSE245035), the THY-Tau22 mouse model data for mice at 17 months of age (accession GSE285506, https://www.ncbi.nlm.nih.gov/geo/query/acc.cgi?acc=GSE285506), the Tg2576 mouse model data (accession GSE285694, https://www.ncbi.nlm.nih.gov/geo/query/acc.cgi?acc=GSE285694), and the data from the brain of human AD patients and controls (accession GSE138852, https://www.ncbi.nlm.nih.gov/geo/query/acc.cgi?acc=GSE138852).

## Code availability

All scripts used for data analysis are available on GitLab: https://gitlab.com/uniluxembourg/lcsb/biomedical-data-science/bds/thy-tau22-longitudinal-analysis.
