# Supplementary material for: Temporal transcriptomic changes in the THY-Tau22 mouse model of tauopathy display cell type- and sex-specific differences
Source: Acta Neuropathol Commun. 2025 May 7;13:93. doi: 10.1186/s40478-025-02013-z (PMC12060421; doi:10.1186/s40478-025-02013-z)
Supplement: Supplementary file 1 — Supplementary Material 1 [file 40478_2025_2013_MOESM1_ESM.pdf]

Supplementary Materials

Muhammad Ali[1], Pierre Garcia[1], Laetitia P. Lunkes[1], Alessia Sciortino[1], Melanie Thomas[1], Tony Heurtaux[2], Kamil Grzyb[1], Rashi Halder[1], Alex Skupin[1], Luc Buée[3], David Blum[3], Manuel Buttini[1], Enrico Glaab[1]

Affiliations:
[1] Luxembourg Centre for Systems Biomedicine (LCSB), University of Luxembourg, 7 avenue des Hauts Fourneaux, L-4362, Esch-sur-Alzette, Luxembourg
[2] Department of Life Sciences and Medicine (DLSM), University of Luxembourg, 6 avenue du Swing, L-4367, Belvaux, Luxembourg
[3] Neuroscience & Cognition Research Centre, INSERM, CHU Lille, LabEx DISTALZ, UMR-S 1172, Alzheimer & Tauopathies, Université Lille, Lille, France

Supplementary Figures

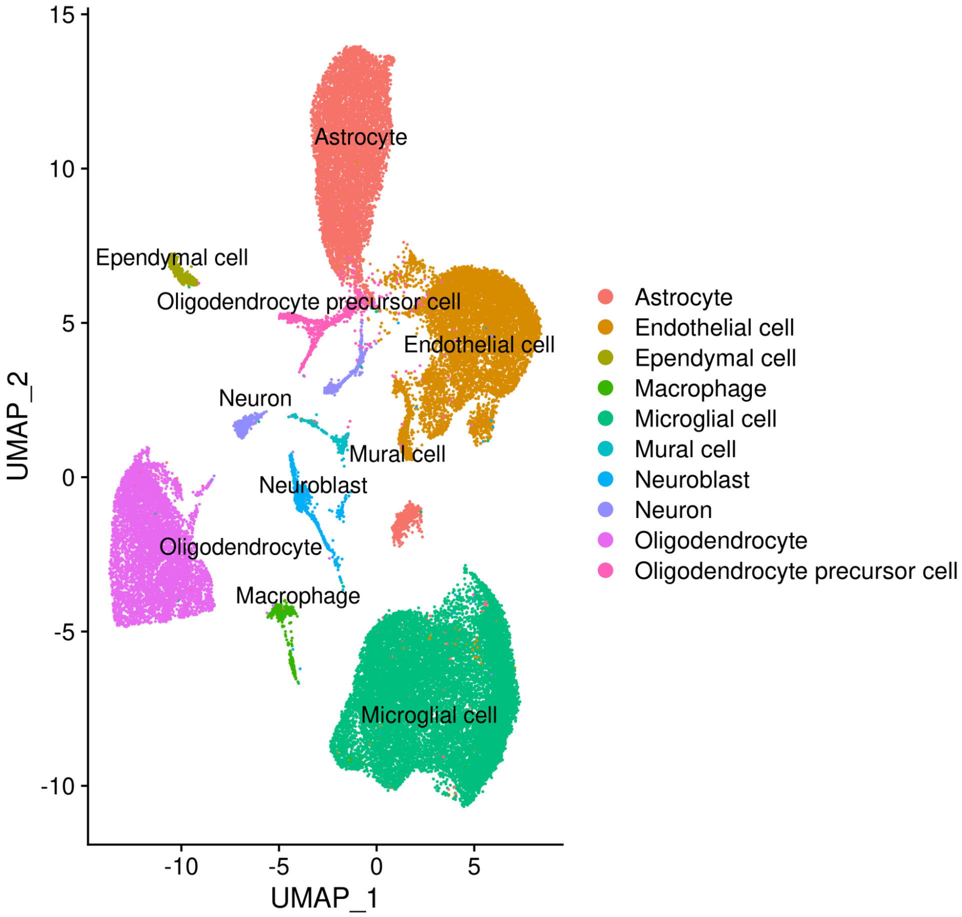

Fig. S1: Visualization of scRNA-seq data clustering at 7 months of age. UMAP visualization of cell clusters representing distinct cell types in cortical tissue from THY-Tau22 and wild-type mice at 7 months of age. Cell type annotation was performed using SCType. The plot integrates data from all mice across both sexes (males and females) and conditions (transgenic and wild type). This figure complements Fig. 2 in the main text which shows the corresponding data for 17-month-old mice.

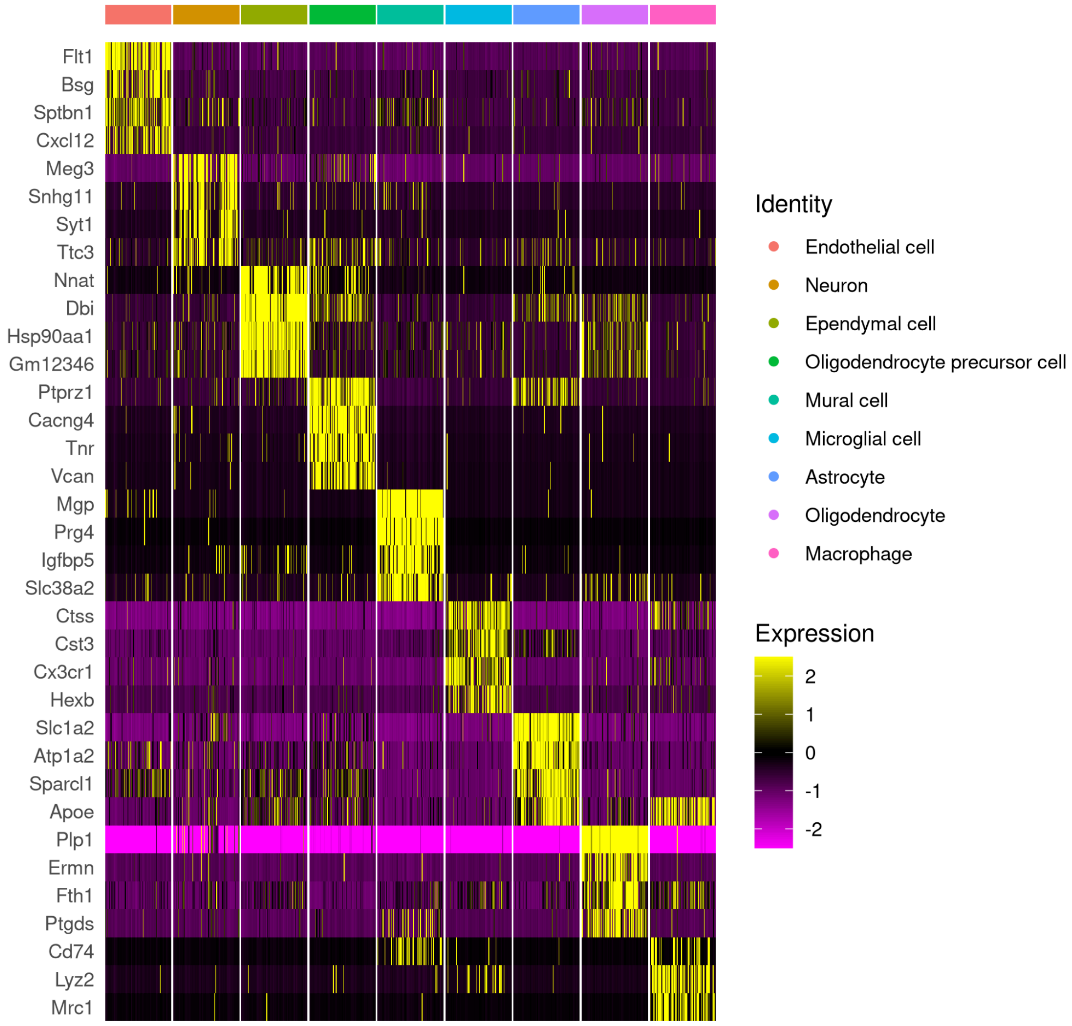

Fig. S2: Heatmap of cell type-specific marker gene expression. Expression patterns of established cell type marker genes across identified cell clusters. Color intensity represents normalized expression levels, with yellow indicating high expression and pink indicating low expression. Hierarchical clustering of both rows (genes) and columns (cell types) reveals distinct expression signatures for each cell population.

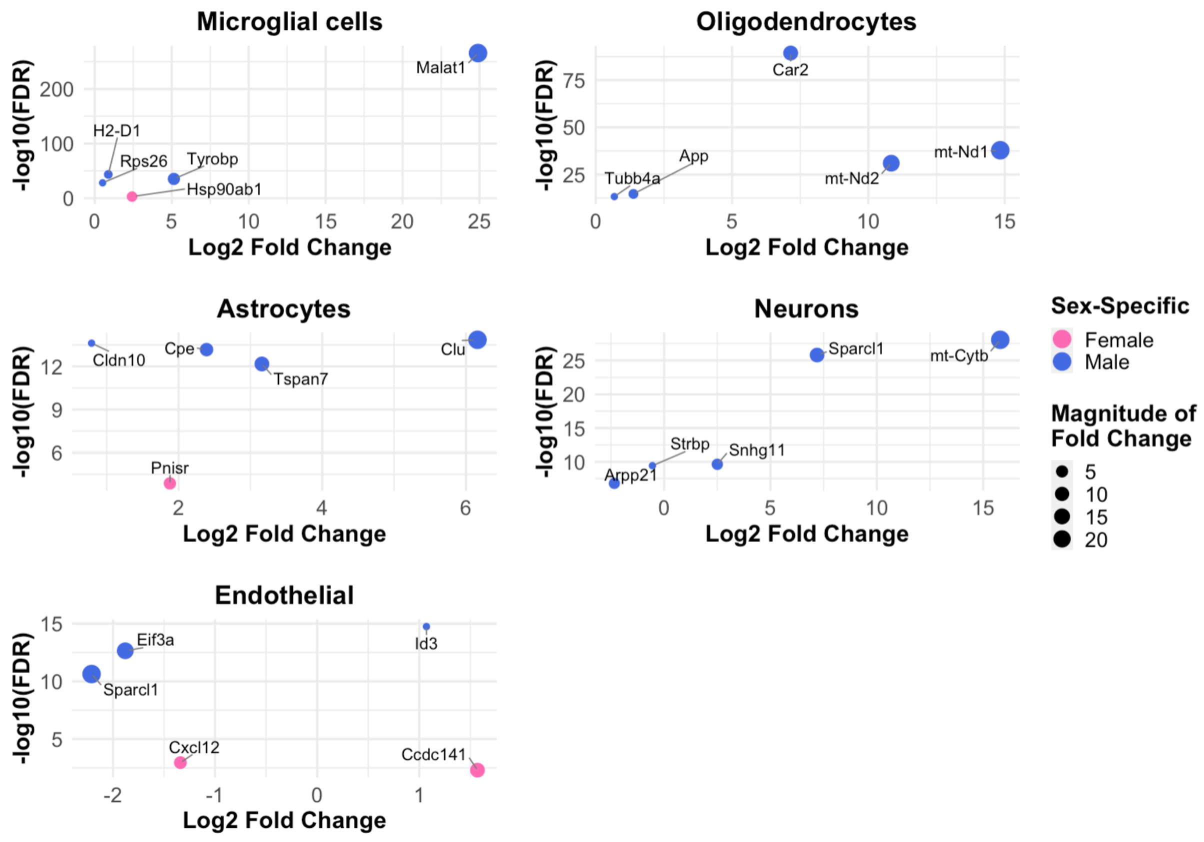

Fig. S3: Sex-specific differential gene expression patterns across five main brain cell types. Volcano plots depicting differentially expressed genes (DEGs) in five main brain cell types. The x-axis shows the log2 fold change (logFC), with positive values indicating higher expression in males and negative values indicating higher expression in females. The y-axis shows statistical significance as -log10(FDR). Point size corresponds to the magnitude of fold change, while colors indicate sex-specific regulation (blue = male-specific, pink = female-specific). Key genes of interest are labeled, including inflammatory response genes in microglia (e.g., Malat1, H2-D1, Tyrobp), protein homeostasis genes in astrocytes (e.g., Clu, Cldn10), mitochondrial genes in neurons (e.g., mt-Cytb) and oligodendrocytes (e.g., mt-Nd1, mt-Nd2), and regulatory genes in endothelial cells (e.g., Id3, Cxcl12).

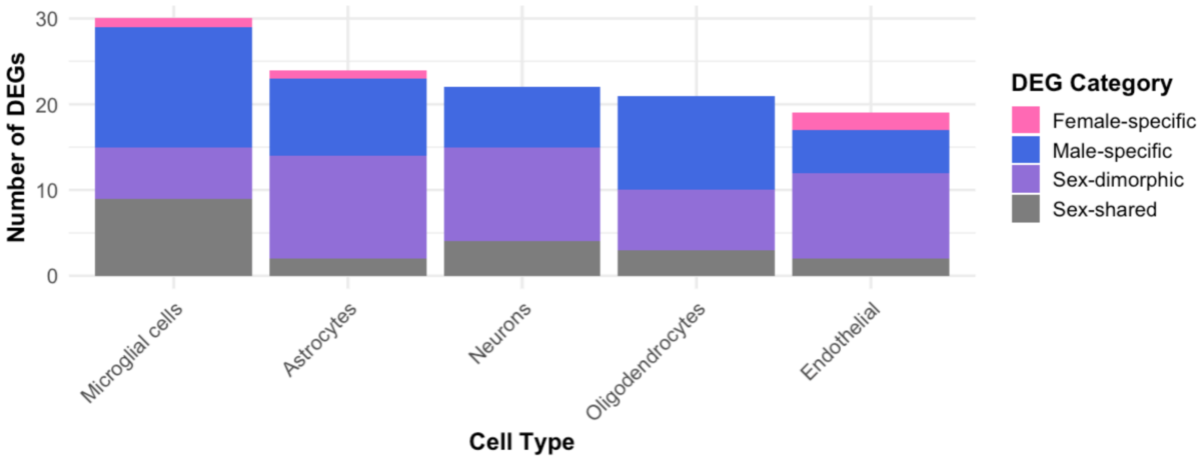

Fig. S4: Distribution of differentially expressed genes across five main brain cell types. Stacked bar plot showing the number and categories of differentially expressed genes (DEGs) in each brain cell type at 17 months of age. The bars are subdivided into four categories: male-specific (blue), female-specific (pink), sex-dimorphic (purple), and sex-neutral (gray) DEGs. Microglial cells show the highest number of total DEGs (55), with a strong bias toward male-specific expression (37 genes). Oligodendrocytes uniquely lack female-specific DEGs but show substantial male-specific expression (17 genes). Neurons and astrocytes display more balanced distributions between sex-dimorphic and sex-specific genes. Endothelial cells show the lowest total number of DEGs (23) but maintain representation across all categories

.

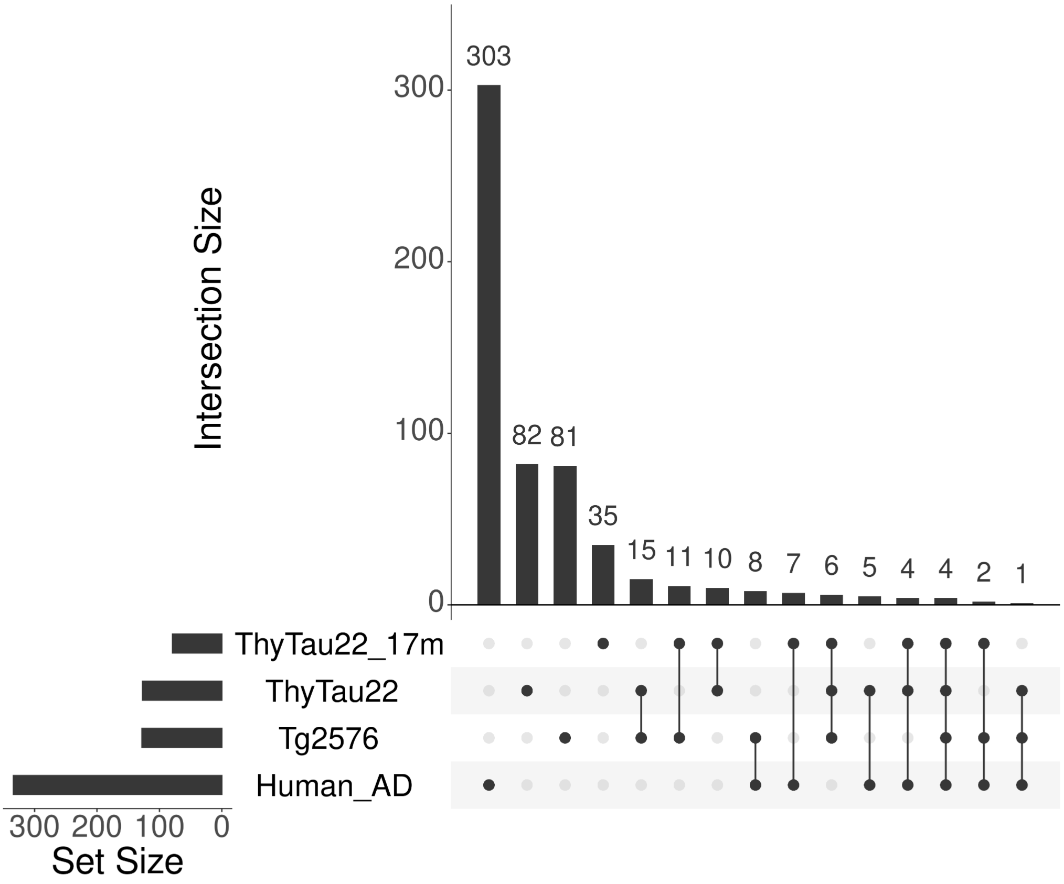

Fig. S5: Overlap of differentially expressed genes between datasets. UpSet plot showing the intersection of differentially expressed genes across four datasets: THY-Tau22 mice at 7 and 17 months, Tg2576 mice, and human AD cortical tissue. The bar chart indicates the size of each intersection set, while the connected dots below show which datasets contribute to each intersection.

Global sex-dependent pathway alterations

We performed pathway enrichment analysis of the sex-dependent DEGs at the level of global changes across all cell types (pseudobulk analysis) for THY-Tau22 mice at 17 months of age (see Methods). Since the number of female-specific DEGs was insufficient to identify cellular process alterations linked to female-specific changes, in the following we focus on identified pathways enriched in male-specific and sex-dimorphic DEGs.

Global male-specific pathway enrichment (THY-Tau22, 17 months)

The pseudobulk enrichment analysis of the male-specific DEGs identified in the combination of cell types revealed distinct patterns of dysregulation in biological processes and molecular functions from the Gene Ontology database (see Fig. 3). The most significantly enriched biological processes centred on RNA processing and splicing pathways, with several related terms showing strong enrichment (adjusted $p<0.05$). Other enriched processes included erythrocyte and myeloid cell homeostasis and regulation of synaptic potentiation, suggesting broad effects on both cellular maintenance and neuronal function.

At the molecular functional level, the analysis revealed a significant enrichment of chromatin and histone-related activities, in particular protein binding and various methyltransferase functions (adjusted $p<0.05$). The prominence of RNA processing and epigenetic regulation pathways suggests that male-specific responses to tau pathology at the 17-months time point may particularly affect gene expression control and chromatin modification. A comprehensive list of all enriched pathways and their statistical parameters can be found in Suppl. Table 4.

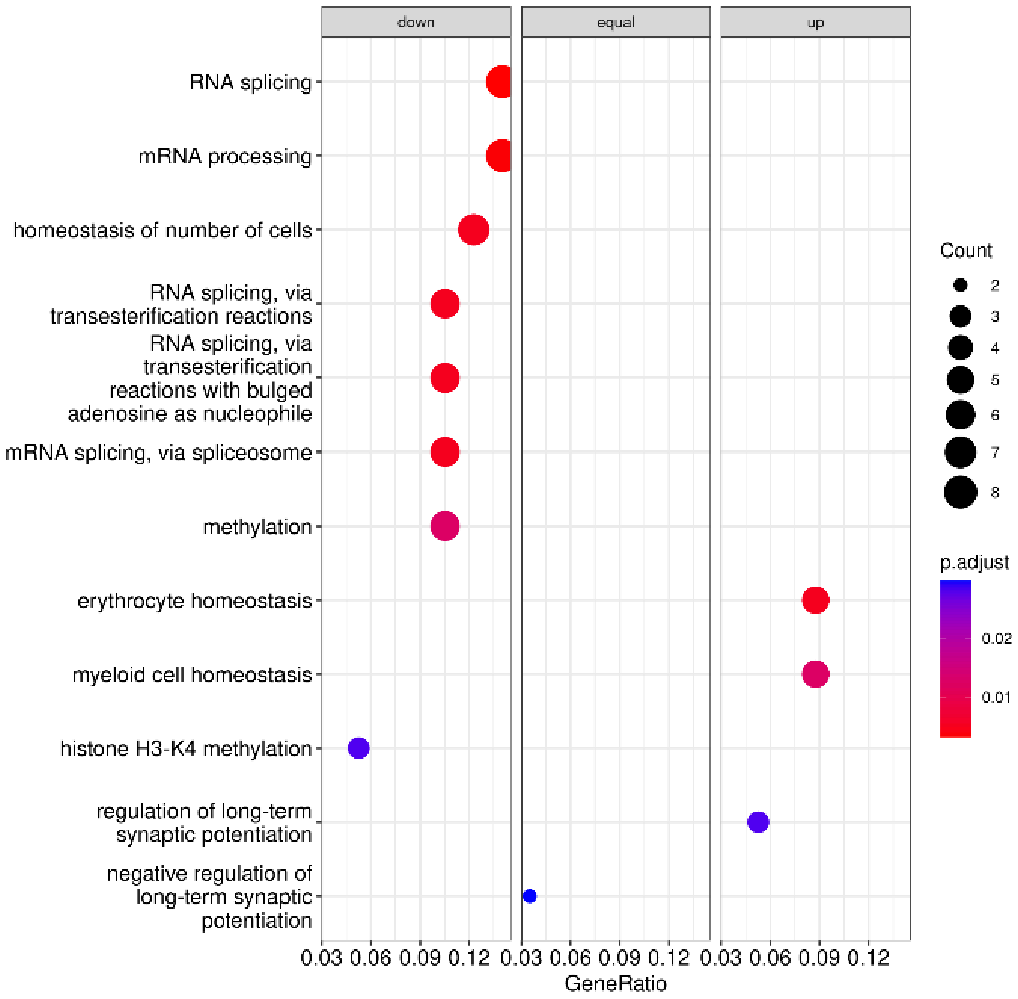

Fig. S6: **Dot plot visualization of the gene set enrichment analysis results for global male-specific differentially expressed genes (DEGs) in THY-Tau22 mice at 17 months of age**. The plot shows enriched Gene Ontology Biological Process (BP) terms (corresponding enriched Molecular Function (MF) terms are shown in Fig. S7). The size of each dot represents the count of genes in each enriched term, and the color scale indicates the adjusted p-value significance level. RNA processing and splicing pathways dominate the enriched processes. The gene ratio on the x-axis indicates the proportion of DEGs in each term relative to the total number of genes in that term.

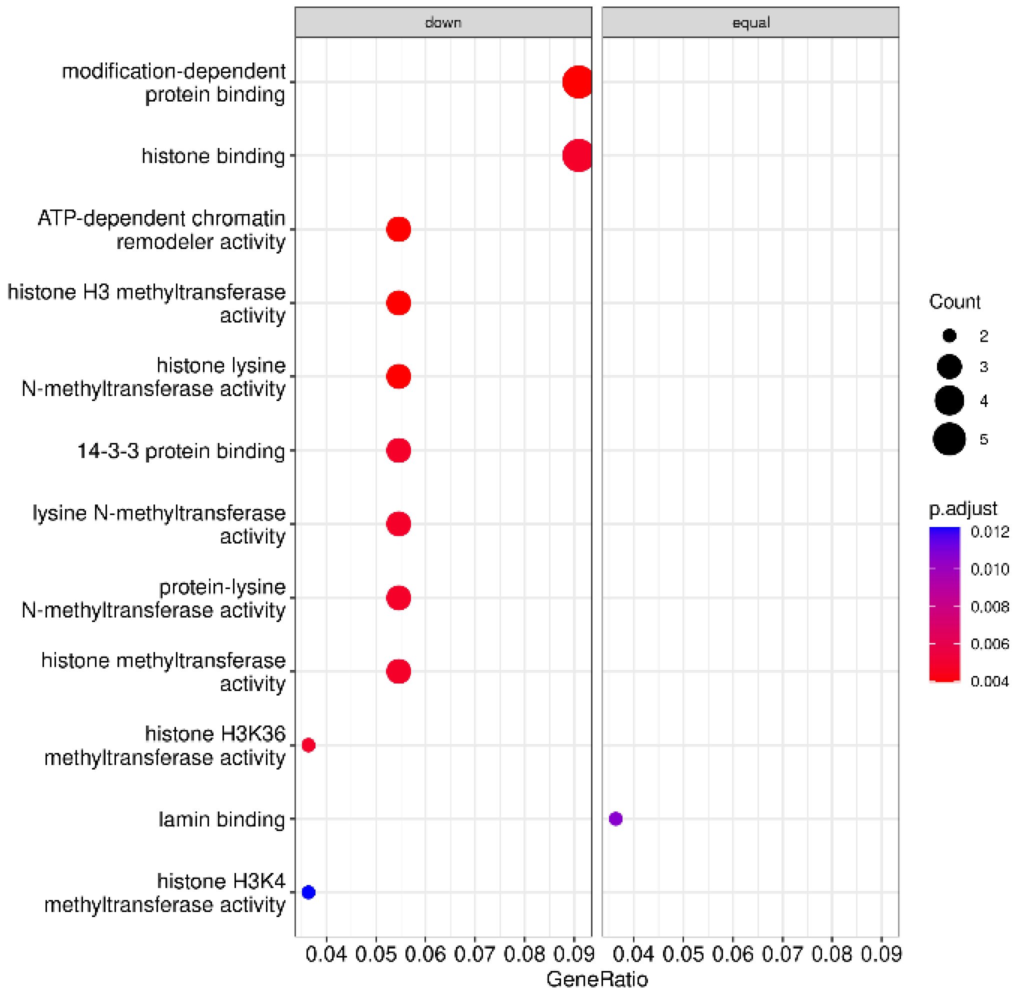

Fig. S7: **Dot plot visualization of the gene set enrichment analysis results for global male-specific differentially expressed genes (DEGs) in THY-Tau22 mice at 17 months of age**. The plot shows enriched Molecular Function (MF) terms (corresponding enriched Gene Ontology Biological Process (BP) terms are shown in Fig. S6). Left panel shows enriched Gene Ontology Biological Process (BP) terms, while the right panel shows enriched Molecular Function (MF) terms. The size of each dot represents the count of genes in each enriched term, and the color scale indicates the adjusted p-value significance level. Protein modification and histone-related functions are prominent among the enriched functions. The gene ratio on the x-axis indicates the proportion of DEGs in each term relative to the total number of genes in that term.

Global sex-dimorphic pathway enrichment (THY-Tau22, 17 months)

The pseudobulk gene set enrichment analysis of sex-dimorphic DEGs in THY-Tau22 mice at 17-months of age revealed distinct patterns of stress response and neurotransmitter signaling pathways (see Fig. 4). The most significantly enriched biological processes centered on cellular responses to salt, gliogenesis, and regulation of neuron death (adjusted $p<1e-4$), highlighting opposing transcriptional responses between sexes in pathways critical for cell survival and glial function. Several stress response pathways were also prominently enriched, including responses to oxidative stress, metal ions, and reactive oxygen species.

At the molecular function level, the most significant enrichment was found in ubiquitin-related processes and ligand binding (adjusted $p<1e-4$), followed by acetylcholine

receptor activities and regulatory functions. This suggests fundamental sex differences in protein degradation pathways and cholinergic signaling, both of which are known to be dysregulated in AD. The enrichment of both cellular stress responses and neurotransmitter signaling suggests that male and female mice show divergent adaptations to tau pathology in pathways central to cellular resilience and synaptic function. A comprehensive list of all enriched pathways and their statistical parameters is provided in Suppl. Table 5.

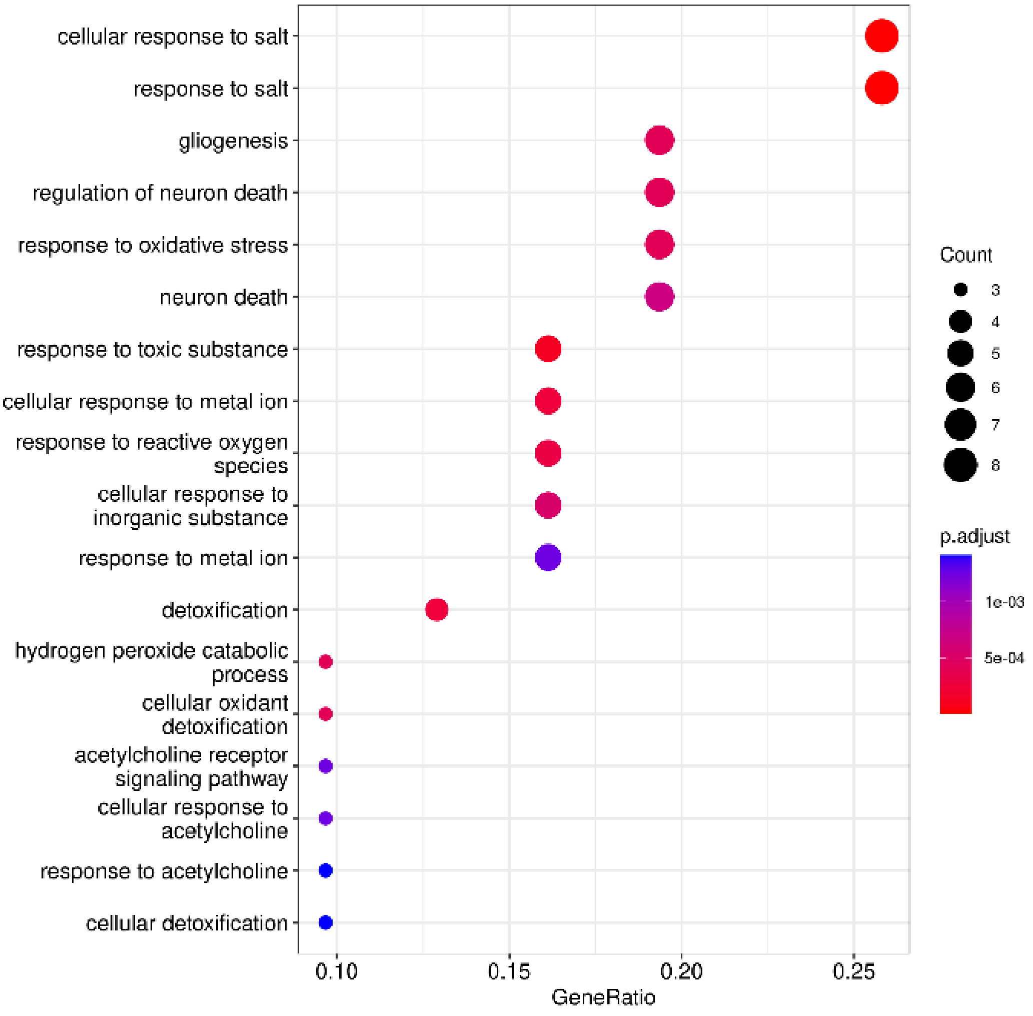

Fig. S8: Dot plot visualization of the gene set enrichment analysis results of global sex-dimorphic differentially expressed genes (DEGs) in THY-Tau22 mice at 17 months of age. The plot shows enriched Gene Ontology Biological Process (BP) terms (corresponding enriched Molecular Function (MF) terms are shown in Fig. S9). The size of each dot represents the count of genes in each enriched term, and the color scale indicates the adjusted p-value significance level (darker purple indicating higher significance). The enriched processes are dominated by stress responses, including cellular responses to salt, oxidative stress, and neuron death regulation. The gene ratio on the x-axis indicates the proportion of DEGs in each term relative to the total number of genes in that term

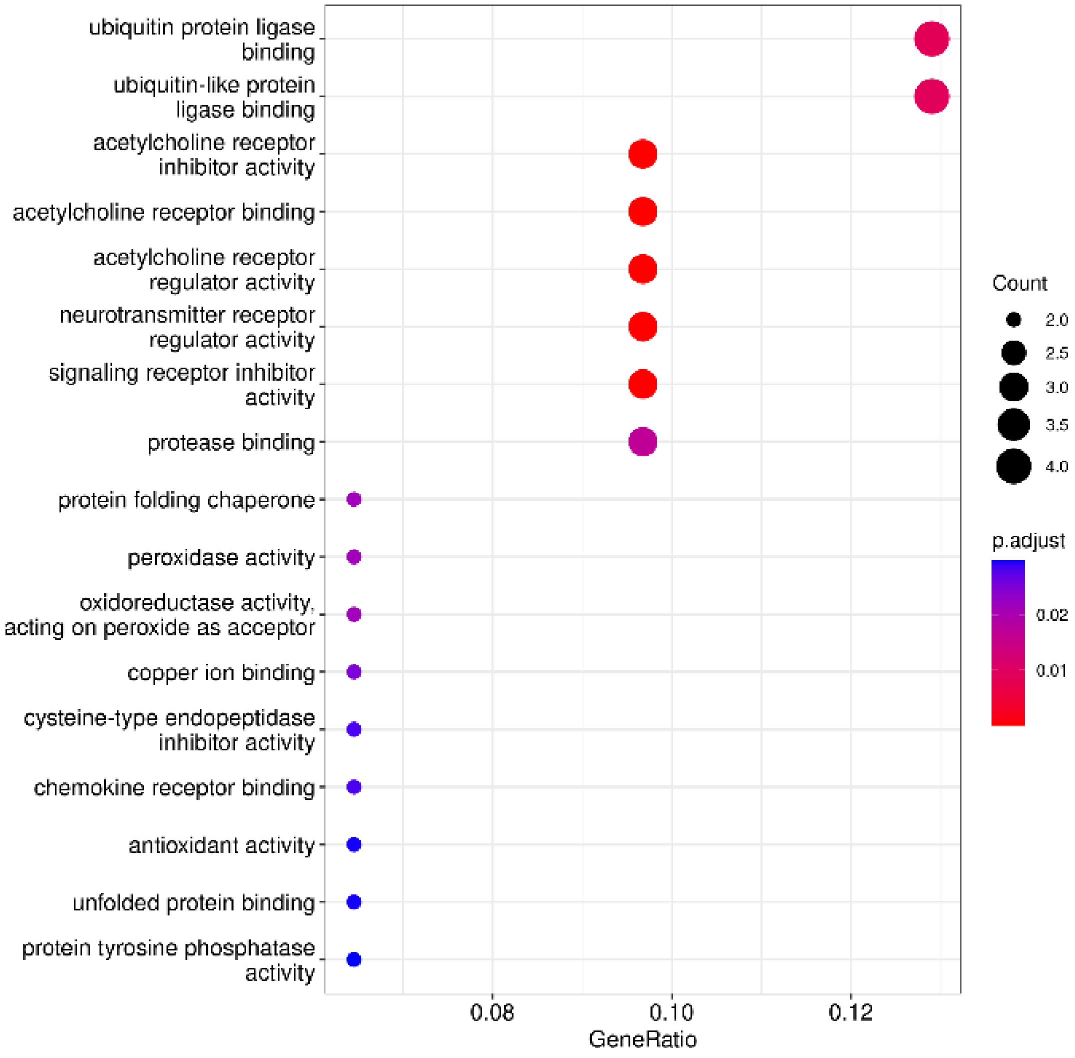

Fig. S9: Dot plot visualization of the gene set enrichment analysis results of global sex-dimorphic differentially expressed genes (DEGs) in THY-Tau22 mice at 17 months of age. The plot shows enriched Molecular Function (MF) terms (corresponding enriched Gene Ontology Biological Process (BP) terms are shown in Fig. S8). The size of each dot represents the count of genes in each enriched term, and the color scale indicates the adjusted p-value significance level (darker purple indicating higher significance). Strong enrichment is observed for ubiquitin-related processes and neurotransmitter receptor activities, particularly acetylcholine receptor pathways. The gene ratio on the x-axis indicates the proportion of DEGs in each term relative to the total number of genes in that term.

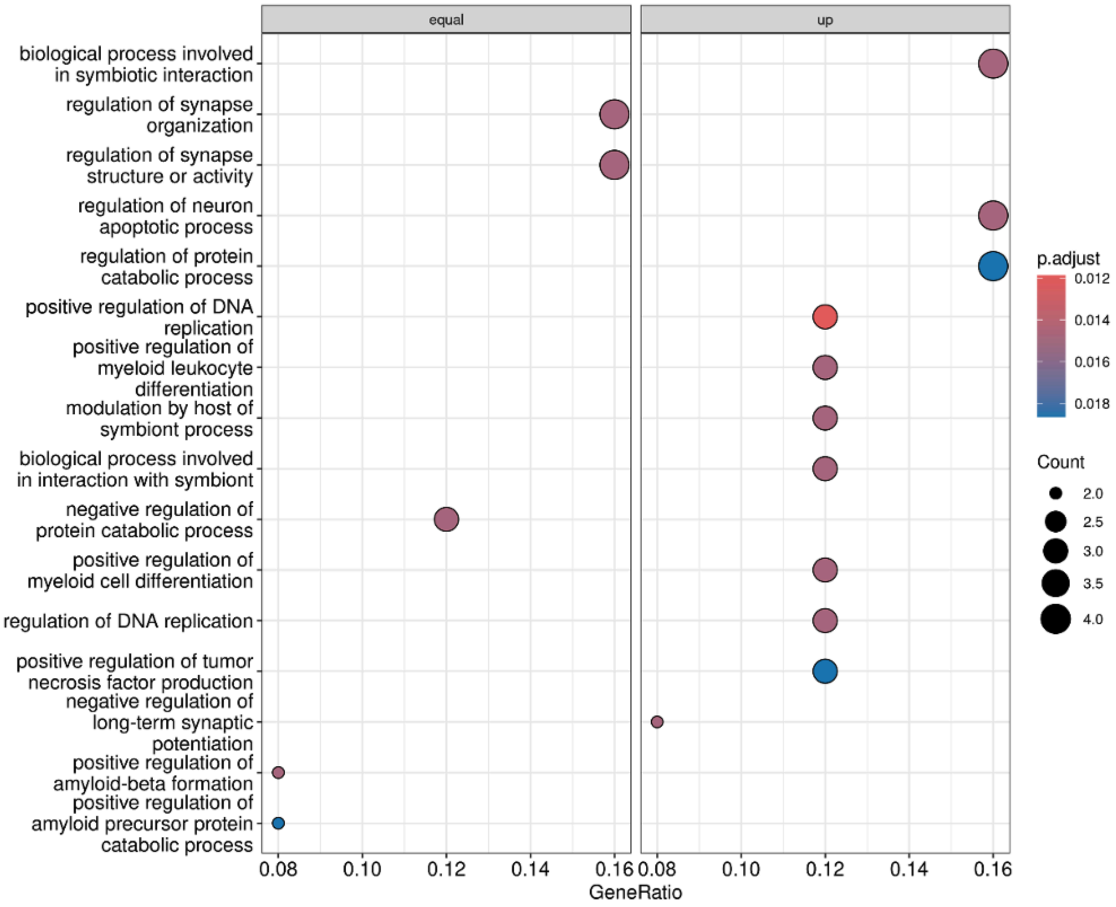

Fig. S10: Pathway enrichment analysis in microglia from 17-month-old THY-Tau22 mice. Dot plot showing enriched Gene Ontology Biological Processes. Dot size indicates number of differential genes in each pathway; color represents statistical significance (red=more significant). Whenever possible, each enriched pathway is classified into three regulatory patterns based on the predominant direction of change in its member genes (up, down, or equal). Gene ratio (x-axis) shows proportion of pathway genes differentially expressed. Only pathways with FDR<0.05 are shown.

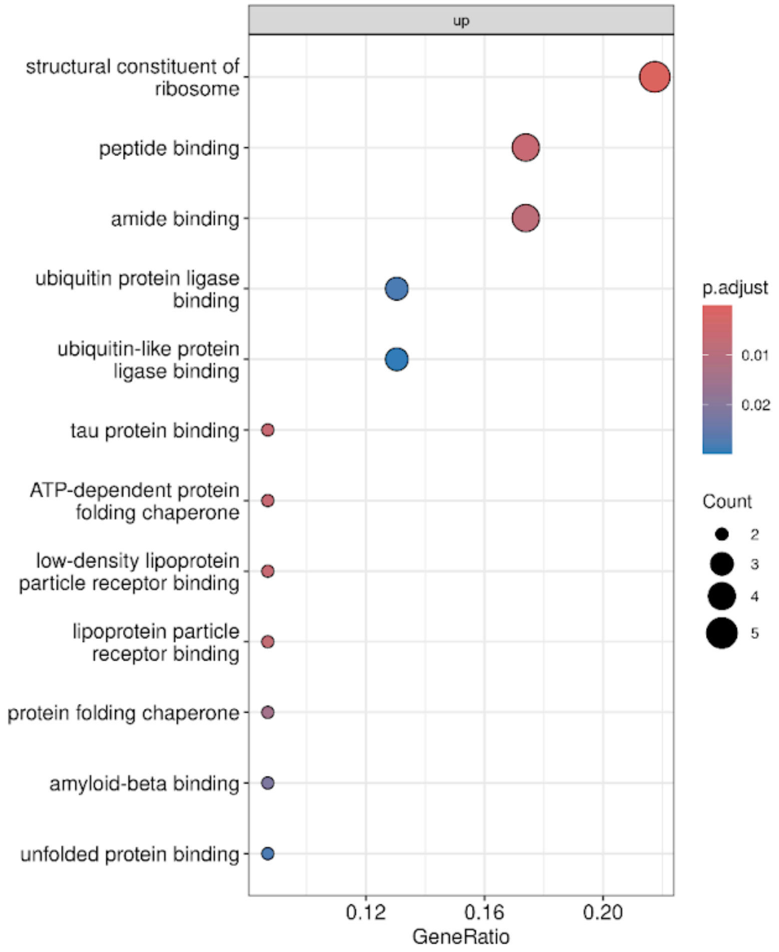

Fig. S11: Pathway enrichment analysis of sex-dependent microglial responses in THY-Tau22 mice at 17 months of age. Dot plot showing enriched Molecular Functions. Dot size indicates number of differential genes in each pathway; color represents statistical significance (red=more significant). Whenever possible, each enriched pathway is classified into three regulatory patterns based on the predominant direction of change in its member genes (up, down, or equal). Gene ratio (x-axis) shows proportion of pathway genes differentially expressed. Only pathways with FDR<0.05 are shown.

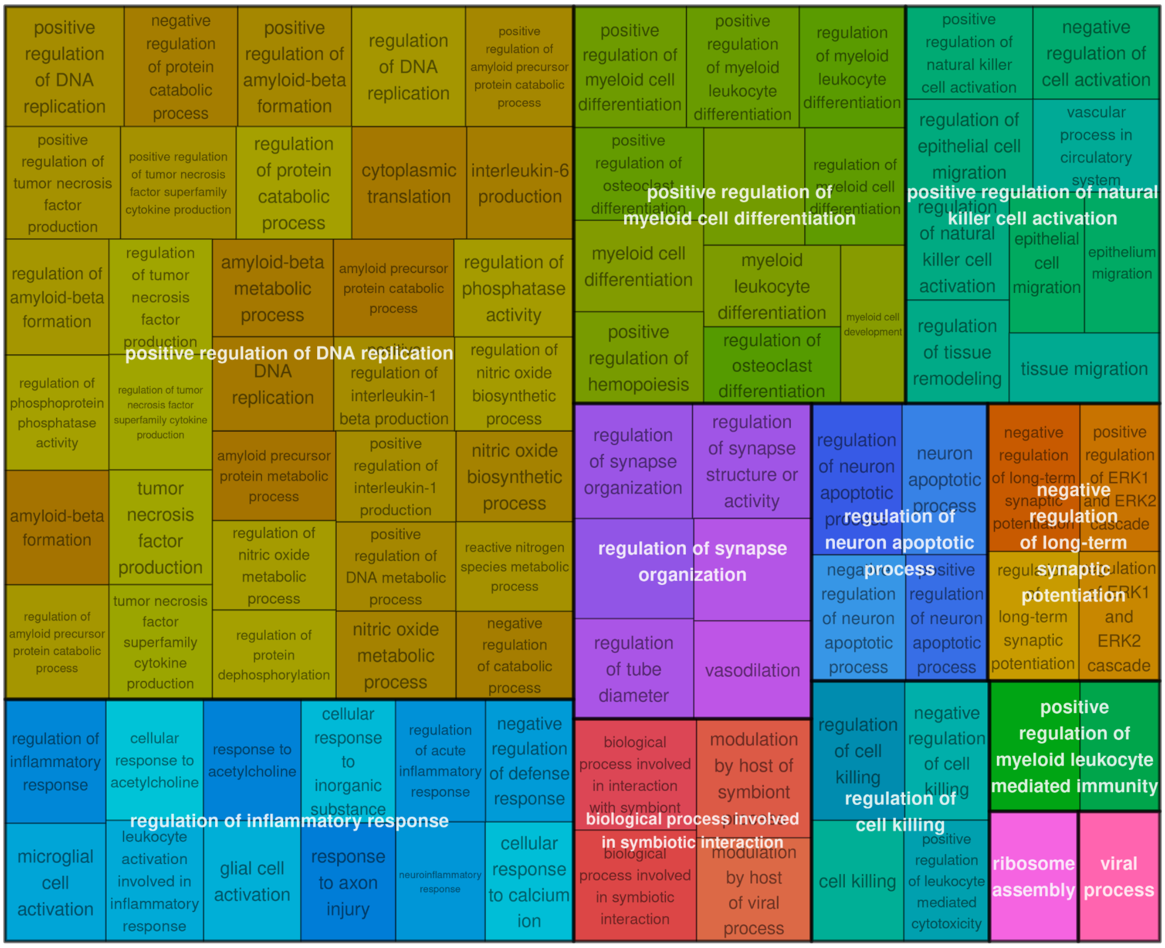

Fig. S12: Microglia pathway enrichment analysis (Biological Processes). Extended visualization of biological process enrichment for microglial DEGs, complementing Fig. 7 in the main text. Includes complete hierarchical organization of enriched terms.

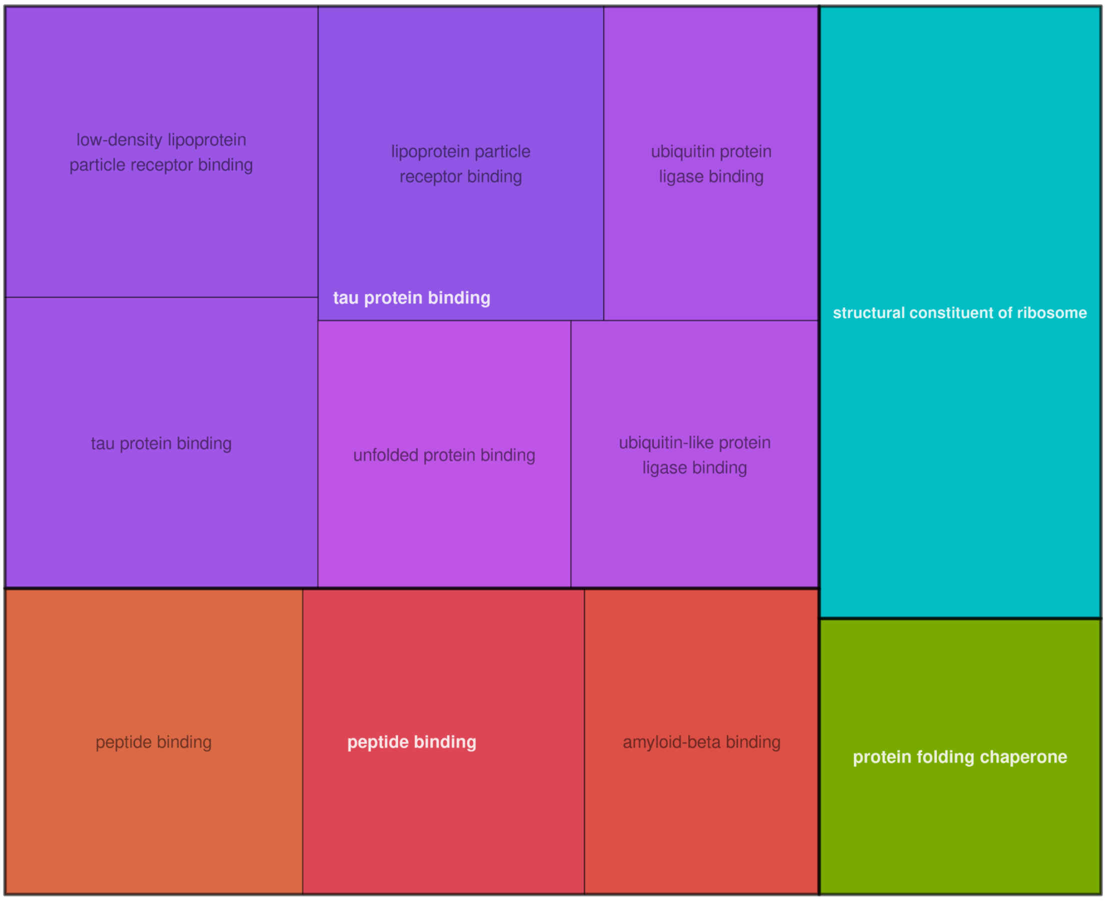

Fig. S13: Microglia pathway enrichment analysis (Molecular Function). Extended visualization of molecular function enrichment for microglial DEGs, complementing Fig. 7 in the main text. Includes complete hierarchical organization of enriched terms.

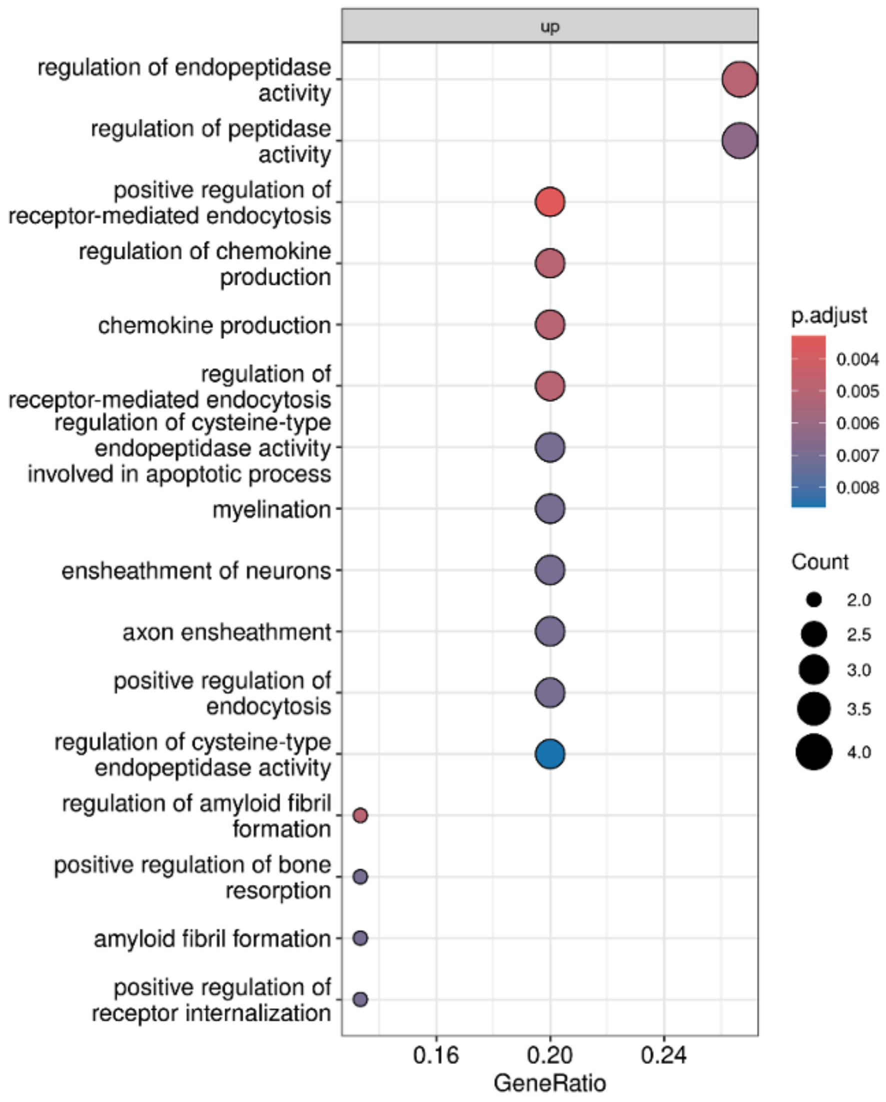

Fig. S14: Pathway enrichment analysis in oligodendrocytes from 17-month-old THY-Tau22 mice. Dot plot showing enriched Gene Ontology Biological Processes. Dot size indicates number of differential genes in each pathway; color represents statistical significance (red=more significant). Whenever possible, each enriched pathway is classified into three regulatory patterns based on the predominant direction of change in its member genes (up, down, or equal). Gene ratio (x-axis) shows proportion of pathway genes differentially expressed. Only pathways with FDR<0.05 are shown.

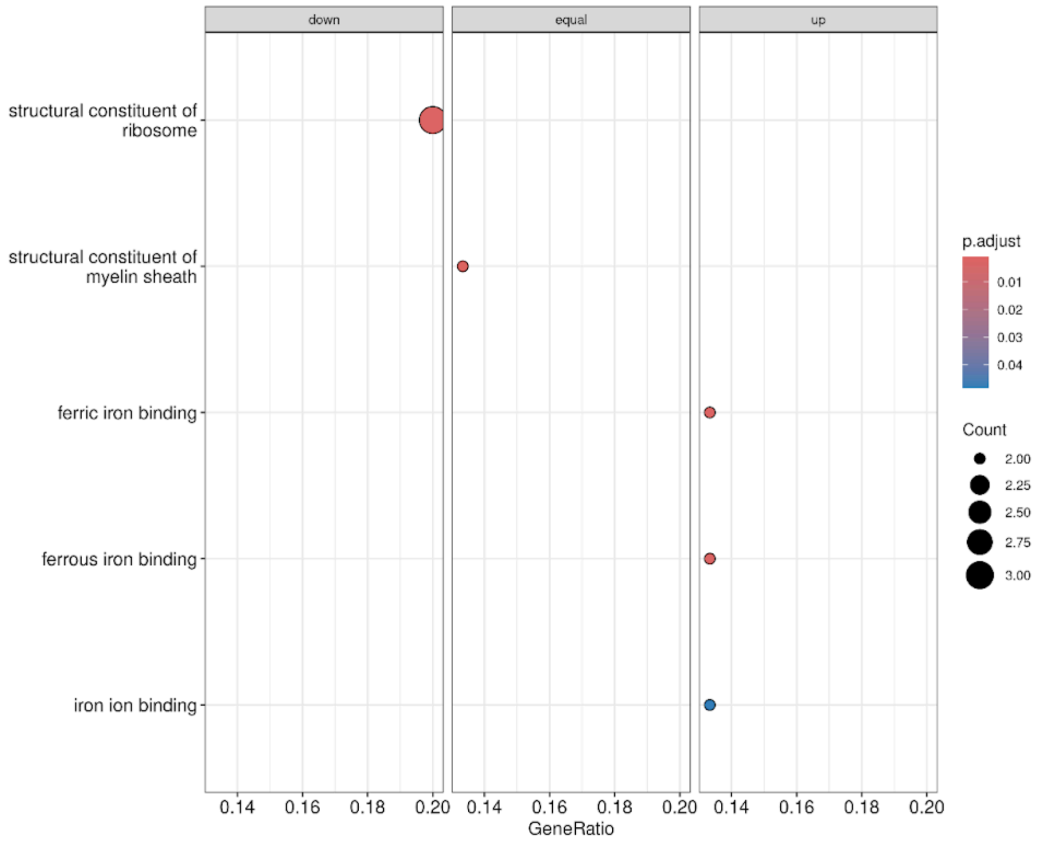

Fig. S15: Pathway enrichment analysis of oligodendrocyte responses in THY-Tau22 mice at 17 months of age. Dot plot showing enriched Molecular Functions. Dot size indicates number of differential genes in each pathway; color represents statistical significance (red=more significant). Whenever possible, each enriched pathway is classified into three regulatory patterns based on the predominant direction of change in its member genes (up, down, or equal). Gene ratio (x-axis) shows proportion of pathway genes differentially expressed. Only pathways with FDR<0.05 are shown.

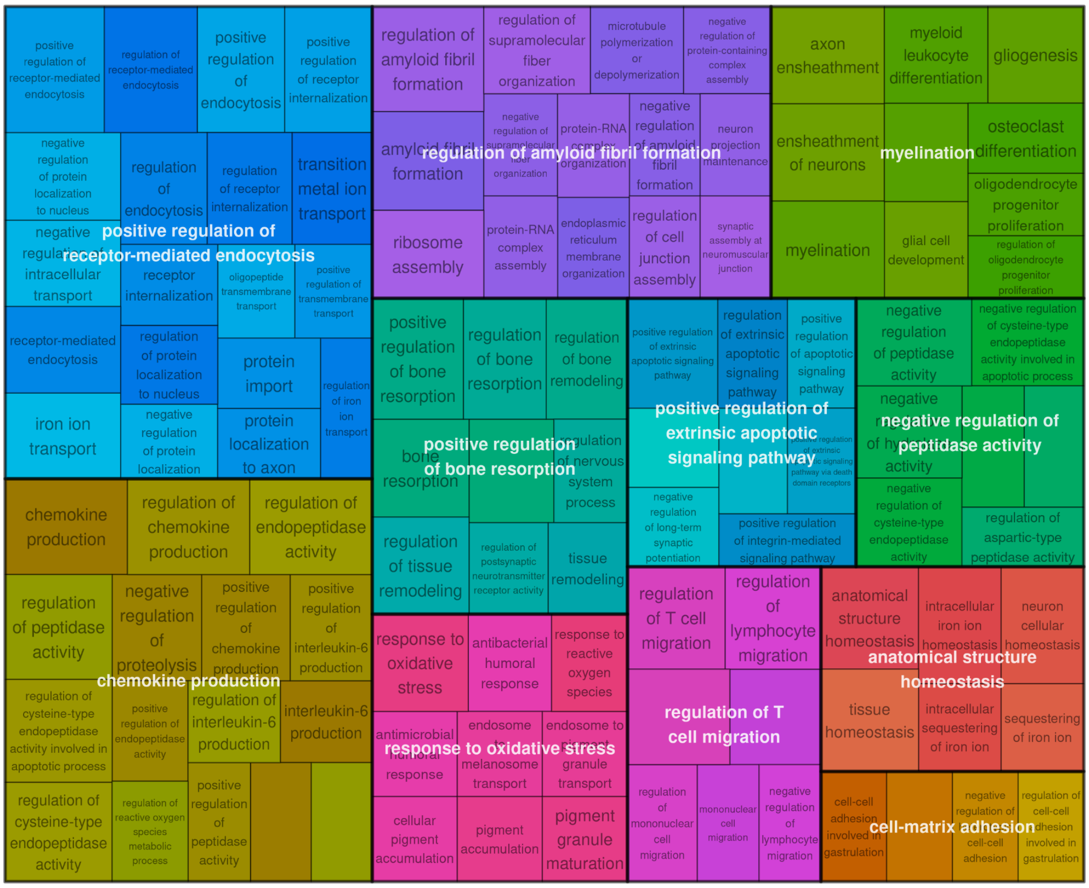

Fig. S16: Oligodendrocyte pathway enrichment analysis (Biological Process). Extended visualization of biological process enrichment for oligodendrocyte DEGs, complementing Fig. 8 in the main text. Includes complete hierarchical organization of enriched terms.

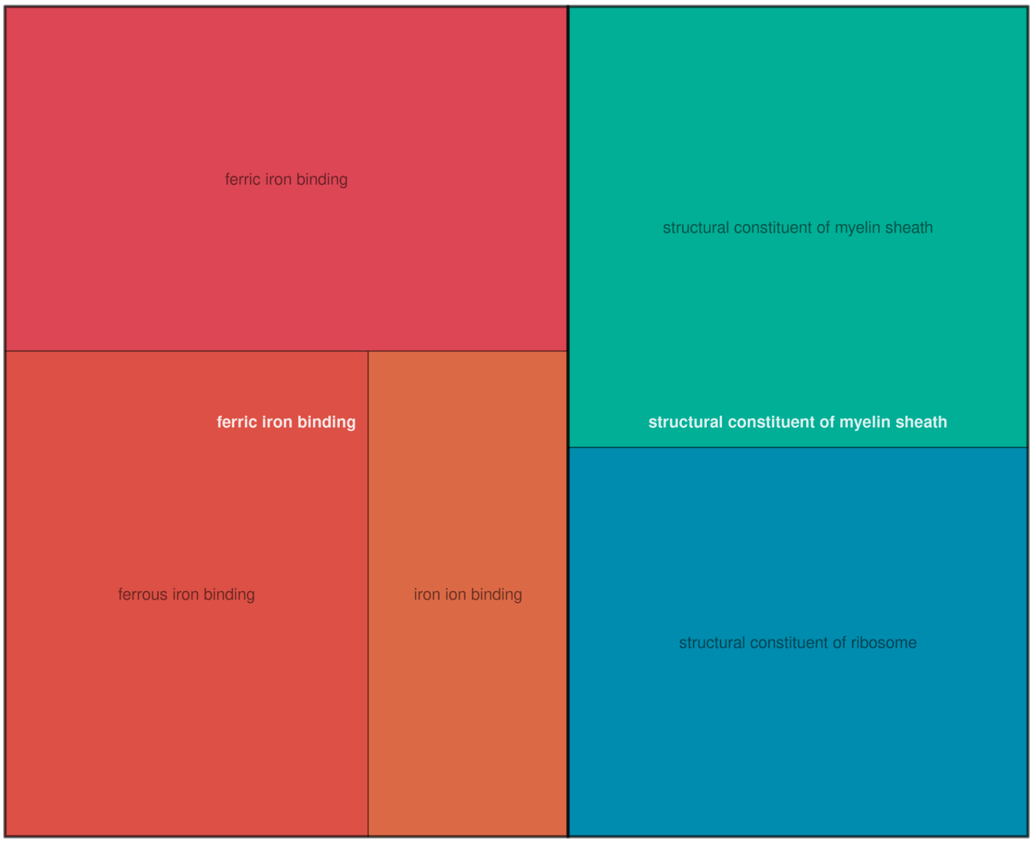

Fig. S17: Oligodendrocyte pathway enrichment analysis (Molecular Function). Extended visualization of molecular function enrichment for oligodendrocyte DEGs, complementing Fig. 8 in the main text. Includes complete hierarchical organization of enriched terms.

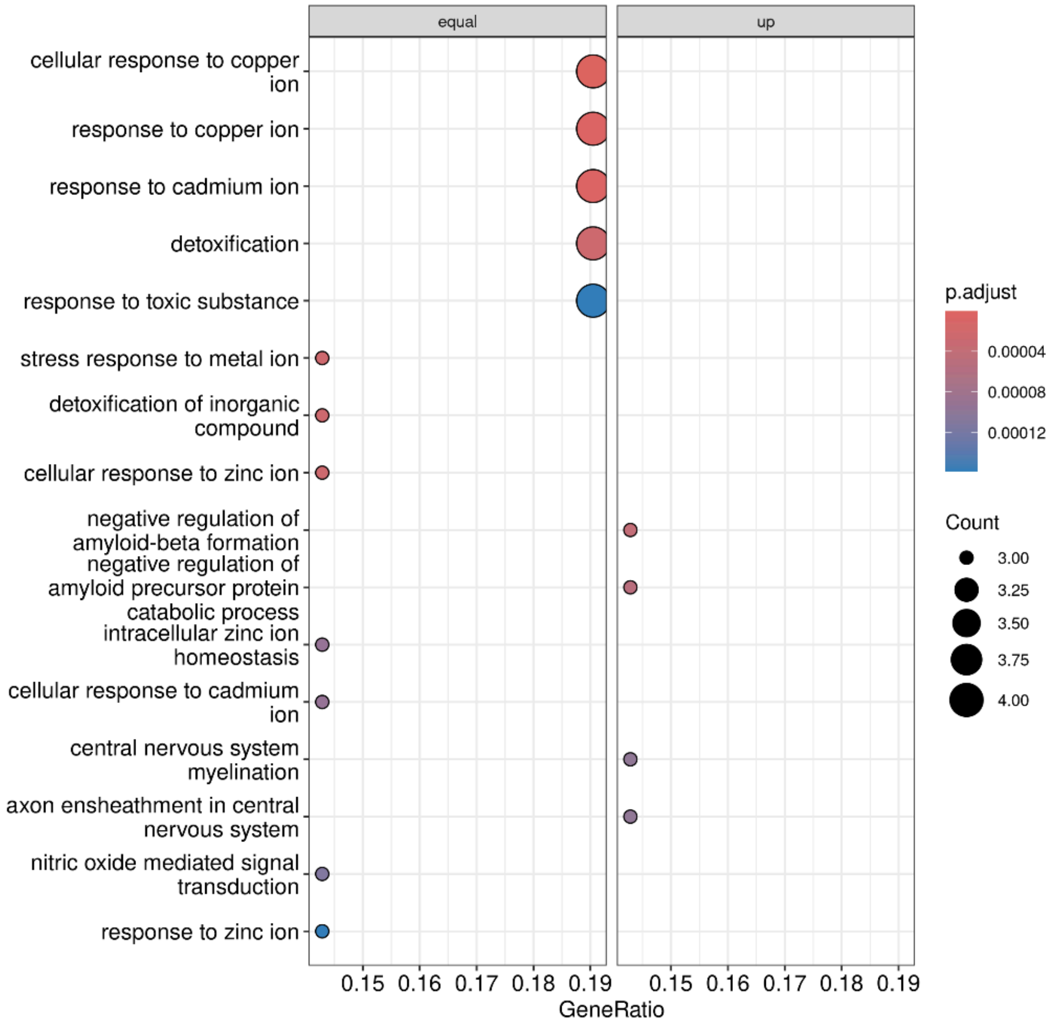

Fig. S18: Astrocyte pathway enrichment analysis. Visualization of Gene Ontology Biological Process (BP) enrichment for astrocyte DEGs. The dot plot shows the enriched pathways with gene ratios and statistical significance. The size of each dot represents the count of genes in each enriched term, and the color scale indicates the adjusted p-value significance level. Regulation of amyloid-beta formation and central nervous system myelination pathways dominate the enriched biological processes. The gene ratio on the x-axis indicates the proportion of DEGs in each term relative to the total number of genes in that term.

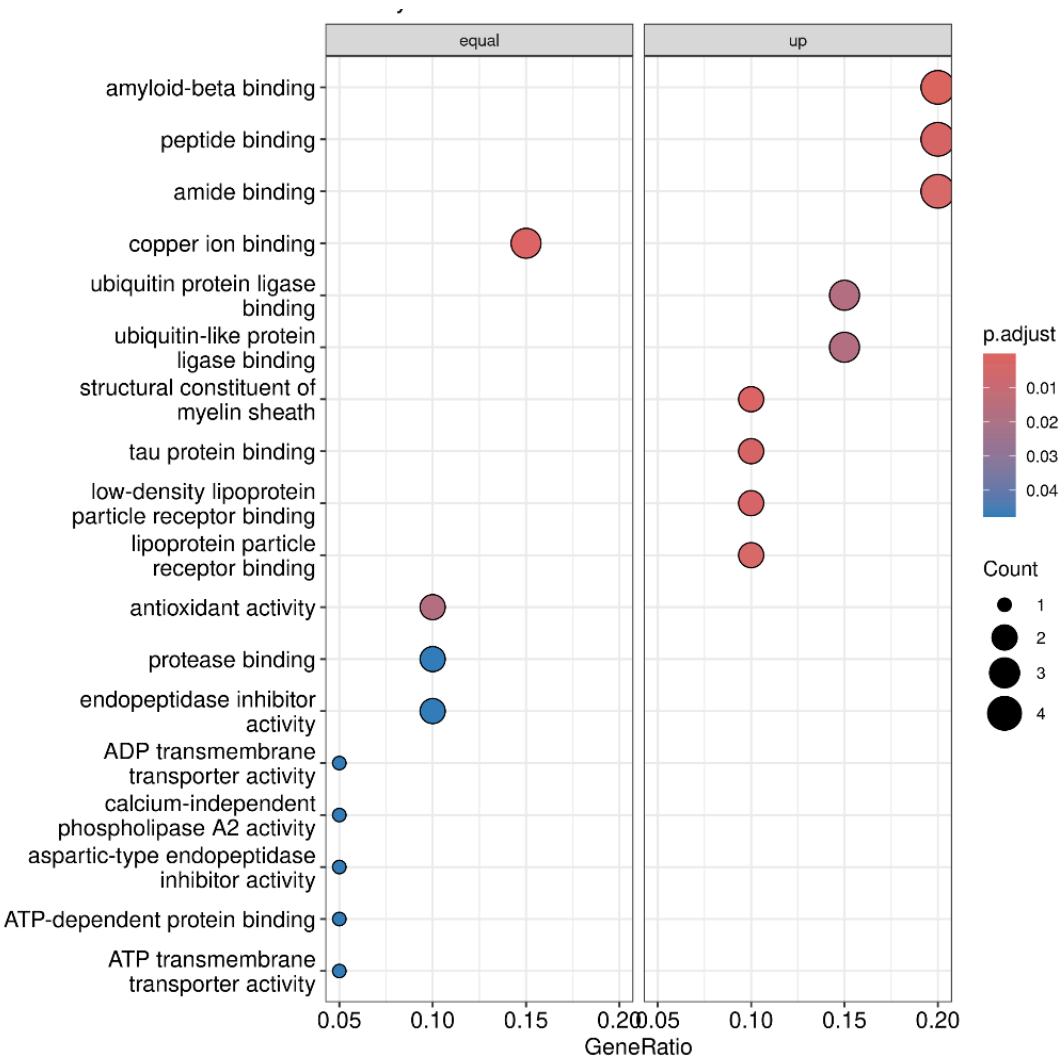

Fig. S19: Astrocyte pathway enrichment analysis. Visualization of Molecular Function (MF) enrichment for astrocyte DEGs. The dot plot shows the enriched pathways with gene ratios and statistical significance. The size of each dot represents the count of genes in each enriched term, and the color scale indicates the adjusted p-value significance level. Amyloid-beta and tau protein binding related functions are prominent among the enriched molecular functions. The gene ratio on the x-axis indicates the proportion of DEGs in each term relative to the total number of genes in that term.

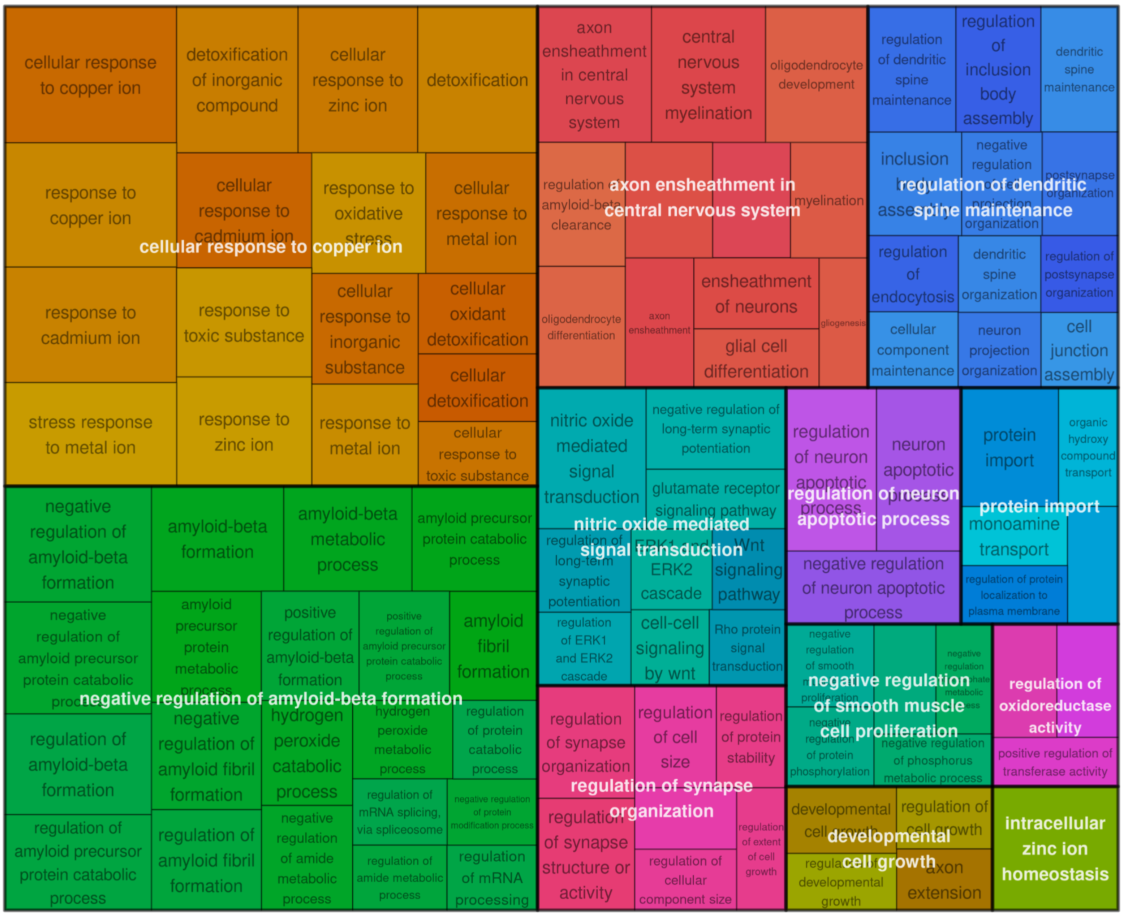

Fig. S20: Astrocyte pathway enrichment analysis (Biological Processes). Extended visualization of biological process enrichment for astrocyte DEGs. Includes complete hierarchical organization of enriched terms.

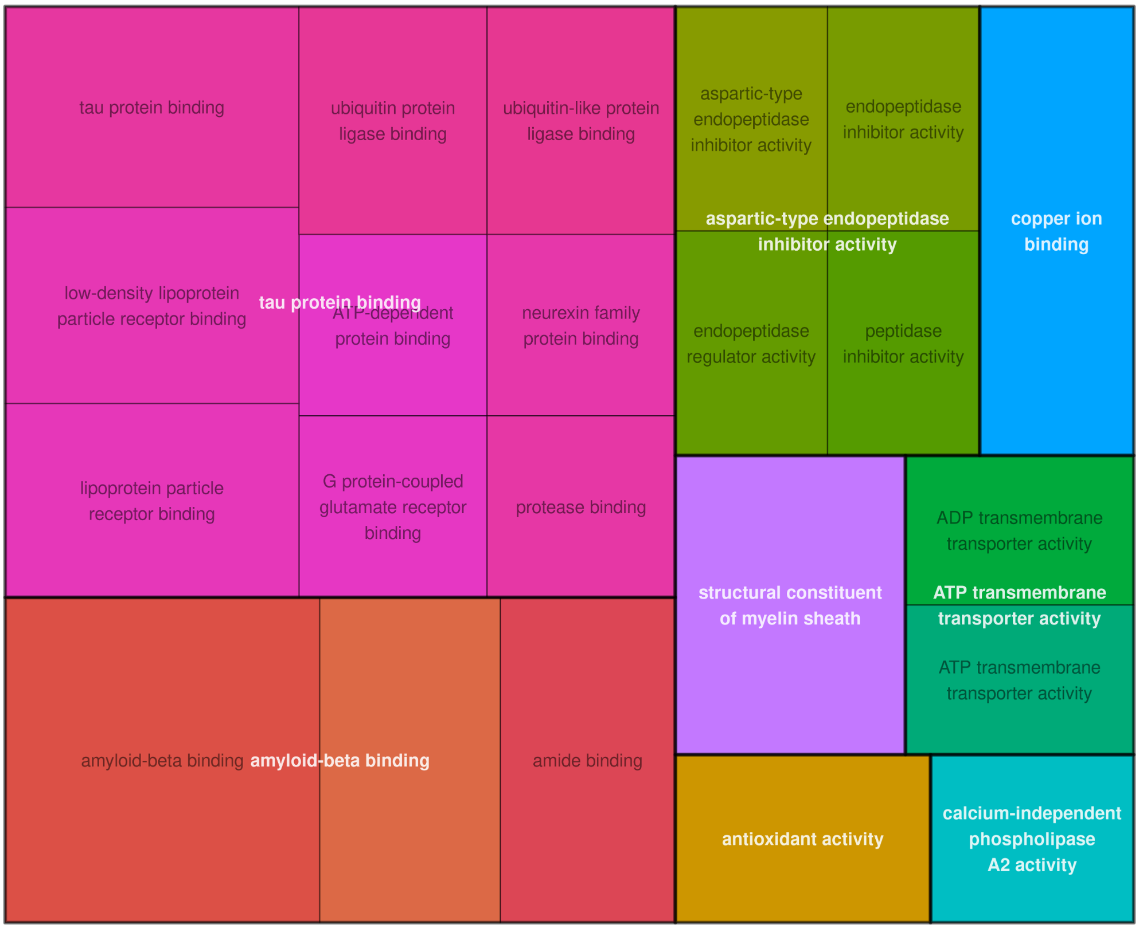

Fig. S21: Astrocyte pathway enrichment analysis (Molecular Functions). Extended visualization of molecular function enrichment for astrocyte DEGs. Includes complete hierarchical organization of enriched terms.

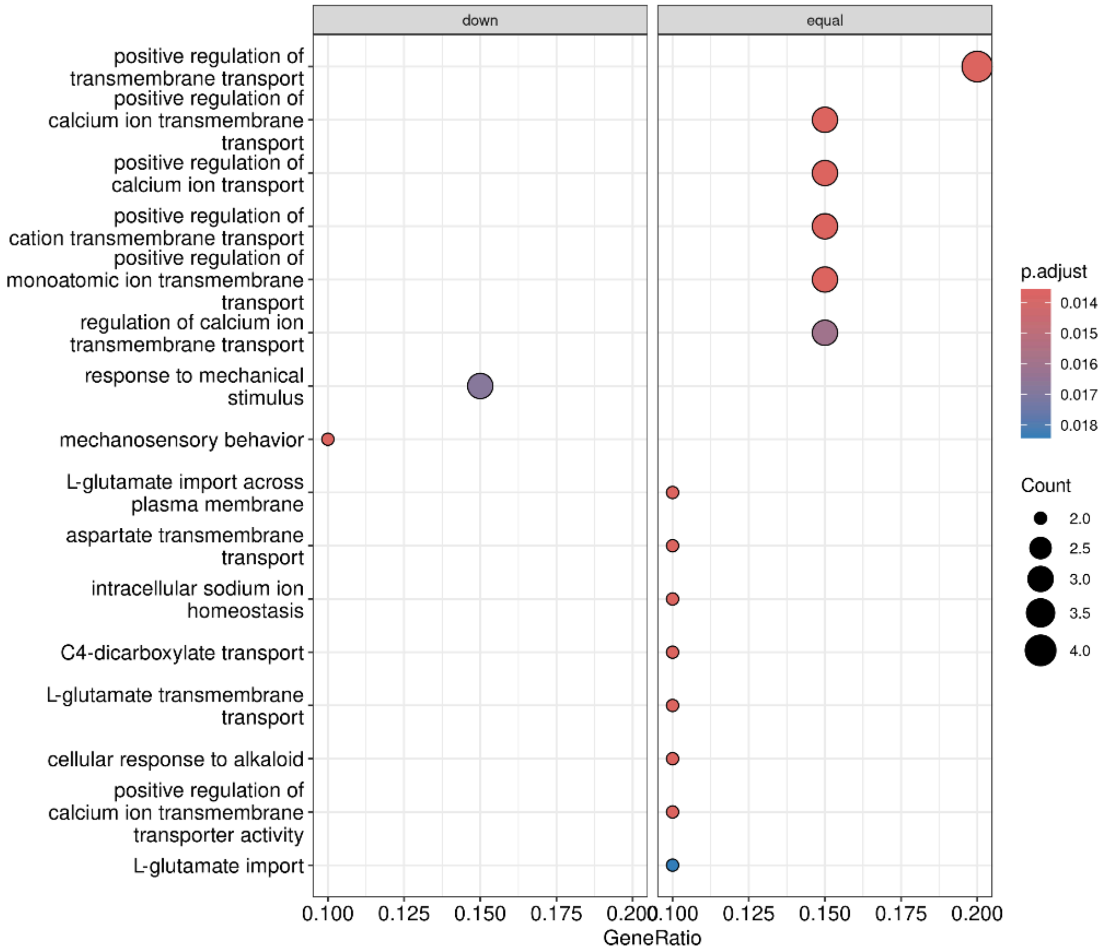

Fig. S22: Neuron pathway enrichment analysis. Visualization of Gene Ontology Biological Process (BP) enrichment for Neuron DEGs. The dot plot shows the enriched pathways with gene ratios and statistical significance. The size of each dot represents the count of genes in each enriched term, and the color scale indicates the adjusted p-value significance level. Transmembrane transport and sodium ion homeostasis pathways dominate the enriched biological processes. The gene ratio on the x-axis indicates the proportion of DEGs in each term relative to the total number of genes in that term.

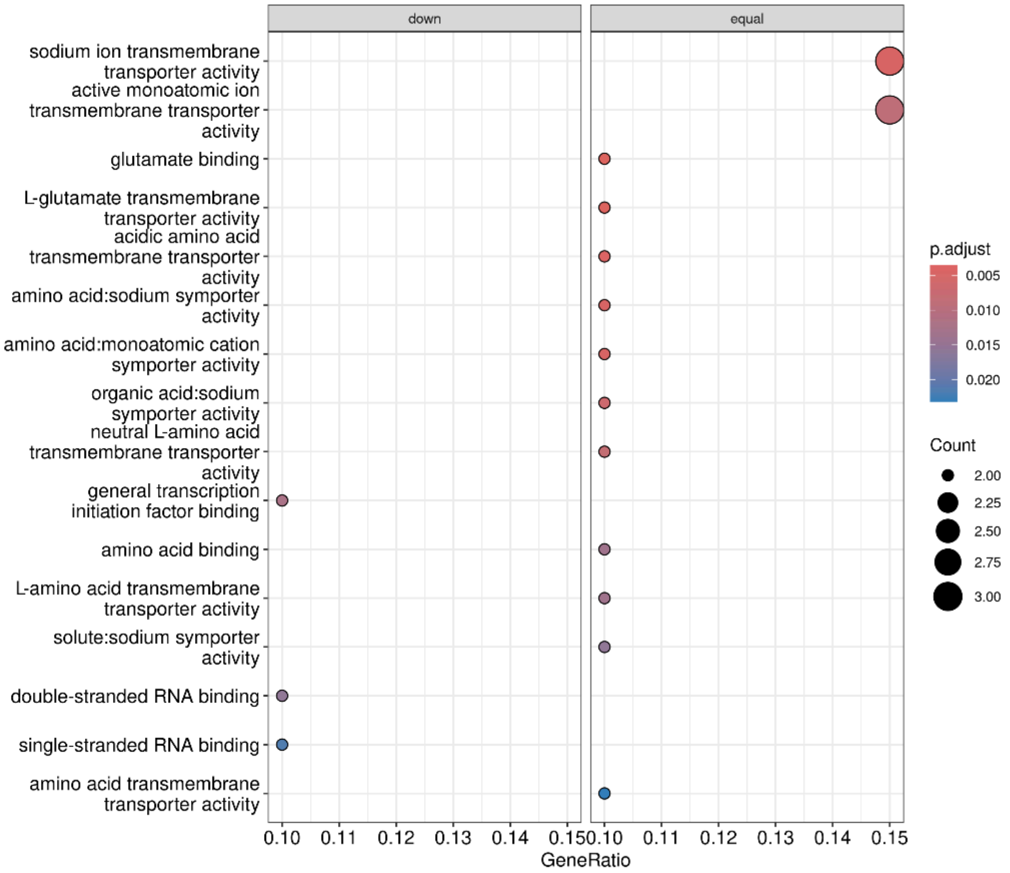

Fig. S23: Neuron pathway enrichment analysis. Visualization of Molecular Function (MF) enrichment for Neuron DEGs. The dot plot shows the enriched pathways with gene ratios and statistical significance. The size of each dot represents the count of genes in each enriched term, and the color scale indicates the adjusted p-value significance level. Transmembrane transporter activity and glutamate binding related functions are prominent among the enriched molecular functions. The gene ratio on the x-axis indicates the proportion of DEGs in each term relative to the total number of genes in that term.

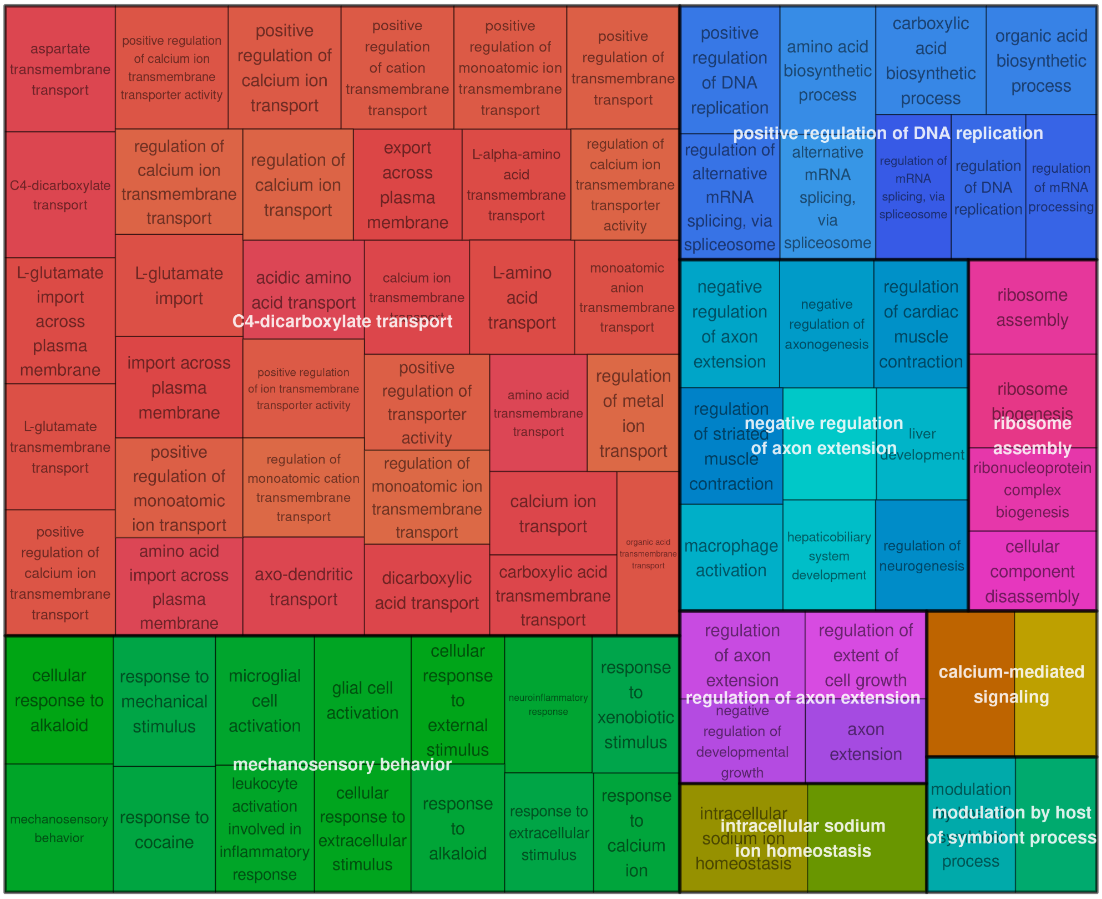

Fig. S24: Neuron pathway enrichment analysis (Biological Processes). Extended visualization of biological process enrichment for Neuron DEGs. Includes complete hierarchical organization of enriched terms.

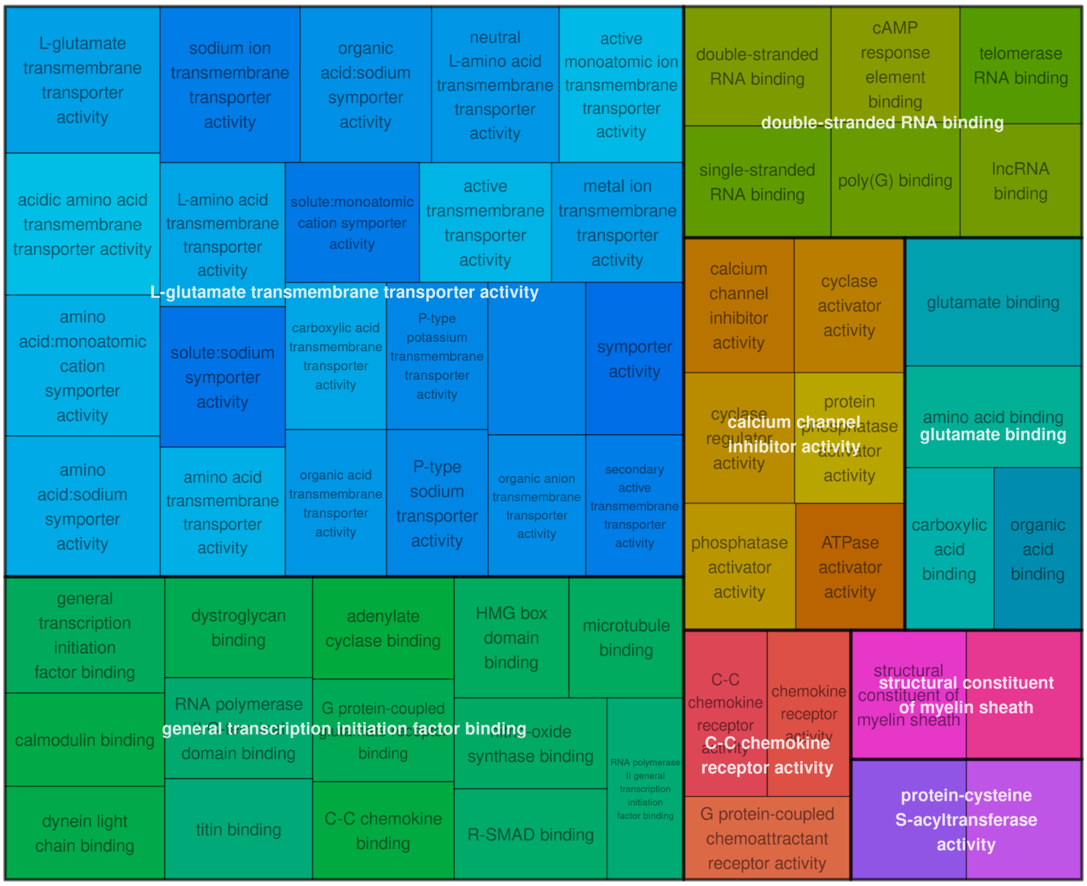

Fig. S25: Neuron pathway enrichment analysis (Molecular function). Extended visualization of molecular function enrichment for Neuron DEGs. Includes complete hierarchical organization of enriched terms.
